# ﻿Revision of Ardissoneaceae (Bacillariophyta, Mediophyceae) from Micronesian populations, with descriptions of two new genera, *Ardissoneopsis* and *Grunowago*, and new species in *Ardissonea*, *Synedrosphenia* and *Climacosphenia*

**DOI:** 10.3897/phytokeys.208.89913

**Published:** 2022-09-21

**Authors:** Christopher S. Lobban, Matt P. Ashworth, Terance Camacho, Daryl W. Lam, Edward C. Theriot

**Affiliations:** 1 Division of Natural Sciences, University of Guam, Mangilao, GU 96923, Guam, USA; 2 Department of Molecular Biosciences, The University of Texas at Austin, Austin, Texas, USA; 3 LSAMP Program, University of Guam, Mangilao, GU 96923, Guam, USA; 4 Department of Biological Sciences, University of Alabama, Tuscaloosa, AL 35487, USA; 5 Section of Integrative Biology, The University of Texas at Austin, Austin, TX, USA

**Keywords:** Ardissoneales, biodiversity, coral reefs, Grunow, Mediophyceae, systematics, Toxariales, Western Pacific

## Abstract

*Ardissonea* was resurrected from *Synedra* in 1986 and was included as a genus by Round, Crawford and Mann (“The Diatoms”) in its own Family and Order. They commented that there might be several genera involved since the type species of the genus possesses a double-walled structure and other taxa placed in *Ardissonea* have only a single-walled structure. Two other genera of “big sticks,” *Toxarium* and *Climacosphenia*, were placed in their own Families and Orders but share many characters with Ardissoneaceae, especially growth from a bifacial annulus. Eighteen taxa (11 new species) from Micronesia were compared with the literature and remnant material from Grunow’s Honduras *Sargassum* sample to address the concepts of *Ardissonea* and Ardissoneaceae. Phylogenetic and morphological analyses showed three clades within *Ardissonea* sensu lato: *Ardissonea* emend. for the double-walled taxa, *Synedrosphenia* emend. and *Ardissoneopsis***gen. nov.** for single-walled taxa. New species include *Ardissoneadensistriata***sp. nov**.; *Synedrospheniabikarensis***sp. nov**., *S.licmophoropsis***sp. nov.**, *S.parva***sp. nov.**, and *S.recta***sp. nov**.; *Ardissoneopsisfulgicans***sp. nov.**, *A.appressata***sp. nov.**, and *A.gracilis***sp. nov.** Transfers include *Synedrospheniacrystallina***comb. nov**. and *S.fulgens***comb. nov**. *Synedraundosa*, seen for the first time in SEM in Grunow’s material, is transferred to *Ardissoneopsisundosa***comb. nov.** Three more genera have similar structure: *Toxarium*, *Climacosphenia* and *Grunowago***gen. nov.**, erected for *Synedrabacillaris* and a lanceolate species, *G.pacifica***sp. nov.** Morphological characters of *Toxarium* in our region support separation of *Toxariumhennedyanum* and *T.undulatum* and suggest additional species here and elsewhere. *Climacospheniamoniligera* was not found but we clarify its characters based on the literature and distinguish *C.soulonalis***sp. nov.** from it. *Climacospheniaelongata* and a very long, slender *C.elegantissima***sp. nov.**, previously identified as *C.elongata*, were present along with *C.scimiter*. Morphological and molecular phylogenetics strongly suggested that all these genera belong in one family and we propose to include them in the Ardissoneacae and to reinstate the Order Ardissoneales Round.

## ﻿Introduction

Bilaterally symmetrical, stick-like diatoms, 300–1300 µm long, are common and sometimes abundant epiphytes on seaweeds of coral reefs. Some have bilateral growth of the valve (“pennate” diatoms) and are in the Class Fragilariophyceae, including *Stricosus* Sabir & E.C. Theriot, 2018 and longer species of *Hyalosynedra* D.M. Williams & F.E. Round, 1986 (Sabir et al. 2018). However, several common genera have valve growth from a bifacial annulus, at or near the edge of the valve face, forming striae internally and externally (Mann 1984; Kaczmarska et al. 2020) and were moved to Class Mediophyceae (bi- to multipolar diatoms) (Medlin et al. 2008) and are presently placed in four families in the Order Toxariales (Cox 2015). All are non-pennate but emerge in 7-gene phylogenetic trees in a clade sister to the pennate diatoms (Kooistra et al. 2003; Medlin et al. 2008; Theriot et al. 2015; Medlin and Desdevises 2020). Previously Round et al. (1990) had placed four genera—*Ardissonea* De Notaris, 1870 in De Notaris and Baglietto (1871), *Toxarium* J.W. Bailey, 1854, and *Climacosphenia* Ehrenberg, 1843, plus *Synedrosphenia* (H. Peragallo) Azpeitia, 1911—in three Families, each in a separate Order in Fragilariophyceae. The sexual reproduction of *A.crystallina* (Agardh) Grunow ex De Toni, 1892 has similarities to that of Fragilariophyceae in being isogamous (Davidovich et al. 2017) and in morphology of its auxospores (Kaczmarska et al. 2018), yet the valve morphogenesis reinforces its position among Mediophyceae (Kaczmarska et al. 2020). Gliding motility is present in these diatoms, even though they lack a raphe—the structure usually associated diatom motility—and even though they are habitually sessile on mucilage stalks. Gliding was demonstrated in *A.crystallina*, with mucilage secretion apparently occurring via a deep groove between the valve and valvocopula at each end of the cell (Pickett-Heaps et al. 1991), and in *Toxarium*, which has a similar apical structure (Kooistra et al. 2003).

Each genus in the Toxariales has a confused taxonomic history that has not yet been addressed with electron microscopy and several issues need to be resolved. The genus *Ardissonea*, subsumed as a subgenus of *Synedra* Ehrenberg, 1830 and declared by Hustedt (1931–1959: 238) to be a “difficult group of … *Synedra*,” was resurrected and emended by Poulin et al. (1986) based on SEM study, and the spelling of the name corrected (from *Ardissonia*) by Round et al. (1990). The generitype *Ardissonearobusta* (Ralfs) De Notaris, 1871 was not among the species studied by Poulin et al. (1986) but apparently was the species Round et al. (1990) used to illustrate the structure of the genus. The structure of *A.formosa* (Hantzsch) Grunow ex De Toni, 1892, studied by Sullivan and Wear (1995), differs significantly from the taxa studied by Poulin et al. (1986, 1987) [*A.crystallina* and *A.fulgens* (Greville) Grunow ex De Toni, 1892] in having a double wall and unperforated girdle bands. On the other hand, the genus *Synedrosphenia*, as illustrated by Round et al. (1990), while having a structure very similar to *Ardissonea* species shown by Poulin et al. (1986), has heteropolar valves and was classified by Round et al. (1990) with the heteropolar genus *Climacosphenia* in the Climacospheniaceae. Round et al. (1990: 420) commented about *Ardissonea* that, “It may prove best to place the non-chambered species in yet another genus,” but this has not yet been done. The definition of the genus has become confused because while Poulin et al. (1986) based their emendation on *A.crystallina* and characterized the transapical costae in these single-walled taxa as providing “chambering,” Round et al. (1990) based their description on *A.robusta*, the generitype, and thus included the internal plate and complete chambering of the valve as a character. Kanjer et al. (2021) regarded *A.fulgens* as “differing only slightly” from the generitype in lacking the internal plate.

The genera *Toxarium* and *Climacosphenia* differ from the others in different ways. *Climacosphenia* was formerly classified alongside *Licmophora*, while *Toxarium* was in *Synedra* (Peragallo and Peragallo 1897–1908; Hustedt 1931–1959). *Toxarium* is defined by the presence of scattered areolae within the annulus and *Climacosphenia* by the presence of craticular bars on the valvocopula. Both have a history of debate over whether there are one or two species of each, distinguished only by cell shape. This led to an assumption that there are only two species in each to choose from, i.e., *Toxariumundulatum* J.W. Bailey, 1854 (generitype) and *T.hennedyanum* (Gregory) Pelletan, 1889; *Climacospheniamoniligera* Ehrenberg, 1843 (generitype) and *C.elongata* J.W. Bailey, 1854.

Finally, one seemingly less similar taxon that occurs among *Ardissonea*, *Toxarium* and *Climacosphenia* spp. in our flora has been reported as *Synedrabacillaris* (Grunow) Hustedt, 1932 (= Synedracrystallinavar.bacillaris Grunow, 1880) (Lobban et al. 2012); this species has a distinct central costa and apparently lacks an annulus. Even though many marine species formerly in *Synedra* have been removed to new genera (Williams and Round 1986), *S.bacillaris* has yet to be revised, despite Sullivan and Wear’s (1995) observations.

The initial objective of this study was to describe several new species of *Ardissonea*, but in our morphological study for taxonomy and floristics we were led to address the systematics of the whole group. We here present descriptions of the taxa in Micronesia, including new genera and new species, plus some related species from Grunow’s (1867, 1877) Honduras materials. We analyze sequence data and morphological characters, make the case for including *Toxarium* and *Climacosphenia* in Ardissoneacae, within a reinstated Ardissoneales Round, and, finally, make nomenclatural changes that reflect our conclusions. These include transfer of single-walled *Ardissonea* species into *Synedrosphenia* and a new genus *Ardissoneopsis*, amending the generic descriptions as needed, and a new genus for *Synedrabacillaris*.

## ﻿Materials and methods

### ﻿Sample collection and imaging

Samples from our extensive collection of benthic diatoms from Micronesian coral reef habitats, preserved and prepared using the protocols standard in the U. Guam diatoms laboratory (Lobban 2021b), were surveyed for taxa of interest. Origins of the samples used in this study are listed in Table 1. Some material was observed alive, and often then transferred as a whole mount onto a filter, dried, and mounted for SEM; this enabled us to study the girdle bands in situ. In addition, we were able to observe the rich material in Grunow’s Honduras sample, collected by A. Lindig ca. 1860 and published in Grunow (1867, 1877). They were unmounted remnants of acid cleaned material he used for preparation of slides of other species; these were listed in Lobban et al. (2021a, table 1).

**Table 1.** Collection localities of specimens mentioned in this study.

Country	Entity (Island)	Municipality (“State”)	Locality name	Locality code	GPS coordinates
United States of America	Guam	Yona Municipality	UOG Marine Laboratory	GU7	13.428°N, 144.799°E
United States of America	Guam	Inarajan Municipality	Saluglula Pools, Inarajan	GU21	13.271°N, 144.748°E
United States of America	Guam	Hagatna Municipality	Agana Boat Basin	GU26	13.478°N, 144.749°E
United States of America	Guam	Santa Rita Municipality	GabGab Reef	GU44	13.443°N, 144.643°E
United States of America	Guam	Piti Municipality	Scuba (Outhouse) Beach	GU52	13.464°N, 144.656°E
United States of America	Guam	Umatac Municipality	Nathan’s Dent	GU54	13.336°N, 144.641°E
United States of America	Guam	Agat Municipality	“Pete’s Reef”	GU55	13.347°N, 144.639°E
United States of America	Guam	Merizo Municipality	Achang mangroves	GU58	13.249°N, 144.697°E
United States of America	Guam	Merizo Municipality	Cocos West @ MDA buoy	GU56	13.252°N, 144.648°E
United States of America	Guam	Santa Rita Municipality	GabGab Reef II	GU66	13.444°N, 144.644°E
United States of America	Guam	Santa Rita Municipality	Western Shoals, Apra Habor	GU68	13.451°N, 144.656°E
United States of America	Guam	Agat Municipality	Agat Reef nr Coral Gardens	GU75	13.357°N, 144.645°E
United States of America	Guam	Santa Rita Municipality	Vecky’s Reef	GU76	13.449°N, 144.625°E
United States of America	CNMI	Saipan Municipality	American Memorial Park	SPN2022-1-4	15.218°N, 145.721°E
Federated States of Micronesia	Yap	Weloy Municipality	Nimpal MPA reef	Y36	9.542°N, 138.084°E
Federated States of Micronesia	Yap	Weloy Municipality	Nimpal MPA reef	Y37	9.542°N, 138.082°E
Federated States of Micronesia	Yap	Tomil Municipality	Tagireeng Channel	Y39	9.562°N, 138.145°E
Federated States of Micronesia	Yap	Tomil Municipality	Peelaek Corner	Y42	9.515°N, 138.179°E
Federated States of Micronesia	Chuuk	Weno Municipality	Eastern Passage, Moch Islet	TK4, TK28	7.514°N, 151.967°E
Republic of Marshall Islands	Majuro	Majuro Atoll Municipality	Mile 28 near Laura, outer reef	M1, M2	7.137°N, 171.038°E
Republic of Marshall Islands	Jaluit	Jaluit Atoll Municipality	Kabbenbock Islet	J5	5.930°N, 169.636°E
Republic of Marshall Islands	Bikar	none (uninhabited)	Within the lagoon	BA1 thru 11	12.223°N, 170.096°E

Light microscopy was done on Nikon 80*i* microscopes with differential interference contrast, imaging with the Nikon DS-Fi1 camera system. Some SEM images were taken with a PhenomWorld G2Pro desktop SEM as in previous work, but many final images, including those using a eucentric (tilting) stage and all those of *Toxarium* and *Climacosphenia*, were made on a Thermo-Fisher Phenom XL SEM (NanoScience Instruments, Phoenix, AZ) after recoating stubs with gold using a Luxor Gold sputter-coater (NanoScience Instruments, Phoenix, AZ). The materials observed were valves or frustules on strewn slides and SEM stubs in the diatom collection of the University of Guam Herbarium (**GUAM**), linked to collection data by the sample number and are referenced here by that sample number. Cataloguing of selected, imaged specimens of individual specimens has begun, using the Specify™ software, but is only in the earliest stages, so that catalog numbers have not yet been assigned to the specimens studied, but the developing website is accessible via the Guam Ecosystems Collaboratorium for Corals and Oceans (**GECCO**) Biorepository Specify Portal at https://specifyportal.uog.edu.

### ﻿DNA isolation, amplification and sequencing

Culture conditions for the strains sequenced and photographed in this study were dependent on where the strains were isolated. Strains isolated from Guam and Saudi Arabia were maintained in a Percival model I-36LL incubation chamber (Percival, Boone, Iowa, USA) at 27 °C under a 12:12 hr light:dark period. Strains isolated from Florida and North Carolina were maintained on a lab bench between 20–24 °C, illuminated by a north-facing window. DNA sequencing of these strains was conducted using the DNeasy Plant Minikit, adding a 45 s incubation in a Beadbeater (Biospec Products, Bartlesville, OK, USA) with 1.0 mm glass pellets for cell disruption. Three markers—the nuclear-encoded ribosomal SSU and chloroplast-encoded rbcL and psbC—were amplified by PCR, purified and sequenced following the protocols outlined in Lobban et al. (2021b).

Sequence data were added to a dataset of diatoms, with *Bolidomonaspacifica* Guillou & Chrétiennot-Dinet, 1999 used as an outgroup (see Suppl. material 1: Table S1 for GenBank accession numbers). Data were initially partitioned by gene, by paired and unpaired sites in SSU secondary structure (determined by the SSUalign program following Lobban et al. 2019) and codon position in rbcL and psbC. Model and partition testing were performed using PartitionFinder 2 with the best model chosen using the corrected Akaike information criterion (AICc). The dataset and partitioning scheme were analyzed under maximum likelihood using RAxML ver. 8.2.7 (Stamatakis 2014) compiled as the pthread-AVX version on an Intel i7 based processor in Linux Mint 18 and IQ-TREE version 1.6.12 for Linux. For RAxML, we ran 15 replicates with 400 rapid BS replicates each with ML optimizations and 4 replicates each with 1500 rapid BS replicates with ML optimization, with bootstrap support assessed by the BS replicates from the run which produced the optimal ML score. Bootstrap support for the IQ-TREE analysis was assessed from 1000 replicates. A Bayesian Inference based phylogeny was inferred by a multi-threaded MPI hybrid variant of ExaBayes version 1.5, using four independent runs with two coupled chains where branch lengths were linked. Convergence parameters monitored included the average deviation of split frequencies of less than or equal to 5% with a minimum of 10,000,000 generations. Bayesian nodal support was assessed using posterior probabilities, with the first 25% of the trees removed as “burn-in”.

### ﻿Morphological analysis

Patterson (1982, 1988) proposed three tests of homology: similarity, conjunction, and congruence. He further proposed that similarity in some aspect of shape, form, or development was sufficient to postulate homology, with congruence as the decisive test, which we take to mean that similarity is the first test. Cracraft (1981) even more strongly argued that similarity was the first step in testing homology, writing that we are compelled to postulate homology for observed similarity. Following that philosophy, we coded multistate characters as additive where such nested similarity was apparent, an approach simultaneously and independently argued by Wilkinson (1992), Lipscomb (1992) and Theriot (1992). Such a treatment does not rely on assumptions about character evolution but instead relies on the principle of parsimony in the same way homology was initially postulated for binary characters (Sober 1983); that is, multistate characters represent nested sets of similarity (and in fact, can be represented as a series of binary characters in a matrix for computer analysis.) The matrix (Suppl. material 2: Table S2) was analyzed under maximum parsimony using Winclada version 1.00.08, which serves as a tool for inputting data into a matrix, as a GUI for inputting commands into NONA version 2 for phylogenetic analysis, and as a tool for tree and character mapping visualization (Nixon 2002), with the following settings: Maximum trees to keep = 1001, Number of replications = 1000, Starting trees per hold = 1, Search strategy = Multiple TBR + TBR, and Search = unconstrained. Two characters were treated as additive: pseudoseptum (0 = absent, 1 = present, 2 = uniform rim all around valve) and valvocopula external poration (0 = absent, 1 = one row, 2 = multiple rows). Settings were heuristic search, 5000 replicates, mult*max*. *Toxariumhennedeyanum* was used as the outgroup.

### ﻿Terminology

The frustules in the taxa treated here appear either heteropolar or isopolar, even though most are attached to the substrate at one pole by mucilage pads or stalks. Heteropolarity may be evident in the shape of the valve (cuneate or clavate), and/or in a cuneate girdle view caused by tapering girdle bands and narrower mantle toward the basal pole. We coded polar morphology as isopolar if the shapes were essentially similar at opposite poles. We avoid the term ‘apices’ because it can be ambiguous, and refer instead to ‘poles’; in heteropolar species we distinguish the ‘basal pole’, i.e., attachment end, from the ‘apical pole’. In some species the valve mantle, especially at the poles, is “recurved as a lip or poorly developed pseudoseptum” (Pickett-Heaps et al. 1991: 723); for this we use the term ‘pseudoseptum’ without qualification (see also Kanjer et al. 2021). In these species, the valvocopula and copula are shaped to fit around the pseudoseptum leaving a groove, the copula with very long fimbriae draped over the valvocopula, as clearly shown in Pickett-Heaps et al. (1991, fig. 18); we call this ‘complex polar architecture’. In some of these species, there is a broad notch in the valvocopula at the poles, producing a tunnel against the pseudoseptum (Round et al. 1990). In yet other species the rim of the valve is straight and the pars interior at the valvocopula pole is correspondingly simpler (fitting into the valve the way the sides of a cookie tin fit into the lid); we call this ‘simple polar architecture’. Most species have three girdle bands in the epicingulum and mature hypocingulum, which, following von Stosch (1975) and Sullivan and Wear (1995), are designated as valvocopula, copula and pleura; the pleura is usually very small and easily hidden or lost in preparation. In *Climacosphenia*, the septum of the valvocopula has a costa along each side with ingrowths forming the craticular bars, i.e., having the structure of a ladder, for which the genus is named. The ingrowths usually meet in the middle and form unions that may be seamless (Round 1982 called those “complete”) or complex; sometimes two outgrowths from one side will anastomose with one from the other side. Other terminology follows Anon (1975) and the revision by Ross et al. (1979).

## ﻿Results

### ﻿Morphology

We review the literature and present our observations on the taxa in the following groups, (1) those with **double walls + pseudosepta**, (2) those with **single walls + pseudosepta**, (3) those with **single walls but without pseudosepta**, (4) *Synedrabacillaris*, (5) *Toxarium*, and (6) *Climacosphenia*.

#### Group 1: Double-walled taxa with pseudosepta

Table 2

##### ﻿ Ardissonea formosa

Taxon classificationPlantaeArdissonealesArdissoneaceae

(Hantzsch) De Toni, 1892

BC4123A3-73E6-5A5C-BA1B-AFB8B61F1479

Fig. 1

###### References.

Peragallo and Peragallo 1897–1908, p. 310, pl. 78, fig. 6; Hustedt 1931–1959, p. 233, fig. 720; Sullivan and Wear 1995, p. 181, figs 1–8; Navarro 1982, p. 260, figs 61–63; Navarro and Lobban 2009, p. 136; Lobban et al. 2012, p. 259, pl. 15, figs 4, 5, pl. 16, figs 1, 2; Park et al. 2018, p. 105, fig. 5.

###### Description from literature.

Hustedt (1931–1959) described this species as linear with ends somewhat narrowed, 200–700 µm long, 15–25 µm wide, 9–10 transapical costae in 10 µm, two rows of areolae between each costa; two or three longitudinal costae and an inner layer, dividing the wall into a series of chambers, each with a single foramen in the inner wall, resulting in three or four chambers. The series of foramina have been described as rows of “pearls,” parallel to the longitudinal lines of the annulus with its underlying costae and sometimes a central costa. Hustedt (1931–1959) specifically referred to a (3^rd^) longitudinal costa down the middle sometimes, and his fig. 720b shows two rows of foramina between the annular lines, suggesting a costa between them, as the chambers were supposed to have only a single opening. A similar situation is seen in Sullivan and Wear (1995: fig. 4). Hustedt (1931–1959) showed two rows of areolae between the transapical costae, but Sullivan and Wear (1995) reported only one and noted the discrepancy. With SEM they showed that the valvocopula had a prominent notch in the advalvar side and a fimbriate edge to the pars interior. They noted that the areolae at the poles were smaller, circular, less dense and more loosely organized but not an apical pore field.

###### Materials examined.

Guam: GU44BH-5!, GU26A!, GU52R-2!. Federated States of Micronesia: Yap: Y39A!, Y42-1!. Marshall Islands: Jaluit Atoll: J5!; Bakir Atoll: BA2!.

###### Observations.

**Figure 1.**
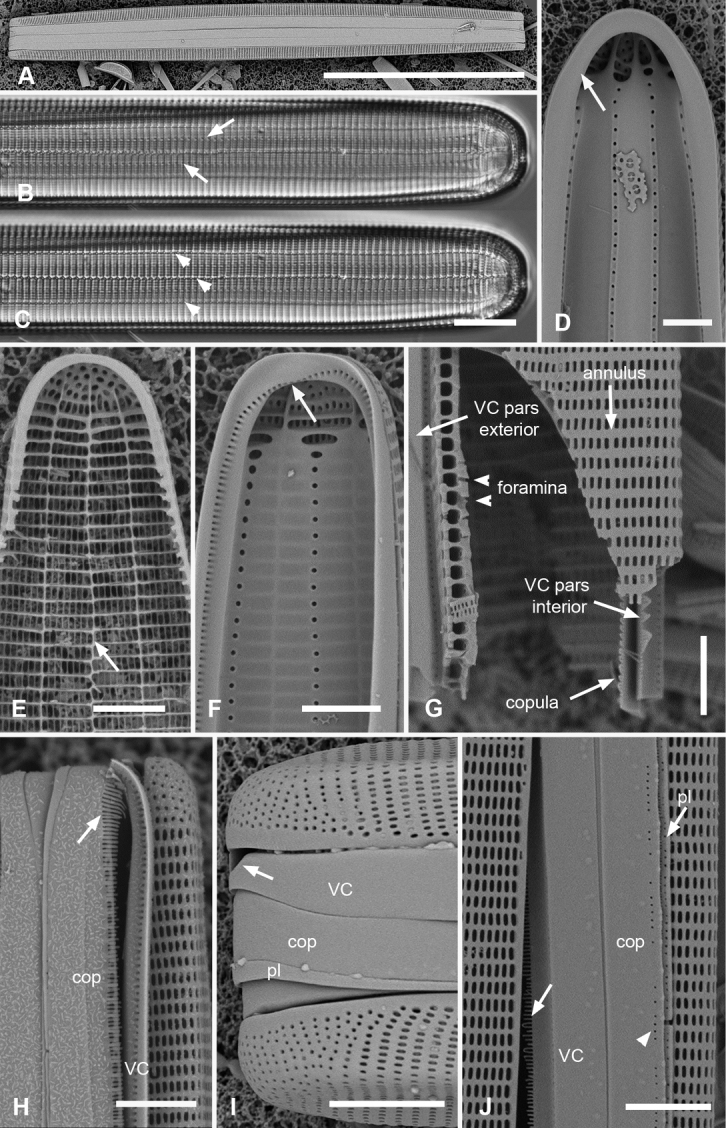
Micrographs of *Ardissoneaformosa***A** whole frustule, length 380 µm, girdle view in SEM, showing isopolarity (Guam: GU44BH-5) **B, C** light micrographs at two focal planes, apical part of valve showing internal foramina (**B** arrows), annulus and central costa (**C** arrowheads) (Jaluit: J5) **D** SEM of internal aspect showing inner wall with foramina and narrow apical pseudoseptum (arrow) (GU44BJ-2) **E** internal aspect of valve pole with inner wall missing, showing a central longitudinal costa (arrow) (Yap: Y42-1) **F** internal view of pole of valve with valvocopula, showing double wall with one central line of foramina, transverse and longitudinal costae showing through the interior wall, and the pores of the valvocopula asymmetrically deflected around the notch (arrow) (Y42-1) **G** broken frustule showing details of wall structure—inner and outer layers separated by costae, inner layer with foramina—in relation to valvocopula and copula (GU44BJ-4) **H** pole of frustule showing long fimbriae at tip of copula (arrow) (GU75A-4) **I** detail of frustule pole in girdle view, showing the three girdle bands with no pores in the external surfaces and the groove in the valvocopula apex (arrow) (GU44BH-5) **J** details of mid portion of frustule in girdle view, showing a line of pores on the edge of the copula (arrowhead) and on the pleura, and the fimbriate inner edge of the valvocopula (arrow) (GU52R-2). Abbreviations used on figures: VC = valvocopula; cop = copula; pl = pleura. Scale bars: 100 µm (**A**); 10 µm (**B–C, I**); 5 µm (**D–H, J**).

Valves linear with broadly rounded poles, 201–300 µm long, 22–24 µm wide, striae 8–10 in 10 µm, isopolar in both valve and girdle view (Fig. 1A). Plastids numerous, lenticular, displaying peristrophy and karyostrophy depending on light intensity (Suppl. material 3: Fig. S1). Three or five longitudinal lines seen in two focal planes (Fig. 1B, C): if five, the annulus, subtending a longitudinal costa, on the valve face (not hidden at the valve margin), a central line where the offset striae meet; between these, on the inner wall, two rows of foramina (Fig. 1B–D). If three lines, one central row of foramina with no longitudinal costa (Fig. 1F). Two additional rows of foramina along the angle of the valve–mantle junction seen in SEM but obscured in LM (Fig. 1D, F). The pattern of costae underlying the inner wall can be traced in Fig. 1D and seen directly in Fig. 1E. We have also seen specimens with one row of foramina along the midline (Fig. 1F). Commonly one or two longitudinal depressions in valve surface, giving a shallow *v* (Fig. 1I) or *w* profile. Striae comprising single row of oval areolae between each transapical costa (Fig. 1C, E, G); loose rows of smaller, circular areolae at the poles, especially noticeable on mantle (Fig. 1I). Narrow pseudoseptum around poles (Fig. 1D, E), elsewhere the double wall forms a thick rim (Fig. 1G). Three girdle bands as follows (Fig. 1F–J). Valvocopula with line of small pores along edge of pars interior, usually obscured in girdle view, deflecting asymmetrically inwards at pole (Fig. 1F), corresponding to location of the notch on advalvar side, which however, is nearly central (Fig. 1I). Interior edge of valvocopula with short fimbriae (Fig. 1G, J). Copula with long fringe of fimbriae at pole, overlapping the valvocopula (Fig. 1H), as described by Sullivan and Wear (1995); an irregular line of pores along abvalvar margin of copula in some specimens (Fig. 1J, arrowhead). Narrow pleura (Fig. 1I, J) with a row of small pores on pars interior.

###### Taxonomic comments.

The biseriate striae described by Hustedt (1931–1959) and the uniseriate striae in Sullivan and Wear’s (1995) materials suggest that they were observing different species. Sullivan and Wear (1995) observed only two bands on the epicingulum and none on the hypocingulum but we observed the pleura. *Ardissoneas.s.* superficially resembles *Synedra* in the current restricted sense (= *Catacombas*) in shape and double wall but differs in lacking rimoportulae and polar pore fields.

##### ﻿ Ardissonea densistriata

Taxon classificationPlantaeArdissonealesArdissoneaceae

Lobban sp. nov.

BE45F726-136F-55E0-BC89-5DA3396ABAE3

Fig. 2

###### Diagnosis.

Differing from *A.formosa* in small, lanceolate valves with 16–17 striae in 10 µm and externally porate copula.

###### Description.

**Figure 2.**
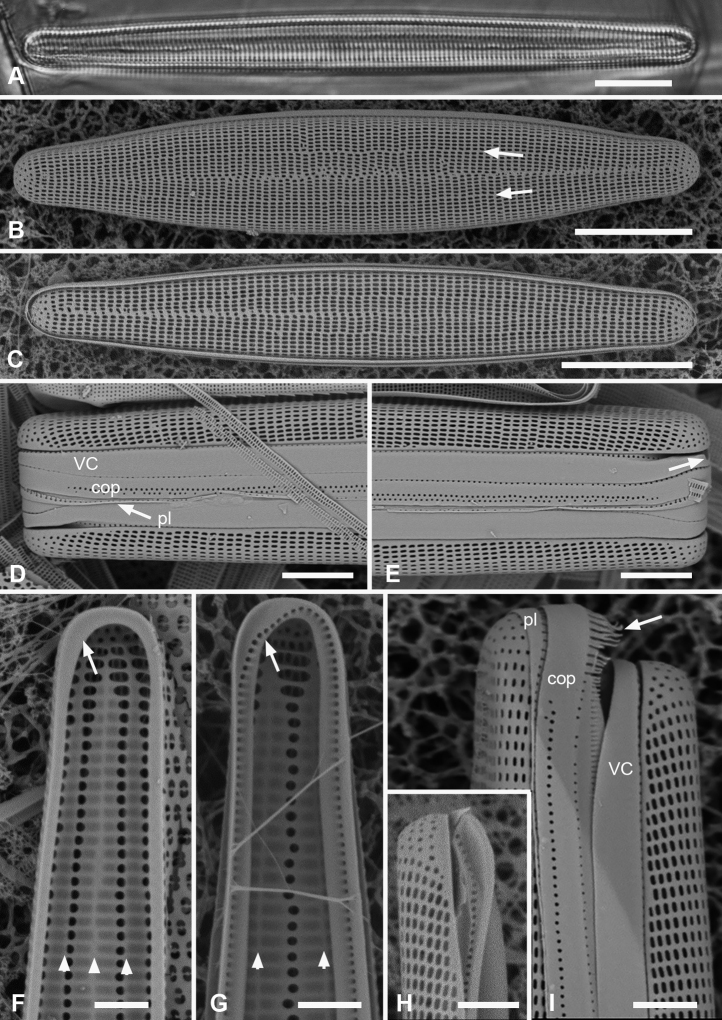
*Ardissoneadensistriata* sp. nov. **A** holotype from Bikar Atoll, BA1 (LM) **B, C** external views of valves in SEM; arrows on **B** point to location of annulus (**B** Y42-1, **C** BA-1) **D, E** external view of two ends of same frustule showing isopolarity; arrow on **E** points to apical notch in VC (J5) **F, G** internal views of valves with one and two middle lines of foramina, arrows showing asymmetrical pseudoseptum on valve in **F** and asymmetrical pole of the valvocopula in **G**; arrowheads point to longitudinal costae (BA1) **H, I** external views of frustules from Bikar and Jaluit, respectively, showing profile of pole of valvocopula in **H** and apical comb of fimbriae on copula in I (arrow). Scale bars: 10 µm (**A–C**); 5 µm (**D, E**); 2.5 µm (**F–I**).

Valves lanceolate, weakly rostrate, 46–103 µm long, 7–10 µm wide, striae parallel, 16–17 in 10 µm (Fig. 2A–C), meeting in an irregular sternum (Fig. 2B, C). Isopolar in both valve and girdle views (Fig. 2A–E). Areolae oval, apically elongate. Two or three longitudinal costae appearing through internal plate (Fig. 2F, G); correspondingly three or four rows of foramina are present. Fig. 2F suggests a costa under sternum. Valve with rim expanded into asymmetric pseudoseptum at poles (Fig. 2F). Girdle bands: Valvocopula pars interior at pole developed asymmetrically, matching the pseudoseptum and with deep notch in advalvar surface (Fig. 2G, arrow, 2H). One line of pores on pars interior, exterior plain (Fig. 2D, I). Copula (Fig. 2D, I) with 1–3 irregular rows of pores on the exterior, pars interior with a row of small pores partially exposed to exterior and a fimbriate inner edge, particularly well developed at poles (Fig. 2I). Pleura narrow with single row of pores, continuous along the cell length (Fig. 2D, E).

###### Holotype

**(designated here).** Specimen at 16.6 mm E and 11.5 mm S of the mark on slide 2911, deposited at ANSP accession # ANSP-GC20089. Fig. 2A.

###### Registration.

Phycobank http://phycobank.org/103235.

###### Type locality.

Marshall Islands: Bikar Atoll, 12.223°N, 170.096°E. Filamentous seaweed sample from shallow subtidal farmer fish territory in the lagoon, precise location not recorded. Collection number BA1, October 2019. Andrew McInnis leg.

###### Additional materials examined.

Federated States of Micronesia: Yap: Y42-1!, Y42-3!.

###### Etymology.

*Densus* + *striatus* (Latin) = densely striated, in comparison to congeners.

###### Taxonomic comments.

Given the small number of double-walled *Ardissonea* species, this species is easily distinguished by the small size, lanceolate shape and relatively high stria density. It is more readily confused in our flora with *Grunowagopacifica* sp. nov. (see below), but in LM the latter can be distinguished by the lack of an inner wall (indicated by absence of foramina) and presence of a longitudinal costa along the midline, in SEM by the extensive poration on the copula. To date this species has not been observed in Guam samples. A different lanceolate, double-walled *Ardissonea* was observed in samples from Chuuk and Jaluit, with stria density 9–12 µm, length 116–211 µm, but we do not have complete information from either population. These samples are similar in shape and stria density to a Red Sea species ‘*Ardissonea AdnU041*’ observed by Sabir et al. (unpublished: http://www.protistcentral.org/Taxa/get/taxa_id/586376 and Ashworth, personal communication). This unnamed species is 83–125 µm long and has no external poration on the girdle bands (as in *A.formosa*). The sequence data have been published as UTKSA0041, shown in our Fig. 33, sister to *A.formosa*.

**Table 2.** Comparison of species of *Ardissonea* sensu stricto.

	Length µm	Width µm	Shape	Stria density in 10 µm	Valvocopula exterior pores	Copula exterior pores	Pleura
*A.robusta*	200–520	30–40	Broadly linear-lanceolate	7	Unknown	Unknown	Unknown
*A.formosa*	200–700	15–25	Linear	9–10	Absent	± irregular abvalvar row	Narrow, continuous, porate
*A.pulcherrima*	240–750	18–28	Linear, the cuneate poles somewhat inflated	9	Unknown	Unknown	Unknown
*A.densistriata* sp. nov.	46–103	7–10	Lanceolate	16–17	Absent	Single abvalvar row	Continuous, porate

#### Group 2: Single-walled taxa with pseudosepta

Table 3

##### ﻿ Ardissonea crystallina

Taxon classificationPlantaeArdissonealesArdissoneaceae

(C. Agardh) Grunow, 1880

C5D5D4CD-3C67-581E-9EE1-CA5FDA9603D2

Fig. 3

###### References.

Peragallo and Peragallo 1897–1908, p. 310, pl. 79, fig. 1; Hustedt 1931–1959, p. 232, fig. 719; Navarro 1982, p. 260, figs 59, 60; Poulin et al. 1986, figs 28–30; Poulin et al. 1987, p. 2689, figs 1–11; Pickett-Heaps et al. 1991, figs 9–19; Lobban et al. 2012, p. 259, pl. 15, figs 1–3; Davidovich et al. 2017, figs 1, 2.

###### Description from literature.

Valves 200–700 µm long, 8–20 µm wide, linear but slightly wider at the middle and poles; 11 striae in 10 µm but much closer in “transitional forms such as var. dalmatica” (Hustedt 1931–1959). Internally with prominent costae developed on every virga except at poles, and with two longitudinal costae corresponding to location of annular ring; spines completely absent. Edge of valve recurved into a pseudoseptum; an interpretive diagram in Pickett-Heaps et al. (1991) shows the relationship between the valve, valvocopula, copula and a small, plain pleura at the pole. Valvocopula and copula have interior comb-like fringe, exterior part with four or more rows of pores arranged decussately; an apical groove between the pseudoseptum and a fold in the valvocopula provides an exit for attachment mucilage (Poulin et al. 1987; Pickett Heaps et al. 1991) but Pickett-Heaps et al. (1991) argued that this groove was a very different structure from the apical pore in *A.formosa* as shown by Round et al. (1990). Shorenko et al. (2016) obtained auxospores of this species and determined a maximum length of 678 µm.

###### Materials examined.

Guam: GU44Y-13!, GU44AV-8!, GU44BJ-4!, GU56A-2!. Federated States of Micronesia: Chuuk: TK4, TK28. Marshall Islands: M1!

###### Observations.

**Figure 3.**
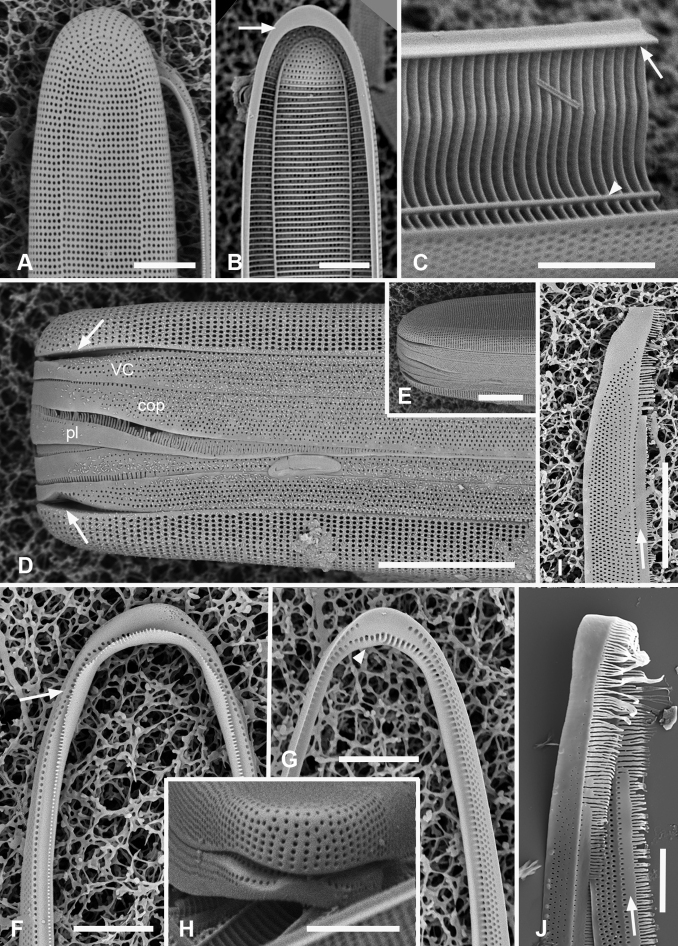
*Ardissoneacrystallina***A, B** external and internal views of valve poles from, respectively Majuro (M1) and Guam (GU44J-4), showing pseudoseptum (**B** arrow) **C** internal SEM view (60° tilt) of broken valve showing internal costae including longitudinal costa subtending the annulus (arrowhead) and continuous rim on valve (arrow) (Chuuk: TK4) **D, E** pole of frustule in girdle view and tilted to 45° to confirm identity; showing the asymmetrical gaps between the pseudoseptum and valvocopula (arrows) (GU75A-4) **F, G** polar portions of valvocopulae from advalvar side and abvalvar sides, respectively, arrow on **F** showing where pores along edge of pars interior move to the back of the gap, arrowhead on **G** showing that slits do not form an open comb (Chuuk: TK4) **H** polar view of pole with valvocopula showing the asymmetrical gaps (TK4) **I, J** poles of copulae from wild material (TK4) and culture (GU44AV-8), respectively, arrows indicating fused bases of fimbriae away from the pole. Scale bars: 10 µm (**D, E, I**); 5 µm (**A–C, F–H, J**).

Specimens from Guam and Chuuk 220–350 µm long, 11–16 µm wide, isopolar in both valve and girdle views, striae 16–19 in 10 µm, slightly offset on internal and external sides of annulus (Fig. 3A, B). Annulus well separated from valve-margin junction and clearly visible in LM and SEM, including around the poles (Fig. 3A, B). Pseudoseptum at poles (Fig. 3B) and rim along entire valve (Fig. 3C), facing pars interior of the valvocopula. Apical spines not observed (Fig. 3A, D, H). Costae well developed (Fig. 3B, C). Three girdle bands present (Fig. 3D): valvocopula fimbriate (Fig. 3D, F–H), forming a slot on one side of the pole and a sharper notch on the other (Fig. 3D arrows, Fig. 3H); pores on pars interior passing across back of flange, elongated into slits at pole, clearly closed at inner margin (Fig. 3G arrow) and pars interior expanded and recurved at pole (Fig. 3F). Pores on pars exterior discontinuous around pole in front of the groove (Fig. 3F–H). Copula (Fig. 3D, I, J) broader and flat, even at pole, with up to 10 rows of circular pores in a decussate pattern; pars interior fimbriate with free fimbriae highly developed at pole, where they overlap the valvocopula, elsewhere shorter fimbriae arising from fused base (arrows, Fig. 3I, J). Small difference in shape, number of rows of pores and length of fimbriae between Fig. 3I and J may be due to origin of material (Fig. 3D and 3I are from Chuuk wild samples, Fig. 3J from Guam culture) but copulae from wild material from Guam (not shown) also had >6 rows of pores. Pleura (Fig. 3D) fimbriate, an apical cap extending along the valve as a very narrow ribbon.

###### Taxonomic comments.

*Ardissoneacrystallina* cannot remain in *Ardissonea* sensu stricto and will be transferred in the Taxonomic Revisions section at the end of the paper. Álvarez-Blanco and Blanco (2014) show Mediterranean specimens of *Ardissoneadalmatica* (Kützing) De Toni, restored to species status without comment, but the length ranges only up to 151 µm. There was a row of papillae/spines around the pole, not present in our material and the length range in the literature is 100–151 µm, but the stria density matches our specimens more closely. We leave our identification at *A.crystallina* pending broader study. Although Hustedt (1931–1959) noted *A.crystallina* as distributed on coasts of warmer waters, Poulin et al. (1987) reported it as the only *Ardissonea* on the coast of Québec, an area that is far from tropical. Likewise, Shorenko et al. (2016) and Davidovich et al. (2017) collected it from the Black Sea. We do not agree with Pickett-Heaps et al. (1991) that there is a fundamental difference in the architecture at the pole of the valvocopulae of *A.formosa* and *A.crystallina* as both involve irregularities in the surface of the flange of the valvocopula where it lies below the pseudoseptum.

**Table 3.** Comparison of species of *Synedrosphenia*†.

	**Length µm**	**Greatest width µm**	**Shape**	**Stria density in 10 µm**	**Valvocopula exterior pores**	**Copula exterior pores**	**Pleura**
*S.baculus*	200–1000	13–27	Linear	10.5	Unknown	Unknown	Unknown
*S.bikarensis* sp. nov.	214–277	12–14	Spathulate	21	3 rows reducing to 1; very coarse fimbriae	Irregular lines of slits (rimae)	Separate porate polar caps, apical larger
*S.crystallina*	200–700	8–20	Linear	11	2–3 rows	Ca. 5 rows	Probably continuous
	[220–350]	[11–16]		[16–19]	[3–4 rows]	[10 rows]	
*S.giennensis*	195	35	Clavate, widest ¼ from apical pole	12	Unknown	Unknown	Unknown
*S.gomphonema*	250–475	27–36	Clavate, subrostrate apical pole	12 basal / 14 apical	4 rows decreasing to 2 near basal pole	Decussate striae, forming fimbriae on abvalvar margin	Apical cap
				[13–14/14–16] †			
*S.fulgens*‡	330–346	9.5–11.5	Linear, center slightly inflated	15–16	Numerous rows	Unknown	Unknown
*S.licmophoropsis* sp. nov.	600–735	27	Cuneate	19	5 rows reducing to 2	10 rows decreasing to 5	Narrow, continuous
*S.parva* sp. nov.	196–250	18	Clavate	16 basal, 18–20 apical	3 rows decreasing to 1	7 rows decreasing to 3	Polar caps porate; apical cap large, fimbriate
*S.recta* sp. nov.	406–430	13–16	Linear	16–18	None	Slits	Small polar caps joined by narrow perforated band, not fimbriate?

† Data for known species are from literature cited in text, data for Micronesian specimens given in brackets where different. ‡ data for type material from Kanjer et al. (2021).

##### ﻿ Synedrosphenia gomphonema

Taxon classificationPlantaeArdissonealesArdissoneaceae

(Janisch & Rabenhorst) Hustedt, 1932 in Hustedt, 1931–1959

B17DD449-77D5-5434-8FAA-86E64856B68E

Figs 4 , 5

###### References.

Janisch and Rabenhorst 1863, p. 13, pl. 2, fig. 6; Grunow 1877, p. 169, pl. 194, fig. 3a, b, c, d ; Peragallo and Peragallo 1897–1908, p. 313, pl. 78, figs 1, 2 (as *S.cuneata* Grunow); Hustedt 1914 in Schmidt et al. (1874–1959), pl. 305, figs 32, 33; Hustedt 1931–1959, p. 231, fig. 723; Cheng and Gao 2011, figs 147, 148; Hein et al. 2008, pl. 14, fig. 3, pl. 15, fig. 1; Hernández-Almeida et al. 2013, figs 39–42 (as *Synedrospheniacuneata*).

###### Description from literature.

The valve shape as shown by both Grunow (1877) and Hustedt (1914, 1931–1959) has a basal part with nearly parallel sides that then tapers wider, flaring more abruptly near the apical pole. Hustedt gave length as 250–475 µm, maximum width 27–36 µm, basal and apical stria densities 12 and 14 in 10 µm, respectively. Striae change abruptly from parallel to radiate where the apical pole narrows. Transapical costae on the virgae often continuous across the valve and obscuring the midline; a longitudinal rib near each margin. Round et al. (1990: 444) noted that in *Synedrosphenia* stria density is higher on the girdle bands than on the valve (Fig. 10A), whereas in *Climacosphenia* it is lower on the girdle bands (Fig. 28A). This holds true for all our species.

###### Materials examined.

Guam GU44Y-13!, GU44BD-4!, GU44BH-5!, GU 44BJ-2!, GU44BJ-4!. Federated States of Micronesia: Chuuk: TK28!; Yap: Y42-1!. Marshall Islands: Jaluit, J5!.

###### Observations.

**Figure 4.**
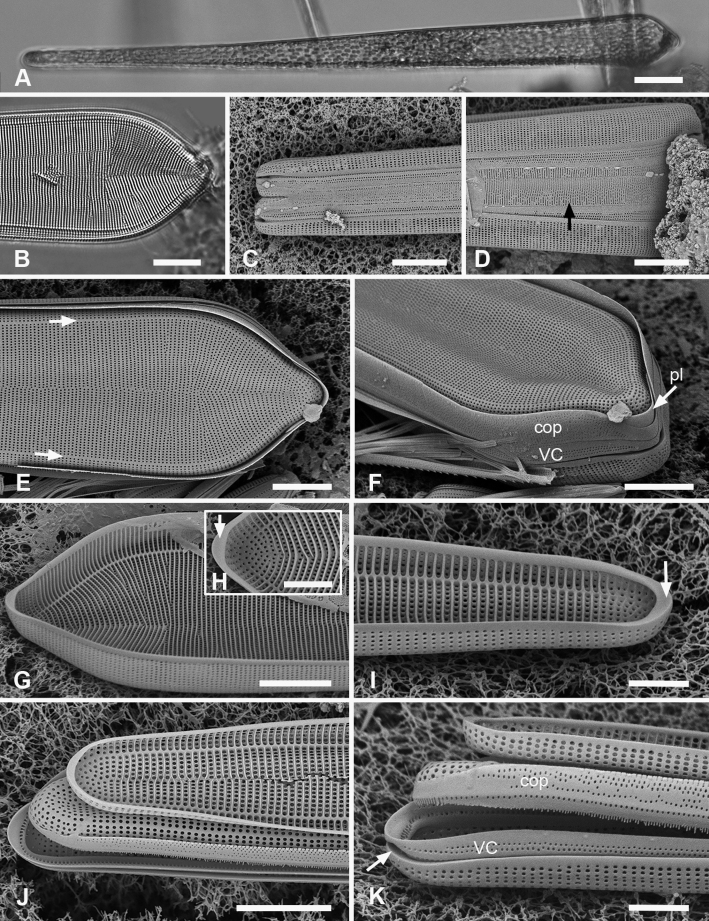
*Synedrospheniagomphonema***A** live specimen in valve view showing heteropolar cell shape and plastids (GU44BJ-4) **B** apical portion of cleaned valve in LM showing change in stria orientation where cell narrows (GU44BD-4) **C, D** recently divided frustule in girdle view with details of basal and apical poles, respectively, showing heteropolarity; black arrow indicates slit formation on the copula (GU44BJ-4) **E, F** valvar view of frustule at 0° and 60° tilt, respectively, arrows on **E** showing location of annulus, labels on **F** naming the girdle bands (abbreviations as before) **G–I** apical and basal portions of valve, internal view tilted 60°, showing pseudosepta at poles (**H, I** arrows) and transverse and longitudinal costae (TK4); inset (**H**) shows tip of same valve at 0° tilt with lack of costae between the last few striae but continuation of annulus **J, K** basal portions of a broken, recently divided frustule from Chuuk, viewed at 0° and 60° tilt, respectively, valves and girdle bands of one daughter cell with one valve of other daughter on top; arrow shows the notch between valve and valvocopula (TK4). Scale bars: 25 µm (**A**); 10 µm (**B–G, I**); 5 µm (**H, J**).

**Figure 5.**
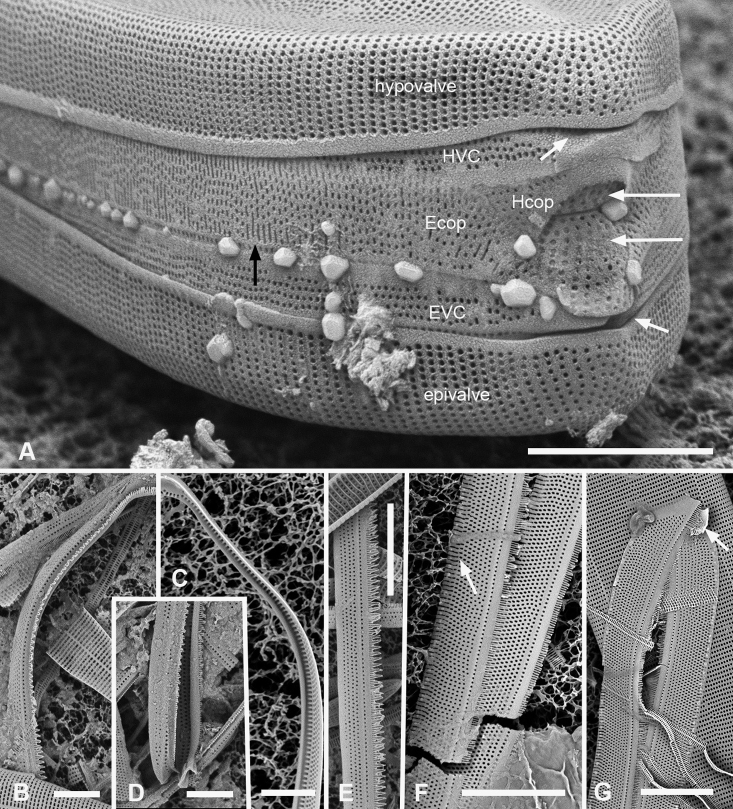
*Synedrospheniagomphonema* cont. **A** apical pole of frustule in polar view tilted 60°, showing the valvocopula and copula of the hypocingulum (HVC and Hcop) largely hidden under the epicingulum bands (EVC and Ecop); the pleurae are missing and in the space where they were the tips of the newly formed valves are visible (long arrows). Short white arrows point to the apical notches slightly off-center in opposite directions; black arrow shows some slit formation in the copula (GU44BJ-4) **B–E** valvocopula with apical pole in advalvar view (**B**) showing recurved pars interior at the tip, abvalvar view (**C**), basal pole (**D**) and middle (**E**), all showing fimbriae (**B, D, E** from same specimen in GU44BI-4, **C** from TK4) **F, G** copula middle and apical pole, respectively, external view, showing fimbriae developing on the abvalvar edge of the pars exterior (arrow) (TK4). Scale bars: 10 µm (**A, E, F, G**); 5 µm (**B–D**).

Frustules heteropolar, cuneate in both valve and girdle view, numerous small, oblong plastids (Fig. 4A–D). Valve margins parallel near basal pole, then gradually widening with slight flare below subrostrate apical pole (Fig. 4E, F). Length 255– >414 µm, greatest width 27–29 µm, stria densities in 10 µm 13–14 basal, 14–16 apical. Striae parallel except changing abruptly to radial at apical pole with several interpolated short striae (Fig. 4B, E, G); areolae circular. Annulus near valve–mantle junction but clearly visible in both LM and SEM (Fig. 4B, E–I), continuous around the poles (Fig. 4E, H, J). Internal costae underlie annulus and virgae, except at the poles. Three girdle bands present (Figs 4F, 5A). Valvocopula (Figs 4J, K, 5B–E) with about 4 longitudinal rows of pores on pars exterior, decreasing to two near basal pole, pars interior with a fimbriate margin comprising a shelf with a single row of pores and a groove; poles with asymmetrical notch (Figs 4C, K, 5A). Copula (Figs 4J, K, 5A, F, G) pars interior with longitudinal line of pores along edge, the rest of pars interior plain with fringe of fimbriae, longer around apical and basal poles (Figs 4K, 5G). Copula pars exterior abruptly narrowed at apical pole, pores arranged in ± decussate striae across the band reaching abvalvar margin and forming fimbriae; no pores around poles. Copulae in Guam specimens tending to form slits (Fig. 4D and Fig. 5A black arrows), not observed in Chuuk specimens (Fig. 5F, G). Pleura visible as apical cap (Fig. 4F), seemingly lost during cell division (Fig. 5A; compare Fig. 12E, F).

**Figure 6.**
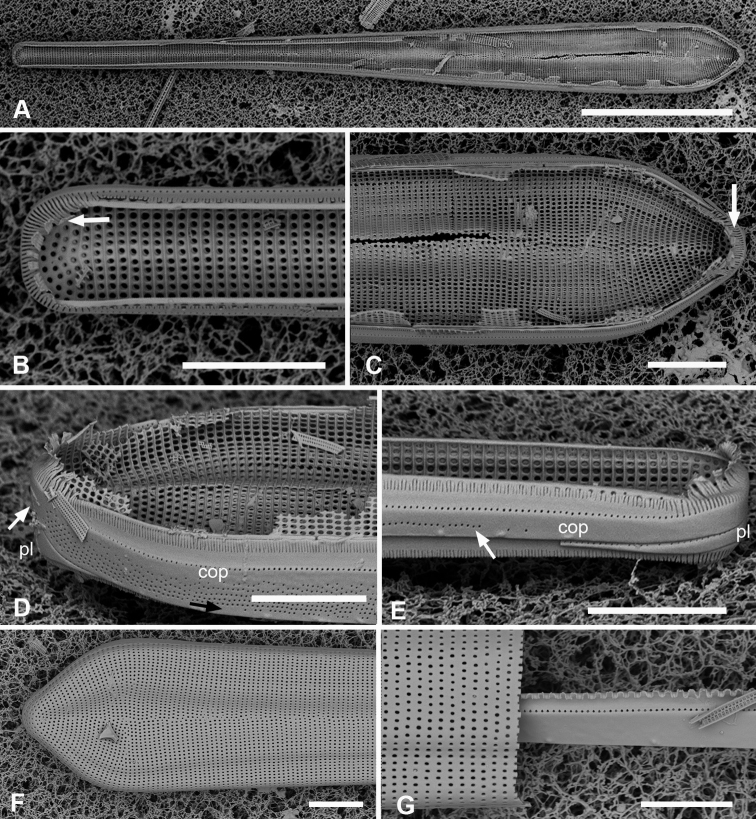
Specimens of *Sceptroneiscuneata* from Grunow’s Honduras sample **A–C** whole frustule at 0° tilt and details of basal pole and apical poles, respectively, showing internal costae and the copula; arrows point to long fimbriae overlapping valvocopula at both poles **D, E** same specimen, tilt = 60°, details of apical and basal poles, respectively, showing copula, including shallow cut-away matching smaller pleura (arrow on **D**), hyaline abvalvar margin of copula (black arrow on **D**), and reduced poration near basal pole (arrow on **E**) **F** apical part of valve in external view **G** valvocopula associated with same specimen. Scale bars: 50 µm (**A**); 10 µm (**B–F**); 5 µm (**G**).

A frustule of *Sceptroneiscuneata* in remnants of Grunow 639 (Fig. 6), showed the valve and copula. Valve stria densities were lower than in our material: basal 12, apical 13 in 10 µm. The copula differed in having a hyaline abvalvar border with no tendency to form fimbriae (Fig. 6D, E) and a longer hyaline area at the basal pole (Fig. 6E vs Fig. 4F). The apical cap of the pleura may be smaller than in our specimens. A valve apical pole with part of the valvocopula (Fig. 6F, G) had 11 striae in 10 µm and there were no pores in the exterior, in contrast to Guam specimens.

###### Taxonomic comments.

There are too many differences from Grunow’s specimens to identify our species as *Sceptroneiscuneata*. We also cannot confirm whether our specimens match *Synedrospheniagomphonema* without SEM of authentic specimens. The taxonomy of *Synedrosphenia* is convoluted. As explained by Fourtanier and Kociolek (1999):

“*Originally described (and validly published) as a subgenus of*Synedra: Synedra*subgenus*Synedrosphenia*H. Peragallo in H. and M. Peragallo 1900 [Peragallo and Peragallo 1897–1908], p. 308, 312. Three species were included*, Sceptroneis [Synedra] cuneata*Grun.*, S.clavata*Grev. and*S.dubia*Grun. and no type was designated. Although Azpeitia 1911, p. 220 introduces the name as “El genero ó subgenero*Synedrosphenia” *(the genus or subgenus*Synedrosphenia) , *he treats it as a genus when describing his new species*Synedrospheniagiennensis. *Although*S.giennensis*Azpeitia is the only species formed as binomial with*Synedrosphenia*in Azpeitia 1911, Azpeitia, in his treatment of*Synedrosphenia*also clearly included the 3 species that constituted Peragallo’s subgenus (but did not make transfers to genus*Synedrosphenia) . *No type was designated by Azpeitia. The generic name cannot be attributed directly to Azpeitia because he did not provide a generic description. The authorship of*Synedrosphenia*is therefore “(H. Peragallo in H. Perag. and M. Perag.) Azpeitia,” and the type has to be chosen among the species originally included in the subgenus by Peragallo. The choice of*S.giennensis*as the type species by Round, Crawford and Mann (1990) p. 444 and 704 is therefore untenable.*”

Hustedt (1931–1959) very clearly synonymized *Sceptroneiscuneata* into *Synedrospheniagomphonema* (1931–1959, fig. 723, pp. 220–221 in Jensen’s (1985) translation, pp. 241–242 in orig.). The drawing in Janisch and Rabenhorst (1863) is, as Hustedt (1931–1959) said, “very schematic and erroneous to be sure,” showing a prominent sternum, but Hustedt based his synonymy on examination of many specimens from both Janisch and Grunow, both of whom worked on collections from Honduras. The other two species, *S.clavata* and *S.dubia*, are poorly known, perhaps not seen since their original descriptions, both characterized as very rare, and perhaps not even attributable to this genus. We thus propose *Sceptroneiscuneata* Grunow as the lectotype, below, recognizing the synonymy with *S.gomphonema* as the current name for the Honduras species, but pending evidence from Janisch and Rabehnorst’s materials to test whether our species is the same. *Synedrospheniagiennensis*Azpeitia (1911, p. 219, pl. 6, figs 1, 2) was based on LM of a single clavate valve, maximum width ¼ of the way from one end, costae between striae not always continuous across the valve. Azpeitia made no mention of longitudinal costae. The dimensions were 195 × 35 µm, 12 striae in 10 µm, areolae 19–20 in 10 µm. This does appear to belong in *Synedrosphenia* but certainly needs further study.

##### ﻿ Synedrosphenia bikarensis

Taxon classificationPlantaeArdissonealesArdissoneaceae

Lobban sp. nov.

A8655576-6D79-5DB1-8887-5CF9CFA4FF60

Figs 7 , 8

###### Diagnosis.

Valve spathulate, heteropolar, differing from *S.parva* and *S.gomphonema* in valve shape and in the fimbriate pars exterior of the copula.

###### Description.

**Figure 7.**
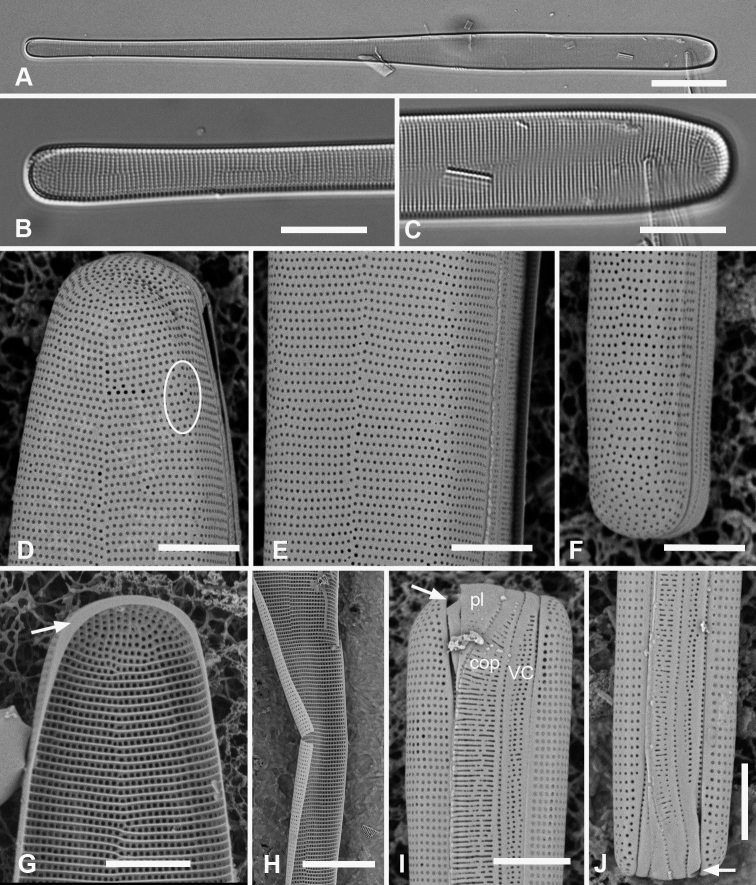
*Synedrospheniabikarensis* sp. nov. (all from Bikar sample BA5) **A–C** holotype whole valve, basal and apical portions, respectively (LM) **D–F** external SEM views of apical, middle and basal portions, respectively, of a frustule, showing valve structure. Oval in **D** highlights area where break in striae across the annulus can be seen, even though the annulus is not readily visible **G, H** internal views of apical pole with pseudoseptum (arrow) and middle portion (**H**) showing transverse costae and the lack of a longitudinal costa under the annulus **I, J** frustule in girdle view, apical and basal portions, respectively, showing heteropolarity and girdle bands of the epicingulum. Arrows mark the notches visible on the hypovalvocopula at the apical pole and epivalvocopula at the basal pole, indicating the asymmetry. Scale bars: 25 µm (**A**); 10 µm (**B, C, H**); 5 µm (**D–G, I, J**).

**Figure 8.**
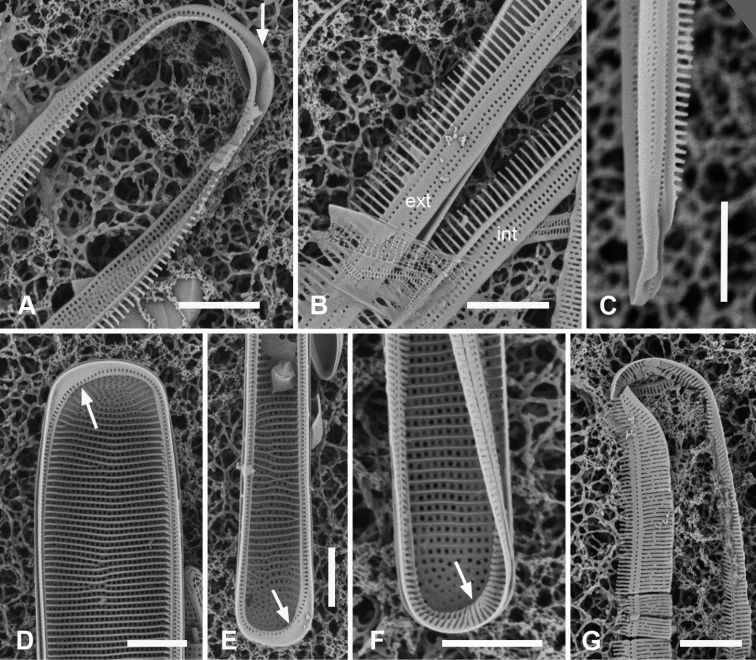
*Synedrospheniabikarensis*, cont. **A–C** valvocopula: apical portion in advalvar view (**A**) with asymmetrical notch at apical pole (arrow), middle portion (**B**) with long sturdy fimbriae (ext = external surface, int = internal surface), and basal portion in girdle view (**C**) **D, E** internal views of apical and basal poles, respectively, of same valve with valvocopula, showing opposite asymmetry of the notches (arrows) **F** basal portion of valve with attached girdle bands, showing how the copula fimbriae fit over the valvocopula at poles (arrow) **G** apical portion of copula showing poration. Scale bars: 5 µm.

Cells heteropolar, probably attached by basal pole to seaweeds. Valves spathulate, 214–277 µm long with linear, wider apical portion 12–14 µm wide, narrowing abruptly about halfway down the valve and then more gradually to 6 µm near the basal pole, which is weakly inflated to 7 µm (Fig. 7A–C). Both poles bluntly rounded. Striae parallel except weakly radiating at apical pole, 21 in 10 µm; areolae circular, 25 in 10 µm (Fig. 7D–F). Internally costae on all virgae except at poles (Figs 7G, 8D, E). Bifacial annulus indicated only by an offset between striae on valve face and mantle (Fig. 7D, oval). There was a junction down the midline of the valve where the inward-growing striae met but no sternum (Fig. 7B–E).

Girdle bands: Cingulum comprising two large, closed bands and a large apical cap (pleura) (Figs 7I, J, 8). Valvocopula porate (Figs 7I, J, 8A–E); the pars interior forming a sturdy comb (Fig. 8A–C) that matched up with the internal costae of the valve (Fig. 8D), though it was not clear whether they aligned or interdigitated with the costae. This was followed by an internal ridge and an adjacent series of pores, often hidden under the valve (Figs 7I, J, 8B, C). The pars interior was modified asymmetrically at each pole (Fig. 8D, E), with a shelf supporting a prominent asymmetrical notch (Fig. 7I, J and Fig. 8A, C). Apical and basal asymmetries were on opposite sides of the valvocopula (Fig. 8D, E, arrows) and the symmetry on the hypotheca was the mirror image of the epitheca (Fig. 7I, J, arrows). Pars exterior with three irregular rows of pores, 30 in 10 µm, reducing to two and then one at the basal pole. The copula also had a comb along inner edge of pars interior and a row of pores just under the overlapping valvocopula (Fig. 8F, G). Copula internal comb continued around the apical pole; pars exterior dominated by a series of long slits (rimae) that often extended to abvalvar margin (Figs 7I, J, 8G), resulting in a fringe along the edge of the cingulum except at poles. Both valvocopula and copula narrowed at the poles, leaving a space filled by the pleura, which is possibly a continuous band (Fig. 7I, J). We did not see the pars interior of the pleura but it is likely to be fimbriate (see *S.parva* below).

###### Holotype

**(designated here).** Specimen at 14.9 mm E and 9.6 mm S of the mark on slide 2920, deposited at ANSP, accession # ANSP-GC20090. Fig. 7A–C.

###### Registration.

Phycobank http://phycobank.org/103236.

###### Type locality.

Bikar Atoll, Marshall Islands, 12.223°N, 170.096°E, on seaweed filaments attached to coral from a farmer fish territory in the lagoon. Collection number BA-5, October 2019. Andrew McInnis leg.

###### Etymology.

Named for Bikar Atoll, Republic of the Marshall Islands, where it was collected.

###### Taxonomic comments.

The apparent absence of a bifacial annulus is unique among the *Synedrosphenia* species described here, where there is usually at least a hyaline line visible on the valve face or at the face-mantle margin. There is, however, a misalignment of striae evident in places along the valve–mantle junction (Fig. 7D, E), as observed in the other bifacial-annulus taxa described here, and hence no reason to suppose the growth of the valve proceeds differently. We hypothesize that there is an effective annulus present.

##### ﻿ Synedrosphenia licmophoropsis

Taxon classificationPlantaeArdissonealesArdissoneaceae

Lobban sp. nov.

83F11755-EDF7-550E-BF74-C6A871F74A9C

Figs 9 , 10

###### Diagnosis.

Extremely long heteropolar cells, differing from other species in the cuneate shape in valve and girdle views.

###### Description.

**Figure 9.**
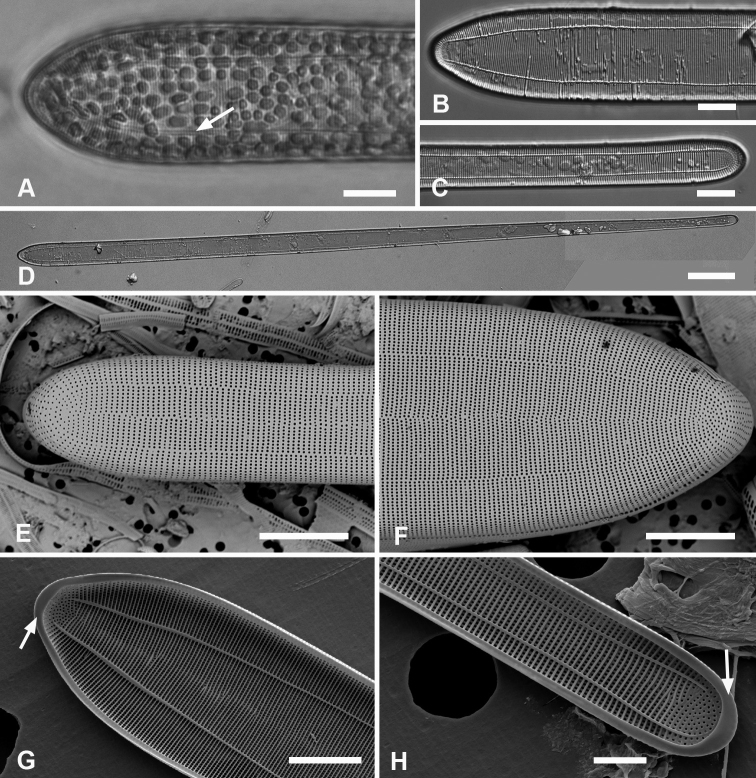
*Synedrosphenialicmophoropsis* sp. nov. **A** apical pole of living cell showing plastids and annulus (arrow) (GU44AV-8) **B–D** holotype valve from GU55B-4 in LM: apical and basal portions showing annulus and entire valve **E, F** external view of valve, SEM: basal and apical poles, respectively, same scale (GU44Y-13) **G, H** apical and basal poles, respectively, valve in internal view, SEM, showing longitudinal and transverse costae and pseudosepta (arrows) (GU56A-2). Scale bars: 50 µm (**D**); 10 µm (**A–C, E–G**); 5 µm (**H**).

**Figure 10.**
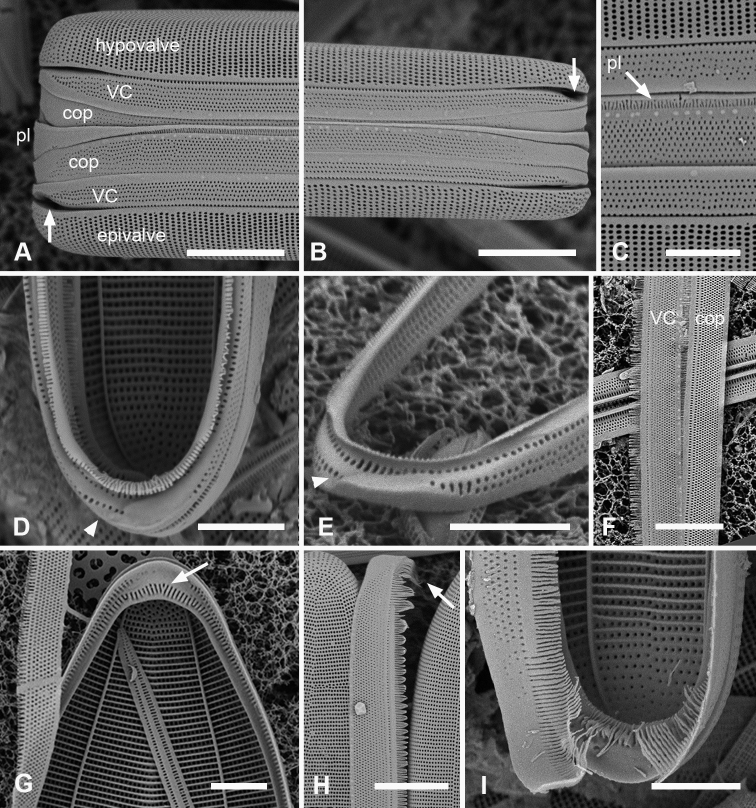
*Synedrosphenialicmophoropsis* sp. nov., girdle bands **A, B** frustule in girdle view with thecal elements labeled, respectively on the apical part and the basal pole of the same cell also showing the asymmetrical apical notches in the valvocopula (arrows) (GU55D-3) **C** small portion near middle of frustule where pleura (pl) is exposed (GU76B-2) **D, E** basal pole of valvocopulae at 0° tilt with opposite valve in the background and tilted to show complex shape of the pars interior, arrowhead pointing toward notch (GU76B-2 and TK4, respectively) **F** portions of valvocopula and copula showing fimbriate inner edges (GU55D-3) **G** valvocopula, apical pole in abvalvar aspect with attached valve, showing elongated pores on inner edge (arrow) (GU44BJ-2) **H** apical portion of copula showing very long fimbriae at the tip (arrow) (GU55D-3) **I** relationship between first two girdle bands at basal pole, the long fimbriae of the copula overlapping the valvocopula (GU76B-2). Scale bars: 10 µm (**A, B, F, H**); 5 µm (**C–E, G, I**).

Frustules heteropolar, tapering uniformly in both valve and girdle view (Figs 9D, 10A, B), attached to seaweed substrata by mucilage stalks; plastids numerous, lenticular to oval (Fig. 9A, B). Valve margins parallel near basal pole, 14 µm wide, widening evenly to 27 µm wide just before the cuneate obtuse or slightly rostrate apical pole, length 600–735 µm (Fig. 9A–D). Striae parallel except radiate near apical pole, 19 in 10 µm near the apical pole, 16–17 near the basal pole (Fig. 9E–H). Annulus, evident even in living specimens (Fig. 9A), nearly halfway between centerline and valve margin (Fig. 9B–H), underlain by a longitudinal costa except at apical pole (Fig. 9G, H). Valve border with a rim along its length and shallow pseudosepta at each pole (Fig. 9G, H), the latter matched by extensive development of the valvocopula. In girdle view, three bands evident, all fimbriate (Fig. 10A–C, F). Valvocopula bearing a row of pores at the edge of the pars interior and five rows of pores on the exterior near the apical pole, reduced to two near the basal pole, the pores in a decussate pattern, 27 in 10 µm, the edge of the pars interior bearing short fimbriae near the apical pole, longer elsewhere (Fig. 10D–F). The pars interior of the valvocopula at the poles formed a deep shelf with an irregular surface (Fig. 10D, E), creating a notch on one side and a shallower, longer gap on the other (Fig. 10A, B); notches were on opposite sides of both the valvocopula itself and when comparing epitheca and hypotheca (Fig. 10A, B arrows). The line of pores along the edge of pars exterior moved to inner edge of the gap and became slitlike (Fig. 10G, arrow). Copula (Fig. 10A, B, F, H) wider, again with pores in a decussate pattern, about 10 rows near apical pole diminishing to five at the basal pole, a wide delicate fringe along the edge of the pars interior. The relationship between the first two girdle bands at the poles is shown in Fig. 10I. The pleura (Fig. 10A–C) had a broad apical cap, quickly tapering to a narrow external line with fimbriate pars interior, broadening again somewhat at the basal pole; this band lacked pores.

###### Holotype

**(designated here).** Specimen at 13.0 mm E and 5.0 mm S of the mark on slide 455, deposited at ANSP accession # ANSP-GC20086. Fig. 9B–D.

###### Registration.

Phycobank http://phycobank.org/103237.

###### Type locality.

“Pete’s Reef” (commercial dive site), Agat Bay, 13.347°N, 144.639°E. Epiphytic on *Halimeda* at 10 m, collection number GU55B-4, 13 Dec. 2008. C. Lobban and M. Schefter, leg.

###### Additional materials examined.

Guam: GU44Y-13!, GU44AV-8!, GU54A-B!, GU56A-2!, GU66B-6!, GU76B-2. Federated States of Micronesia: Yap: Y-37-8!; Marshall Islands: Bikar Atoll BA-5!.

###### Etymology.

Named for its resemblance to certain large *Licmophora* spp., especially *L.attenuata* Lobban, Tharngan and Ashworth.

###### Taxonomic comments.

Longer and more evenly tapered, apical pole broadly rounded, striae denser compared to *S.gomphonema*; valves appearing narrower than *S.gomphonema* because of the greater length, but the apical poles are equally wide.

##### ﻿ Synedrosphenia parva

Taxon classificationPlantaeArdissonealesArdissoneaceae

Lobban sp. nov.

B483AA28-E6A7-50B6-AE64-0877CF7E49E5

Figs 11 , 12

###### Diagnosis.

Shorter and narrower than *S.gomphonema* and more finely striated. Girdle bands distinctive: copula with very long internal fimbriae at poles, pleura a large apical cap.

###### Description.

**Figure 11.**
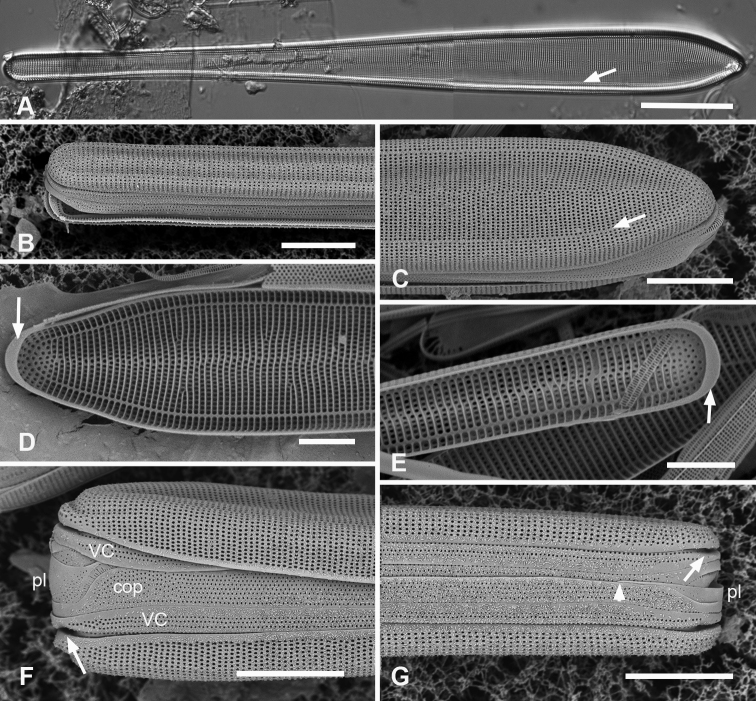
*Synedrospheniaparva* sp. nov. **A** holotype from Guam in LM, arrow indicates annulus near valve face–mantle junction (GU75A-4) **B, C** valve in external view (SEM), basal and apical poles, respectively, arrow indicates annulus (GU75A-4) **D, E** internal views of apical and basal poles, respectively, arrows indicate pseudoseptum (J5) **F, G** frustule in girdle view, apical and basal portions, respectively, showing the girdle bands including the large apical cap of the pleura, smaller at the basal pole and perhaps not connected (arrowhead in **G** indicates the apparent end of the pleura); arrows on **F, G** point to polar notches (GU75A-4). Scale bars: 25 µm (**A**); 10 µm (**B, C, F, G**); 5 µm (**D, E**).

**Figure 12.**
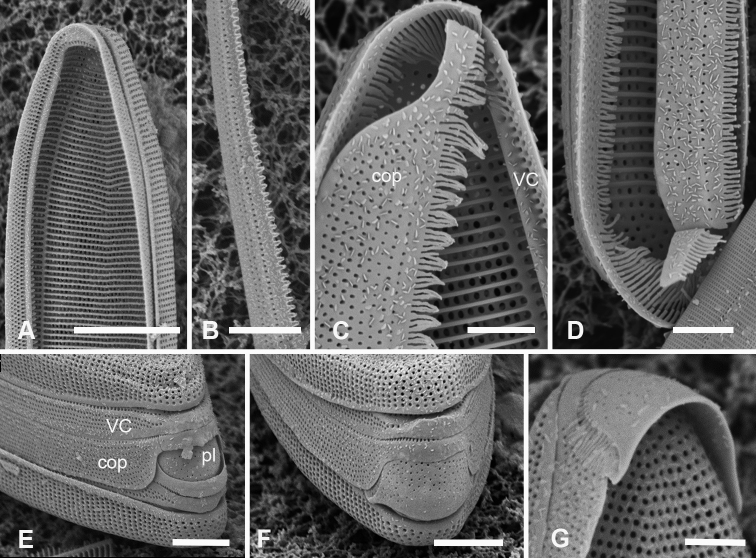
*Synedrospheniaparva* sp. nov., cont. All GU75A-4 **A, B** valvocopula showing apical internal side from abvalvar position, and portion of middle from advalvar side, respectively, showing row of pores along base of pars interior and fimbriae **C** internal view of apical pole with valvocopula in situ and the copula, broken in the middle, covering the valvocopula around the apical pole and left side **D** basal pole of same specimen, again showing the long fimbriae overlapping the valvocopula **E, F** frustules tilted to show polar (apical) architecture of, respectively a recently divided cell, where the pleura is in place between the copulae of the two cingula, and a non-dividing cell **G** detail of a pleura loosened from its position and showing some of the fimbriae (still tucked under the copula at top of image). Scale bars: 10 µm (**A**); 5 µm (**B, D–F**); 2.5 µm (**C, D, G**).

Frustules heteropolar in valve and girdle views (Fig. 11A, F, G) with numerous small plastids (not shown). Valves 196–250 µm long, 18 µm wide near the apical pole, clavate with acutely rounded to weakly rostrate apical poles (Fig. 11A–E). The annulus evident in LM but close to edge of valve face (Fig. 11A, C). Valve surface with weak longitudinal furrows, mantle deeper toward apical pole (ca. 11 vs. 5 areolae deep) (Fig. 11 B, C, F, G). Striae 16 in 10 µm near basal pole, 18–20 in 10 µm near apical pole, parallel except radiate at apical pole (Fig. 11A–E). Pseudosepta present at both poles (Fig. 11D, E); longitudinal costae subtending annulus and transverse costae on vimenes except at apical pole (Fig. 11D, E). Three girdle bands are present (Figs 11F, G, 12E–G). Valvocopula with a row of pores along outer edge of pars interior plus three rows decreasing to one on pars exterior; pars interior with a notched flange at each pole and a fimbriate edge (Fig. 12B). Copula with up to nine rows of pores at apical pole in decussate pattern, decreasing to three near basal pole (Figs 11F, G, 12C, D). Pleurae a pair of polar caps (connecting band not seen), the apical cap relatively massive with small pores and fimbriate (Figs 11F, 12E–G), basal cap smaller, with fewer pores (Fig. 11G arrowhead).

###### Holotype

**(designated here).** Specimen at 15.3 mm E and 12.7 mm S of the mark on slide 2326, deposited at ANSP accession # ANSP-GC20088. Fig. 11A.

###### Registration.

Phycobank http://phycobank.org/103238.

###### Type locality.

Agat Reef behind Anae I., Agat Bay, 13.357°N, 144.645°E. Epiphytic on seaweed at low tide line, collection number GU75A-4, 6 May 2017. C. Lobban and M. Schefter leg.

###### Additional materials examined.

F.S.M.: Chuuk, TK4!; Marshall Islands. Jaluit Atoll, J5!.

###### Etymology.

Named for its small size within the genus.

##### ﻿ Synedrosphenia recta

Taxon classificationPlantaeArdissonealesArdissoneaceae

Lobban sp. nov.

608DA78F-B66D-52FA-AA28-F02427F112B2

Fig. 13

###### Diagnosis.

Long, straight valves with isopolar, bluntly rounded poles, differing from Synedrosphenia (Ardissonea) crystallina in the marginal annulus, shape of pole, and poration on girdle bands, and from Synedrosphenia (Ardissonea) fulgens in the notch and poration of the valvocopula.

###### Description.

**Figure 13.**
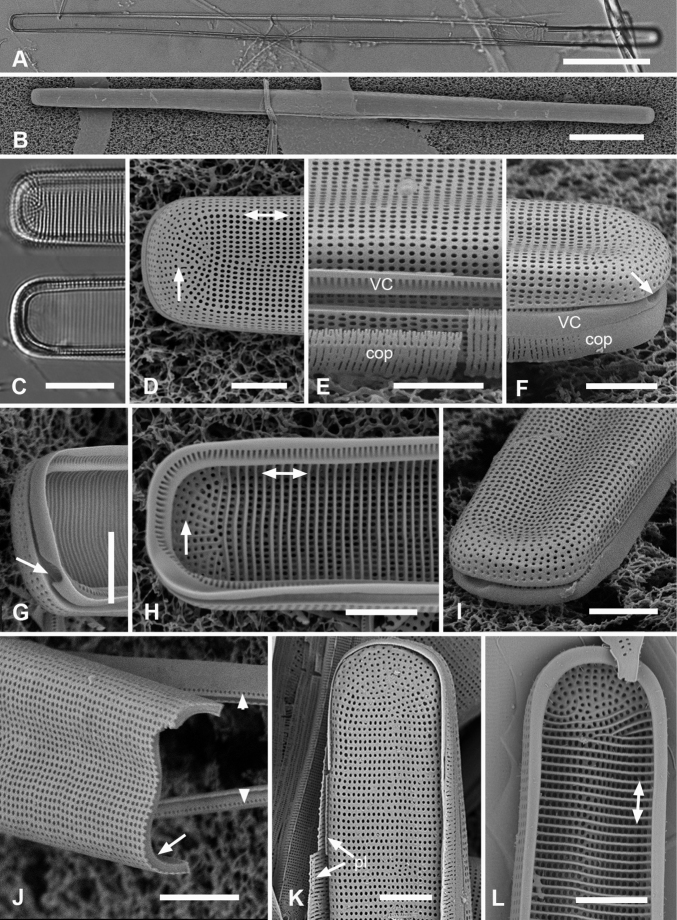
*Synedrospheniarecta* sp. nov. Chuuk TK28 except as noted **A, C** holotype and details of pole at two focal planes, showing valve face and valvocopula **B, D–F** specimen in SEM with details of pole at 0° tilt (**D**), arrows showing position of annulus, and 60° tilted views of central portion (**E**) showing copula and opposite pole (**F**) showing copula and valvocopula with apical notch (arrow) **G, H** internal view of a pole with valve and valvocopula at 60° tilt, showing apical notch (arrow), and at 0° tilt, showing apical area without transapical costae; arrows show inferred position of annulus as in **D** and absence of longitudinal costae **I** polar view of valve with valvocopula, showing apical notch **J** broken valve showing continuous transverse costae (arrow) and pores on pars interior of valvocopula (arrowheads) **K** specimen from Guam (GU76B-4), showing fragments of very narrow pleura (pl, arrows) **L** specimen from Jaluit (J5), pole of valve without valvocopula, showing continuous rim and absence of longitudinal costae; two-way arrow marks position of annulus, as in **D, H**. Scale bars: 50 µm (**A, B**); 10 µm (**C**); 5 µm (**D–L**).

Valves linear, slightly wider in the middle, isopolar with bluntly rounded poles, length 406–430 µm, width 13–16 µm, striae parallel except at pole, 16–18 in 10 µm, areolae slightly elongated in apical axis (Fig. 13A–D). Valve face longitudinally depressed along midline (Fig. 13F, I, K). Annulus detectable externally as a narrow space between areolae around pole and along edge of valve face (Fig. 13D arrows); internally, the end is seen between the areolae at the pole and can be traced around the same as externally (Fig. 13H, L arrows). There are no longitudinal costae (Fig. 13E, J arrow) and transverse costae underlie the vimines as continuous costae from one marginal rim to the other (Fig. 13J). Valvocopula (Fig. 13E–J) lacking external areolae, pores along edge of pars interior have costae between them on the pars interior, appearing to be slits (compare Fig. 13H, J) and the line deflects around the asymmetric sculpting at pole (Fig. 13H). A discrete notch at pole of valvocopula (Fig. 13F, G, I); fimbriae not observed. Copula (Fig. 13E, F) broader with a line along interior–exterior boundary dividing internal bifurcated fimbriae from external part perforated by long slits (rimae) that sometimes continued to the abvalvar edge; perforations discontinued around pole. Pleura glimpsed in Fig. 13K, apparently apical caps joined by very narrow perforated band lacking fimbriae.

###### Holotype

**(designated here).** Specimen at 14.3 mm E and 5.7 mm S of the larger mark on slide 154, deposited at ANSP accession # ANSP-GC20113. Fig. 13A, C.

###### Registration.

Phycobank http://phycobank.org/103239.

###### Type locality.

Federated States of Micronesia: Chuuk: Moch Islet at Eastern Passage of the barrier reef (7°30'50.7"N, 151°57'59.8"E), epiphytic on filamentous seaweeds in farmer fish territory, collection number TK28, 30 May 1991. C.S. Lobban and M. Schefter leg.

###### Additional materials examined.

Guam: GU76B-2!. Marshall Islands: Jaluit, J5!.

###### Etymology.

L. *rectus*, straight. Named for its parallel sides and isopolarity.

###### Taxonomic comments.

This species resembles two former *Ardissonea* species which we are separating into different genera. The complex polar architecture places it in *Synedrosphenia*, within which it is differentiated from S. (Ardissonea) crystallina by the marginal annulus, bluntly rounded poles, plain valvocopula and slits on the copula. The shape of the poles and the marginal annulus resemble *Ardissoneopsisfulgicans*, and in LM of Chuuk samples *S.recta* was distinguishable only by length, the *A.fulgicans* specimens being only 300 µm long. We first noticed it when we found a specimen, apparently of *A.fulgicans*, with a valvocopula pole evidently of the *Synedrosphenia* type (Fig. 13H). It was necessary to tilt specimens to see girdle band characters.

Specimens from Bikar Atoll (not illustrated) with longer (to 583 µm) but narrower (7 µm) valves, weakly inflated at the poles and in the middle, were structurally indistinguishable from the Chuuk specimens on which the species is defined, including the lack of longitudinal costae. Without further evidence, we cannot decide whether to assign this to a formal varietal status, describe it as a separate species, or leave it within the scope of *S.recta*. We mention it here to underline the point that the present study does not exhaust the biodiversity of these genera in Micronesia.

##### ﻿ Ardissonea fulgens

Taxon classificationPlantaeArdissonealesArdissoneaceae

(Greville) Grunow ex De Toni, 1892

A88594BD-30DB-5E45-B452-3E471C935086

Table 3

###### References.

Peragallo and Peragallo 1897–1908, p. 311, pl. 79, fig. 5; Hustedt 1931–1959, p. 228, fig. 717a; Navarro 1982, p. 260, figs 64, 65; Poulin et al. 1986, figs 31, 32; Witkowski et al. 2000, p. 44, pl. 31, figs 9–11; Hein et al. 2008, p. 23, pl. 8: 1, 2).

###### Description from literature.

Kanjer et al. (2021) reported the ultrastructure of Greville’s type material of this species, collected at Mull, Scotland, and clearly showed a pseudoseptum and sculpted apices on the valvocopula; no apical notch was observed. That architecture is not visible in LM and so we can only assume that collections reported from Atlantic and Mediterranean Europe are the same until they can be examined. The species as traditionally understood is described as follows: Valves narrow, linear except slightly wider in the middle, 170–450 µm long, 10–15 µm wide, striae 12–14 in 10 µm, offset slightly where they meet such that a central line (“pseudoraphe”) is distinct but very narrow; longitudinal costae very near to the valve edge and normally difficult to see (Hustedt 1931–1959). Kanjer et al. (2021) show an undulation in the annulus near the middle of the valves in holotype material, a feature not reported elsewhere; they reported a stria density of 15–16 in 10 µm. Poulin et al. (1986, figs 31, 32) showed a valve without pseudoseptum, i.e., of the *Ardissoneopsis* type, with transapical costae on the virgae except at the poles, and the annulus near the valve-mantle junction marked externally by a gap in the areolae and internally by a longitudinal costa on each side. SEMs of var. mediterranea, with 17 striae in 10 µm, in Güttinger (1989), include an oblique view suggesting that the annulus is not internally thickened. Navarro (1982) gave dimensions of 554 µm long by 13.5 µm wide, 14–15 striae in 10 µm. Hein et al. (2008) showed “Ardissoneacf.fulgens” with dimensions of 123–186 µm long by 6.5 µm wide, striae 20 in 10 µm—i.e., smaller and more finely striated than classical descriptions, and they noted that their counts of striae from images in Witkowski et al. (2000) showed stria densities of 19–22 in 10 µm.

###### Taxonomic comments.

For the reasons given below for separating single-walled *Ardissonea* taxa, it cannot remain in *Ardissonea* sensu stricto. If this species is confirmed to lack the apical notch, its pseudoseptum and sculpted valvocopula are sufficiently strong characters to place it in *Synedrosphenia*; it certainly does not belong in *Ardissoneopsis*, proposed below, which lacks those characters, and we therefore must describe our *fulgens*-like taxa as new species and propose a new combination of *Synedrospheniafulgens* for Greville’s species.

#### Group 3: Single-walled taxa without pseudosepta

Table 4

##### ﻿ Ardissoneopsis fulgicans

Taxon classificationPlantaeArdissonealesArdissoneaceae

Lobban & Ashworth sp. nov.

47B36CAE-E5DC-5F93-9929-019FFE2282AB

Fig. 14

Ardissonea fulgens (Greville) Grunow sensu Lobban et al. 2012, p. 259, pl. 16, figs 3–5. Synonym.

###### Diagnosis.

Differing from congeners in the straight outline, only weakly inflated at the center.

###### Description.

**Figures 14.**
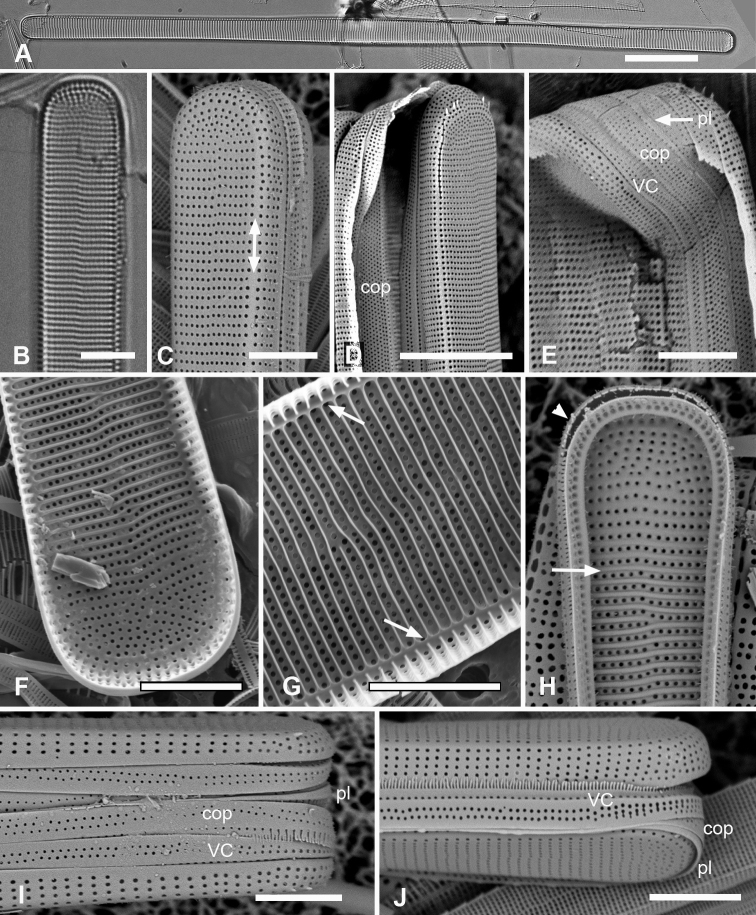
*Ardissoneopsisfulgicans* sp. nov. **A, B** valve in LM showing shape and striation (TK28) **C** pole of frustule from acid cleaned material in SEM, showing annulus (double-headed arrow) (J5) **D, E** poles of frustules in whole mounts, attached at opposite ends, showing girdle bands and apical spines (GU7R) **F, G** internal views of valve pole and central portion, showing the absence of pseudoseptum at pole and presence of longitudinal costae (arrows) (GU44N-A) **H** pole of valve with valvocopula, showing start of transverse costae (arrow) (contrast with pole of *Synedrospheniarecta* Fig. 13H); arrowhead indicates edge of fimbriate copula (J5) **I, J** poles of frustules showing fimbriae on copula and valvocopula, respectively (J5). Scale bars: 25 µm (**A**); 10 µm (**D**); 5 µm (**B, C, E–J**).

Valves 205–320 µm long, 9–15 µm wide, usually slightly widened in the middle and bluntly rounded at poles (Fig. 14A–F); 17–19 parallel striae in 10 µm. Cells attached by one pole to sturdy mucilage stalks (not shown). Annulus along valve–margin junction and not visible in LM, underlain by longitudinal costae (Fig. 14C, D, F, G); sternum absent but striae often out of alignment at the midline (Fig. 14B, H); often spines on poles (Fig. 14D, I). Valve rims straight (i.e., lacking a pseudoseptum) and valvocopula simple, therefore no apical groove (Fig. 14E, F); transverse costae on the mantle thinning toward the margin (Fig. 14G; contrast Fig. 13J). Transverse costae on most vimenes but absent from a relatively large area at the poles (compared to *Synedrospheniarecta*, Fig. 13H) (Fig. 14F, H). Three girdle bands were present (Fig. 14E, I, J). Valvocopula (Fig. 14E, H, J) with generally two rows of pores on pars exterior; pars interior with a single row along outer edge and a fimbriate inner edge. Copula (Fig. 14D, E, I) with multiple rows of pores forming a decussate pattern, and a fimbriate advalvar margin. Pleura barely visible as a narrow cap at each end, possibly connected, apparently fimbriate at the poles (Fig. 14E, I).

###### Holotype

**(designated here).** Slide 449, multiple specimens present. Slide deposited at ANSP, accession # ANSP-GC20111. Representative specimen published in Lobban et al. 2012, Micronesica 43: pl. 16, figs 4, 5.

###### Registration.

Phycobank http://phycobank.org/103241.

###### Type locality.

Guam: Apra Harbor, GabGab reef. 13.443°N, 144.643°E, sparse filamentous algal turf in farmer fish territory (*Plectroglyphidodonlacrymans*), depth ca. 5 m., collection number GU44Y-13, 10 May 2009. C. Lobban, M. Schefter leg.

###### Additional materials examined.

Guam: GU7R!, GU44Z-15!, GU44L!, GU44N-A!, GU44AC-3!, GU44BJ-4! (inter alia); Federated States of Micronesia: Chuuk: TK28!; Marshall Islands: Jaluit J5!.

###### Etymology.

Adjective (Latin), *fulgens* (shiny) + -*icans* (“indicates... resemblance sometimes so close as to be almost identical” – Stearn 1973: 309).

###### Taxonomic comments.

In our region, this species is hard to positively identify in LM from the new species *Synedrospheniarecta* described above, and some other species so far only glimpsed but differs in (1) having simple apical structure unlike *Synedrosphenia* spp.; (2) the presence of two or more rows of pores on the valvocopula exterior. Lobban et al. (2012) previously identified this species as *Ardissoneafulgens* on the basis of literature but Kanjer et al.’s (2021) surprising finding about the type material (see above) forced the reevaluation.

**Table 4.** Comparison of species of *Ardissoneopsis*†.

	Length µm	Width µm	Shape	Stria density in 10 µm	Valvocopula exterior pores	Copula exterior pores	Pleura
*A.appressata* sp. nov.	400–1000	10–15	Linear, center and poles inflated	13–14	2 rows, decreasing to 1	6 rows, decreasing to 4	Very narrow; apical caps?
	[560–900]	[9–12]		[18–19]			
*A.fulgicans* sp. nov.	205–320	9–15	Linear, center slightly inflated	17–19	4 rows, decreasing to 2	4–5 rows	Very narrow; apical caps?
*A.gracilis* sp. nov.	217–431	5–7	Linear, undulate, center ± inflated	10–12	4 rows, decreasing to 1 near basal pole, but increasing at both poles	Ca. 8 rows decreasing to 2	Narrow.; continuous?
*A.undosa*	To 850	10–11	Linear, undulate, center and poles inflated	19–22	Unknown	Unknown	Unknown

† data for known species are from literature cited in text, data for Micronesian specimens of given in brackets where different.

##### ﻿ Ardissoneopsis appressata

Taxon classificationPlantaeArdissonealesArdissoneaceae

Lobban & Ashworth sp. nov.

C1712FC0-814B-5148-99A7-C021F05F91FE

Figs 15 , 16

Ardissoneafulgensvar.gigantea (Lobarzewsky) Rabenhorst sensu Lobban et al. 2012, p. 260, pl. 1, figs 1, 2, pl. 16, figs 6–8. Synonym.

###### Diagnosis.

Differentiated from congeners in the straight valve with inflated center and apices, and valve-appressed colony formation.

###### Description.

**Figure 15.**
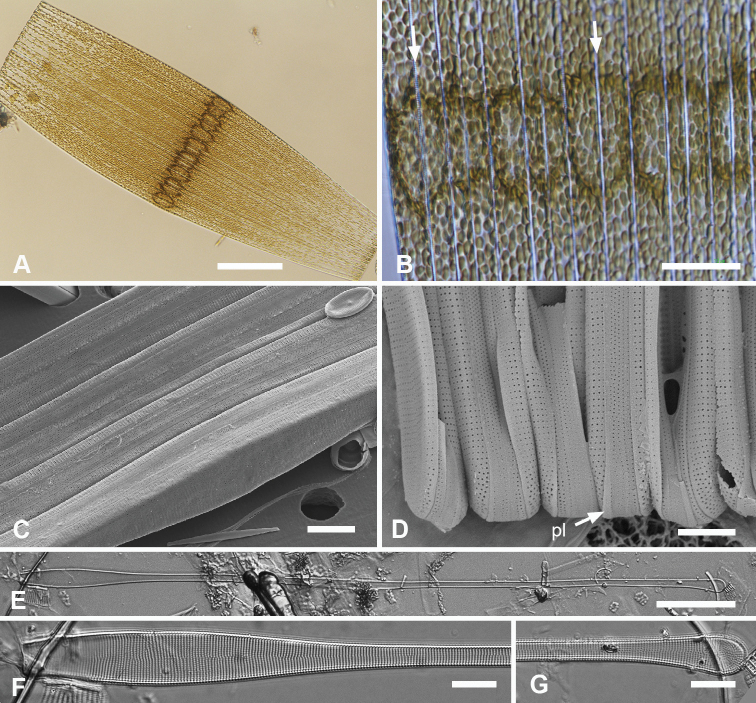
*Ardissoneopsisappressata* sp. nov. **A, B** flabellate colony and detail showing hemispherical nuclei surrounded by plastids and appressed to girdle faces; arrows on **B** show two of the many walls between cells (GU68H-1) **C** middle of colony in oblique view, showing inflation where nuclei are located **D** base of colony, showing parts of six frustules including pleura (pl, arrow) (GU44AP-2) **E–G** half valve from Yap, acid cleaned, with details of central and polar portions showing shape and stria density (Y42-1). Scale bars: 100 µm (**A**); 50 µm (**E**); 25 µm (**B**); 10 µm (**C, F, G**).

**Figure 16.**
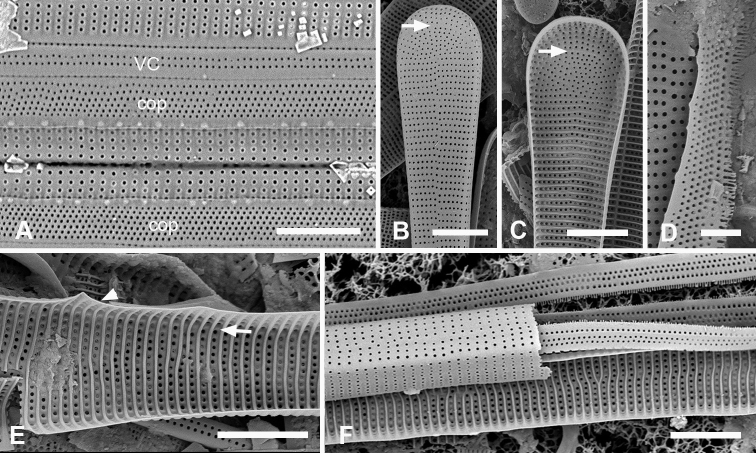
*Ardissoneopsisappressata* sp. nov., cont. GU44BJ-4 except as noted **A** girdle view of part of colony showing cells joined valve to valve, and the pattern of pores in valvocopula (VC) and copula (cop) (GU44AE-2) **B, C** valve pole in external and internal view, respectively showing tip of annulus (arrows) **D** portion of copula showing fimbriae **E** portion of valve near center: arrow points along longitudinal thickening below annulus and arrowhead indicates narrowing of transverse costae at margin **F** portion of frustule in narrow part, showing internal and external aspects and portions of the fimbriate valvocopulae (GU44T-1). Scale bars: 5 µm (**A–C, E, F**); 2 µm (**D**).

Cells valve-appressed to form flat colonies from sturdy mucilage pads, cells toward the outside increasingly curved in the apical axis (Fig. 15A–D). Nucleus in widest part of the cell and appressed to one side, numerous lenticular plastids (Fig. 15B). Valves very long and narrow, inflated at the middle and poles (Fig. 15E–G); frustule also inflated in the middle in girdle view because of deeper mantle and wider girdle bands (Fig. 15C vs D). Length 560–900 µm, width at center 9–12 µm, poles 7–10 µm, 5 µm in between; striae 18 in 10 µm (Figs 15F, G, 16A–C). Annulus along junction of valve face and mantle except at poles, where it is further back (Fig. 16B, C); some thickening under annulus but not as thick as transverse costae (Fig. 16E). Striae parallel except radiate at poles (Fig. 16B, C). Internally, costae on most virgae except near poles (Fig. 16C, E, F). Spines absent. No pseudosepta (Fig. 16C); transverse costae on the mantle diminishing toward margin (Fig. 16E arrowhead; contrast Fig. 13J). Three girdle bands present (Figs 15D, 16A): fimbriate valvocopula (Fig. 16A, F) with two rows of pores around the poles, decreasing to one toward the basal pole, along with one row along bottom edge of pars interior; fimbriate copula (Fig. 16A, D) with six rows of pores in a decussate pattern, decreasing to four near basal pole; pleura (Fig. 15D) very narrow, pars interior not observed.

###### Holotype

**(designated here).** Slide 449, multiple specimens present. Slide deposited at ANSP accession # ANSP-GC20111. Representative specimen published in Lobban et al. 2012, Micronesica 43: pl. 16, figs 6, 7.

###### Registration.

Phycobank http://phycobank.org/103242.

###### Type locality.

Guam: Apra Harbor, GabGab reef. 13.443°N, 144.643°E, sparse filamentous algal turf in farmer fish territory (*Plectroglyphidodonlacrymans*), depth ca. 5 m., collection number GU44Y-13, 10 May 2009. C. Lobban and M. Schefter leg.

###### Additional materials examined.

Guam: GU68H-1!, GU44Z-15!, GU44T-1!, GU44BJ-4!, inter alia. Federated States of Micronesia: Yap: Y42-1!; Chuuk: TK28!.

###### Etymology.

Adjective (L.) *appressus*, appressed, with reference to the colony formation.

###### Taxonomic comments.

We previously (Lobban et al. 2012) identified this species as *Ardissoneafulgens var. gigantea* (Lobarzewsky) Rabenhorst, based on literature. Starting from Peragallo and Peragallo (1897–1908), that taxon has been described as extremely long (400 µm to 1 mm), with a broad central portion joined to capitate poles by very narrow linear portions, the striae 13–14 in 10 µm, but not forming distinctive colonies. Cells from Guam matched this description, except for somewhat higher stria density, but adhered valve to valve while still attached to massive mucilaginous stalks, giving colonies a unique form, a characteristic not mentioned in the literature. The variety is distinctive enough by the shape and colony formation to differentiate it from the nominate variety but has been even less studied than *A.fulgens*.

However, drawings of *Synedragigantea* Lobarzewsky (1840, p. 276, pl. 6, fig. a–c) show a highly elongated diatom occurring in valve-appressed clusters, somewhat similar to those seen in Guam, but not showing any bulge in the middle and being apparently very flexible; as far as one can tell, the frustule is uniformly linear in both views, but he stated that the side (i.e., valve) view is 3–4 times narrower and with a *wavy outline*. This does not well accord with valve views of *Ardissoneafulgens var. gigantea*, which has never been depicted as wavy but has been shown with a strong central inflation. Lobarzewsky’s drawings look more like *Licmophoraflucticulata* Lobban, Schefter and Ruck (2011), which is wavy and very narrow and occurs in valve-appressed clusters, except that the clusters of that species in girdle view are very obviously tapered. The descriptions of this taxon were carried forward into Rabenhorst (1864: 140) and De Toni (1892: 674) but there is no mention of the valve outline. The comparison of its shape to *Toxariumhennedyanum* (W. Gregory) Pelletan seems to have begun with Peragallo and Peragallo (1897–1908), who clearly describe the valve shape and stria density for the first time, but show a smooth valve outline. This taxon was said to be frequent in the Mediterranean (Peragallo and Peragallo 1897–1908), yet neither they nor Hustedt (1931–1959) refer to valves sticking together in distinctive fan-shaped clusters. The extreme length is the sole character that could link the acid cleaned frustules so accurately described by the Peragallos to the taxon drawn by Lobarzewski (1840), and that is insufficient, so that the identity of *Synedragigantea* is a mystery, while the lack of colony information and the lower stria density leave doubt about how the Guam population is related to any European taxa. Given findings of Kanjer et al. (2021) on the nominate variety, we reject our earlier identification and propose a new species for our material.

##### ﻿ Synedra undosa

Taxon classificationPlantaeArdissonealesArdissoneaceae

Grunow, 1867

3632B8D3-3DB8-5448-B165-1EE5E7CBF81F

Fig. 17

###### Reference.

Grunow 1867, p. 4; Grunow 1877, p. 167, pl. 193, fig. 8a–c.

###### Description from literature.

Very long, to 850 µm, slender, inflated at the center and poles, undulate in between, striae 19 in 10 µm (20–22 according to De Toni 1892). *Synedraundosa* has the outline of *Toxariumundulatum*, but linear striae as in *Ardissoneopsisappressata*.

###### Materials examined.

Remnant material of Grunow 839-4611 [W catalog # W0127010, Acquisition # W-1901-0004611]. Not found in Micronesian samples.

###### Observations.

**Figure 17.**
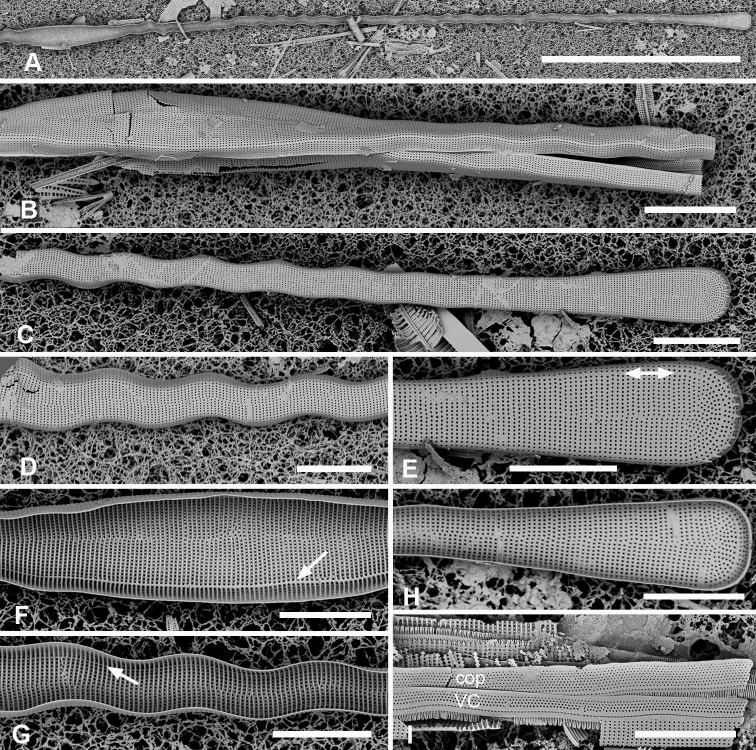
*Synedraundosa* from Grunow’s Honduras material **A** internal view of half valve, total length would have been ca. 690 µm **B** external view of fragment of frustule including the central inflation **C** external view of valve fragment with pole **D** external view of valve fragment near central area showing the strong undulation **E** detail of external pole showing annulus (double-headed arrow) and spines **F–H** internal views of central, near central (cf. **D**), and polar portions of valve, respectively, showing longitudinal costae (arrows) and transverse costae; transverse costae developing gradually toward left in **H**. **I** girdle bands, showing fimbriate valvocopula and copula with glimpses of interior costate surface of valve. Scale bars: 100 µm (**A**); 15 µm (**B, C**); 10 µm (**D–I**).

We have not encountered this in the Guam flora but found several fragments in Grunow’s Honduras gathering that showed internal and external structure, confirming the similarity of structure to the two aforementioned *Ardissoneopsis* spp. A fragment including the central inflation and one pole suggests a total length of ca. 690 µm (Fig. 17A). Widths were 10–11 µm across the center, 9 µm across the poles and 4–5 µm between; stria density 19–21 in 10 µm. The annulus along valve face–mantle junction at least partially subtended by longitudinal costae (Fig. 17B–E). Transverse costae also evident throughout except near poles (Fig. 17F–H). No pseudoseptum. Valvocopula and copula both with multiple rows of pores in decussate pattern on exterior, one row of interior pores, fimbriate inner margin and lacking sculpted apices (Fig. 17I). Copula wider, with fimbriae commonly bifurcated.

###### Taxonomic comments.

According to AlgaeBase (Miranda in Guiry and Guiry 2020), *S.undosa* is currently referred to as *Toxariumundosum* (Grunow) De Toni, 1892, which De Toni recognized as distinct from *T.undulatum*. We have not found more recent mention of this species, but it clearly cannot remain in *Toxarium*. Hustedt (1931–1959, pp 224–225) stated that, while Grunow had made clear the abundance of *Synedraundosa* in his Honduras material, “one must assume he also included S. (Toxarium) undulata,” because Hustedt had not observed specimens matching Grunow’s drawings in Honduras material he examined. Hustedt concluded that this species requires further examination. Grunow, however, was clear that *S.undosa* was different from *S.undulata* and the SEM images here clearly prove him right. On the evidence that it is a species in its own right, we propose below its transfer to *Ardissoneopsis*.

##### ﻿ Ardissoneopsis gracilis

Taxon classificationPlantaeArdissonealesArdissoneaceae

Lobban sp. nov.

BD9DE006-3FAD-5C0E-A316-49B6E2078499

Fig. 18

###### Diagnosis.

Differing from *A.fulgicans* in valves being more slender, slightly wavy outline, lower stria density, and markedly heteropolar frustule in girdle view and from *A.undosa* by lack of central inflation.

###### Description.

**Figure 18.**
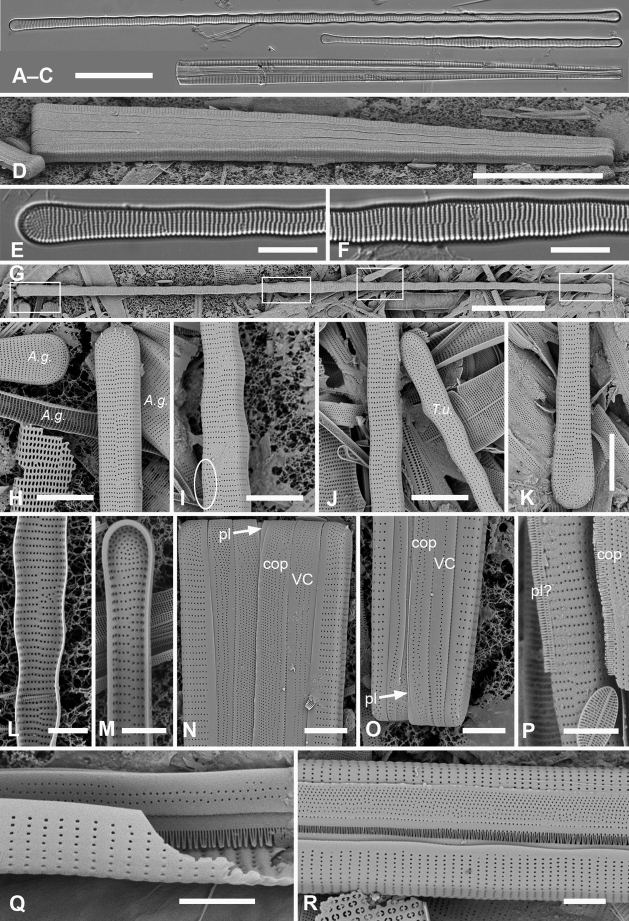
*Ardissoneopsisgracilis* sp. nov. (GU44BJ-4, except **B** TK28) **A–C** three specimens to scale in LM to show size range: valves from Guam (**A**) and Chuuk (**B**), frustule from Guam in girdle view (**C**) showing heteropolarity **D** frustule in SEM tilted 60°, showing both valve and girdle faces **E, F** pole and mid-section of holotype **G–K** external views of valve in SEM with other species adjacent for comparison (GU44BJ-4): index image (**G**) with locations of details marked, apical pole (**H**) (shown by depth of mantle), compare stria density with *Ardissoneopsisappressata* (*A.g.*); top half of center (**I**), offset areolae in striae within the oval suggest location of annulus; narrow portion below center (**J**), compare with scattered areolae in adjacent *Toxariumundulatum* (*T.u.*); basal pole (**K**), note spines on both poles **L, M** internal aspect near middle and at the apical pole, showing lack of costae **N, O** girdle view of frustule apical and basal poles, showing taper in mantle and girdle bands **P** possible piece of pleura (pl?) with fimbriae **Q, R** fimbriate edges of valvocopula and copula, respectively. Scale bars: 50 µm (**A–D, G**); 10 µm (**E, F, H–K**); 5 µm (**L–R**).

Frustules cuneate in girdle view (Fig. 18C, D, N, O), both valve mantle and width of girdle bands decreasing from apical pole to basal pole; hypothesized to be attached to mucilage pads at the basal pole, but not observed in live or raw material. Valves slender, weakly undulating, slightly inflated in the middle and at poles, slightly heteropolar but poles not consistently distinguishable except for mantle depth (Fig. 18A, B, G, E). Dimensions: 206–431 µm long, 5 µm wide except 7 µm across the middle and poles (Fig. 18A–G). Striae parallel, 10–12 in 10 µm, except radiating at poles, areolae small, circular. The annulus near valve–mantle junction, indicated only in places where striae on mantle are offset from those on valve face (oval on Fig. 18I). There is often also an offset across the midline but no sternum. Several spines occur on both poles. (Fig. 18H, K, N, O). Internally, interstriae are barely thickened, not forming costae, and there are no longitudinal costae on the annulus (Fig. 18L, M). Three girdle bands were present (Fig. 18N–R): valvocopula with a row of pores along the edge of the pars interior and a fimbriate internal margin (Fig. 18Q); two rows of pores occur around basal pole, but become one within 10 µm from base, further out becoming two and then three or more rows (Fig. 18N, O). In the copula, also bearing a fimbriate flange (Fig. 18P, R), pars exterior has numerous pores arranged in a generally decussate pattern but becoming looser near basal pole (Fig. 18N, O). The pleura is very narrow even at the poles (Fig. 18N, O, P).

###### Holotype

**(designated here).** Specimen at 11.0 mm E, 2.5 mm S of the mark on slide 1831, deposited at ANSP accession # ANSP-GC20087. Fig. 18E, F.

###### Registration.

Phycobank http://phycobank.org/103243.

###### Type locality.

Guam: Apra Harbor: GabGab reef, 13.443°N, 144.643°E, sparse filamentous algal turf on farmer fish territory (*Plectroglyphidodonlacrymans*), associated with *A.gigantea* and *Toxariumundulatum*, depth ca. 8 m., collection number GU44BJ-4, 8 Feb. 2015. C.S. Lobban and M. Schefter leg.

###### Other materials examined.

Guam: Apra Harbor: GU52P-7!, GU52AI-1!. Federated States of Micronesia: Chuuk: TK28!; Yap: Y36-2!. Marshall Islands: Majuro: M1!, Jaluit, J1!.

###### Etymology.

Adjective (Latin), slender.

###### Taxonomic comments.

Most similar to *Ardissoneopsisfulgicans* but differing in the wavy margin, width only half that of *A.fulgicans*, and in having only 10–12 striae vs. 19–20 in 10 µm. Its shape resembles some *Toxariumhennedeyanum*, which can sometimes be slightly wavy and only weakly inflated in the middle (e.g., Suppl. material 4: Fig. S2A–C) but always has a random pattern of areolae on the valve face. Much less inflated in the middle, much less strongly undulate, and more coarsely striated than *Synedraundosa*.

#### Group 4: *Synedrabacillaris* complex

##### ﻿ Synedra bacillaris

Taxon classificationPlantaeArdissonealesArdissoneaceae

(Grunow) Hustedt, 1932

AAAFC283-B9C8-5AE3-ABFF-DF77E63B7B85

Fig. 19

###### References.

Grunow 1877, p. 167, pl. 193, fig. 12a–c; Hustedt 1931–1959, p. 230, fig. 718; Sullivan and Wear 1995, p. 182, figs 9–12.

###### Description from literature.

As described from LM (Hustedt 1931–1959), this taxon is easily distinguished from *Ardissonea* spp. by the prominent longitudinal costa on the midline and the absence of lateral longitudinal costae (i.e., no observable annulus). There is only one wall layer. Valves linear, usually slightly wider in the middle, 320–700 µm long, 16–20 µm wide, 8–9 striae in 10 µm. Robust transapical costae on each of the virgae. As seen in SEM (Sullivan and Wear 1995), the valve has a single wall, areolae are circular, those at the poles slightly smaller and not organized into striae, elsewhere the striae are framed by the transapical costae. Sullivan and Wear stated that the costae extend from the margin to the median costa, but their fig. 12 shows some costae extending from the median costa and the costae are not consistently thick. Sullivan and Wear reported an absence of spines, polar pore fields and rimoportulae. Girdle bands closed, not more than five (three on the epicingulum): the valvocopula broad with a single row of small, circular pores near the advalvar edge of the pars exterior and a large flange that fits inside the valve mantle; there was said to be short fimbriae along its inner edge but they are not evident in the micrographs. There are additional rows of pores on the valvocopula just around the poles. The valvocopula has a flange that fits around the valve mantle but there is no apical notch. The copula is also broad but with several rows of pores (their fig. 11 shows these loosely organized in straight striae of at least three pores), and there is a narrow pleura (Sullivan and Wear 1995); none of their images show the inner edge of the girdle bands. Although not specifically stated in these descriptions, the frustules are implicitly isopolar. Sullivan and Wear (1995) particularly noted the circular areolae, in contrast to the large oval areolae they saw in *Ardissoneaformosa*. Grunow’s (1877) published drawings (as Synedracrystallinavar.bacillaris) do not include details of areolae but unpublished drawings archived in the Grunow drawing collection at W (W1901-5465) include several sketches where the areolae appear to be oval. Grunow’s drawings, like those of later authors, show this species as linear and isopolar, with bluntly rounded poles, but his unpublished girdle view shows a slightly tapered frustule.

###### Observations.

**Figure 19.**
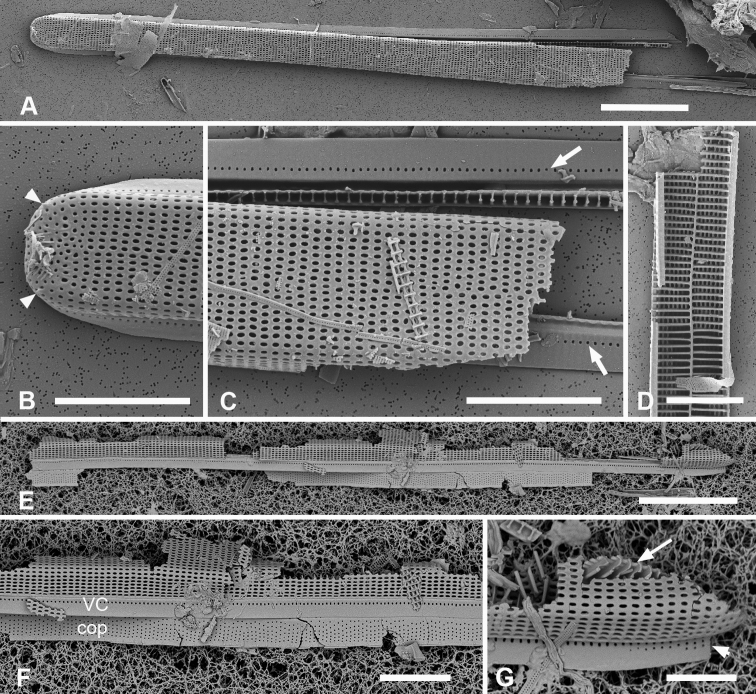
Putative Synedracrystallinavar.bacillaris from Grunow’s Honduras material **A–C** specimen in external view with valvocopula, identified by the length, the straight sides and blunt pole, stria density (11 in 10 µm), and row of pores in the exterior of the valvocopula (arrows); small spines on apex (arrowheads on **B**) **D** fragment of valve, interior view, showing central longitudinal costa and transverse costae **E–G** fragment of frustule showing valvocopula and copula **G** showing transverse costae (arrow) and absence of inner wall, arrowhead shows additional pores on valvocopula pole. Scale bars: 25 µm (**A, E**); 10 µm (**B–D, F**); 5 µm (**G**).

We were able to find a few fragments of Synedracrystallinavar.bacillaris Grunow in an SEM preparation from Grunow’s Honduras gathering (Fig. 19). The external view (Fig. 19A–C), of a fragment 172 µm long that appeared to reach the slightly inflated middle, was distinguished from *Ardissoneaformosa*, also present on the stub, by the row of pores on the exterior of the valvocopula. The pole was blunt, with small spines (Fig. 19B, arrowheads), and the valve linear, although a little wider in the middle. Both valves had 11 striae in 10 µm, comprising oval areolae, and the small fragment, width 10 µm, showing the interior had the characteristic central longitudinal costa and prominent transapical costae (Fig. 19D). A second frustule fragment (Fig. 19E–G), 174 µm long, showed the single line of pores on the valvocopula were 19 in 10 µm, striae on the copula 18 in 10 µm. The broken tip of this valve showed the transverse costae (Fig. 19G).

###### Taxonomic comments.

Specimens provisionally identified as *S.bacillaris* were found in samples from across Micronesia but there was a range of stria densities and dimensions. All had oval areolae and many had spines. However, there were few fitting the literature description, even overlooking the shape of the areolae, i.e., those with linear valves, blunt poles and 8–9 striae in 10 µm. Those in Guam were mostly among populations of lanceolate specimens and probably part of them (see next taxon). The small, circular areolae shown by Sullivan and Wear (1995) are certainly different from the oval areolae in our specimens but determining the identities of these specimens relative to one another and to the literature will require much further study.

##### ﻿ Grunowago pacifica

Taxon classificationPlantaeArdissonealesArdissoneaceae

Lobban & Ashworth sp. nov.

DD1993EC-7F48-516D-9761-3E2302BC05FB

Figs 20 , 21

###### References.

Lobban et al. 2012, pp. 256–257, pl. 13, figs 1, 2 [as *Synedrabacillaris* (Grunow) Hustedt].

###### Diagnosis.

Differing from Grunowago (Synedra) bacillaris in width and lanceolate outline of valve.

###### Description.

**Figure 20.**
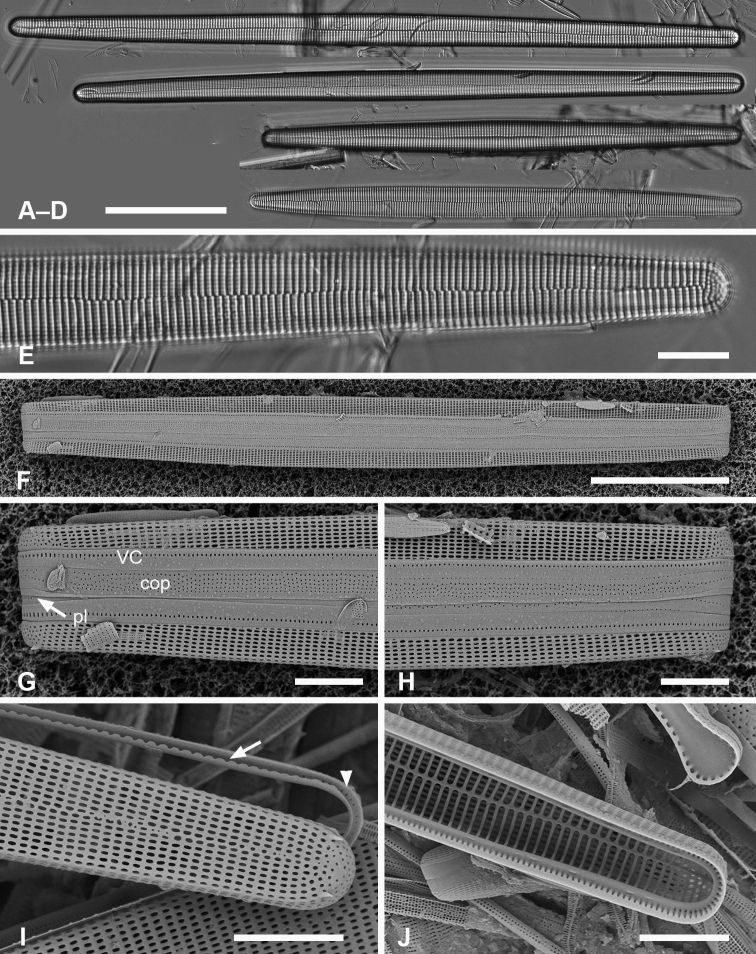
*Grunowagopacifica* sp. nov. **A–D** LM of specimens from GU44BJ-4 (**A–C**) and GU44Z-15 (**D**) **D, E** holotype specimen **F–H** frustule in girdle view, showing isopolar frustule wider in the middle and three girdle bands (GU44BJ-4) **I** external view of pole and advalvar surface of valvocopula, the latter showing a short row of small pores on the apical part of the pars interior (arrowhead) and the crenulated edge of the pars interior (arrow) **J** internal view of pole and abvalvar surface of valvocopula. Scale bars: 50 µm (**A–D, F**); 10 µm (**E, G–J**).

**Figure 21.**
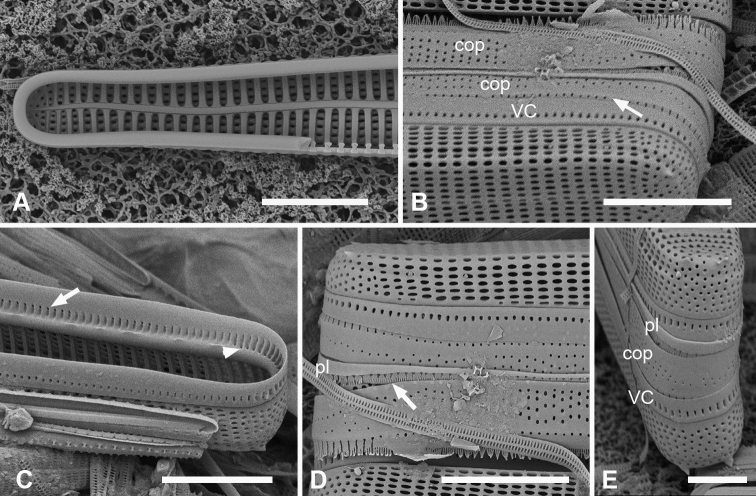
*Grunowagopacifica* sp. nov., cont., all GU44BJ-4, except **A** = SPN2022-1-4 **A** valve apex showing continuous rim, broken away at lower right, not expanded into a pseudoseptum **B** frustule at 40° tilt, showing fimbriate inner margin of copula on the hypotheca (top) and line of pores on pars interior (arrow) **C** valvocopula, showing internal and external aspects, the pars exterior pores (arrow) separated by ridges on the pars interior, and the presence of pores on the pars interior at the pole (arrowhead) **D** frustule in girdle view with fimbriate pars interior of pleura exposed (arrow) **E** frustule in polar view tilted 60°, showing girdle bands. Scale bars: 10 µm (**A–D**); 5 µm (**E**).

Valves lanceolate, often slightly rostrate, 194–341 µm long, 11–16 µm wide, striae 9–10 in 10 µm (Fig. 20A–D), the striae comprising large oval pores; poles with smaller, circular pores, but not organized into polar pore fields (Figs 20E, 21A). Isopolar but wider in the middle in girdle view (Fig. 18F–H). Two small spines usually present on each pole (Figs 20G–I, 21B). Rim present on the valve border but not extended into a pseudoseptum (Fig. 21A). Valve single-walled (Figs 20J, 21A), internally costate with a distinctive central costa the entire length of valve and thickened vimines (Figs 20J, 21A), no annulus detected either as a break in the striae, or any other longitudinal costae (Fig. 21A). Three girdle bands were present (Fig. 20F–H). Valvocopula (Figs 20I, J, 21A, C) without extended lip but having groove on pars interior that fits rim of valve. A single row of pores on advalvar side of pars exterior, circular except elongated near poles (Figs 20G, H, 21A, C), a short line of pores around poles on pars interior (Figs 20I, 21C). Copula has a row of pores on outer edge of pars interior (Fig. 21A, B, D), often hidden under valvocopula and a well-developed fringe on the pars interior; up to 5 rows of pores on pars exterior (Figs 20G, H, 21B, D). Pleura a very thin fimbriate band, continuous along cell length (Figs 20G, H, 21B, D).

###### Holotype

**(designated here).** Specimen at 18.3 mm E and 6.4 mm S of the mark on slide 146, deposited at ANSP accession # ANSP-GC20085. Fig. 20D, E.

###### Registration.

Phycobank http://phycobank.org/103245.

###### Type locality.

GabGab reef, Apra Harbor, Guam, 13.443°N, 144.643°E, ca. 1 m depth, collection number GU44Z-15, 20 June 2009. C.S. Lobban and M. Schefter leg. Additional slides from this sample have previously been deposited at California Academy of Sciences (CAS) for other species holotypes, but specimens of *G.pacifica* are not marked. Slides include CAS accession/slide numbers: 627386/223007 (*Perideraion* spp.), 627383/223005 (*Gatohyalinus*); 627409/223023 (*Astrosyneradiata*); and 627396/223010 (*Hanicellamoenia*).

###### Additional materials examined.

Guam: GU44L-C!, GU44AI-5! (in culture), GU44AP-8!, GU44BH-5!.

###### Etymology.

Adjective (Latin) with reference to its presence in the Western Pacific Ocean.

###### Taxonomic comments.

Specimens that we (Lobban et al. 2012) referred to *Synedrabacillaris* were 180–400 µm long, 14 µm wide, with 9–14 striae in 10 µm. This range of stria density is suspiciously large for Ardissoneaceae and suggests that two or more species are being confounded. Some specimens in GU44BJ-4 population were nearly linear but most likely represent an extreme within *G.pacifica* rather than an occasional *G.bacillaris*. Both Hustedt (1931–1959) and Sullivan and Wear (1995) remarked on the ease of identifying this species by the strong central costa, compared to other *Ardissonea* spp., but as with other taxa where a strong identifying character has masked species diversity (e.g., *Cyclophoratenuis* – Ashworth et al. 2012, *Bleakeleyanotata* – Lobban and Perez 2016), we suspect that there is hidden diversity within “*Synedrabacillaris*”. The genetic branches are long between lanceolate samples from Guam (GU44AI-5) and Florida (HK291) (see, below, Fig. 33). For now, we can only describe *Grunowagopacifica* and deal with the generic placement of that species cluster.

#### Group 5: Genus *Toxarium*

##### ﻿ Toxarium hennedyanum

Taxon classificationPlantaeArdissonealesArdissoneaceae

(Gregory) Pelletan, 1889

04308621-E308-5D52-822B-325D51F2A01D

Fig. 22

###### References.

Hustedt 1931–1959, p. 222, 224, fig. 713 (as *Synedrahennedyana*); Round et al. 1990, p. 422–423; Lobban et al. 2012, p. 260, pl. 17, figs 1–5; Kooistra et al. 2003, fig. 1A–R (as *T.undulatum*); Hein et al. 2008, p.33, pl. 15, fig. 3; Álvarez-Blanco and Blanco 2014, p. 128, pl. 63, figs 4–6.

###### Description from literature.

**Figure 22.**
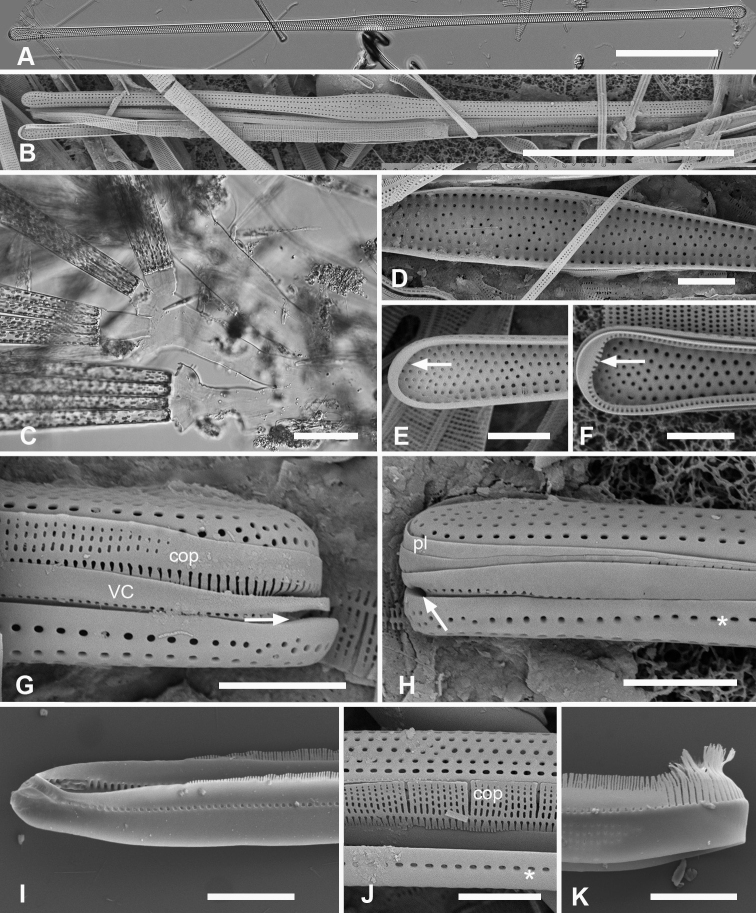
*Toxariumhennedyanum*, all from GU44BJ-4 except **C** GU21AK-11, and **I, K** culture from GU44AK-4 **A** LM of valve with typical pattern in central area; 9 stria in 10 µm **B** SEM of a valve from same sample, 12 striae in 10 µm, with reduced central area **C** mucilaginous stalks with clusters of cells **D** internal central area, showing lack of costae **E, F** internal pole of valve showing asymmetrical pseudoseptum (arrow) and valve with valvocopula showing similar asymmetry (arrow) **G** pole of frustule in girdle view showing apical groove (arrow) on valvocopula and fimbriae partially exposed on copula **H** pole of frustule in girdle view showing apical notch (arrow) and the pleura (pl); note single row of areolae on mantle (*) **I** pole of valvocopula in girdle view, fimbriae absent from the tip **J** narrow portion of valve showing structure of copula (compare with Fig. 23J) and single row of pores on mantle (*) **K** pole of copula in girdle view showing long fimbriae. Scale bars: 50 µm (**A–C**); 10 µm (**I**); 5 µm (**D–K**).

**Figure 23.**
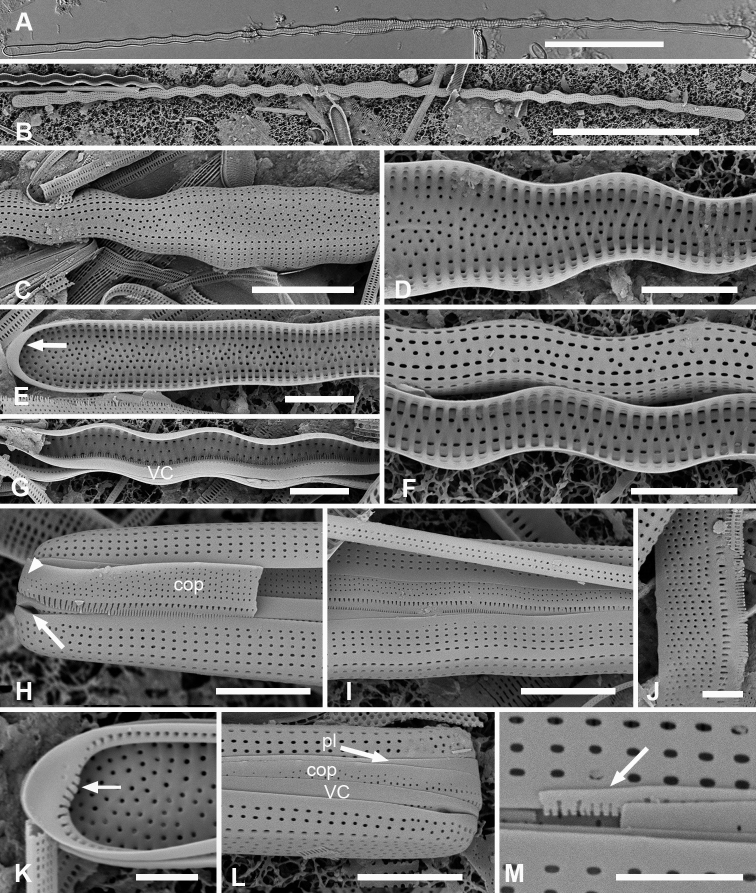
*Toxariumundulatum* (all from Guam, GU44BJ-4) **A, B** entire valves in LM and SEM, respectively, showing prominent undulation **C, D** central expanded portion external and internal views, respectively, the latter showing costae **E** pole, internal view showing asymmetrical pseudoseptum (arrow) **F** internal and external aspects of valve in the narrow part **G** valvocopula lying inside narrow part of valve **H, I** portion of frustules in girdle view, showing multiple rows of pores on mantle, valvocopula with apical notch (arrow), and copula with pores continuing around the pole (arrowhead) **J** detail of copula, for comparison with Fig. 22J**K** pole of valvocopula showing arched slits **L** frustule in girdle view showing pleura **M** fragment of the pleura, showing fimbriae. Scale bars: 50 µm (**A, B**); 10 µm (**C**); 5 µm (**D–I, K**); 2 µm (**J, K, M**).

Colonial on stout mucilage stalks. Long, narrow valves, usually straight, inflated at the poles and the middle, valve lengths ranging from 300 to >1000 µm (Álvarez-Blanco and Blanco 2014); widths 6–8 µm in the middle, 5–6 µm at poles, and about 2 µm elsewhere; transapical striae very short, 9–11 in 10 µm, with scattered areolae in wider central and polar areas (Hustedt 1931–1959). Lobban et al. (2012, pl. 17, figs 4, 5) illustrated variants with few/no pores in the central part of the valve face. Girdle bands are all closed (Kooistra et al. 2003). The valvocopula has a single row of pores along junction between the pars interior and pars exterior and an asymmetrical, notched shelf that fits against the pseudoseptum. The copula has striae that are either porate or become slit-like with broadly fimbriate margins of the pars interior (compare their fig. 1I, L with our Figs 22J, 23J). The narrow pleura has a single row of pores along the junction between the pars interior and pars exterior.

###### Materials examined.

Guam: GU21AK-11!, GU44AK-4!, GU44BJ-4!. Federated States of Micronesia: Chuuk, TK28!. Marshall Islands: Majuro: M1!; Jaluit: J5!; Bikar: BA2!; inter alia.

###### Observations.

Valves 156–334 µm long, usually straight but sometimes curved, 7 µm wide at middle, 4–5 µm at poles, with a single row of areolae on the shallow mantle (Fig. 22H, J, asterisk) and two rows before the scattered areolae (Fig. 22B, D, I), making the short transverse striae 3 areolae long; stria density 10–14 in 10 µm (Fig. 22A, B, D, I, J). In the context of the present analysis, we confirm the presence of an asymmetrical pseudoseptum (Fig. 22E), asymmetrical valvocopula poles with apical notch (Fig. 22F–H), valvocopula finely fimbriate (Fig. 22I), and the copula structure tending toward slits, the striae 28 in 10 µm at the poles (Fig. 22G) increasing to 36 in 10 µm with broadly fimbriate pars interior (Fig. 22J). There is a knuckle-like series of arched slits on the valvocopula pars interior (Fig. 22F), different from the structure in *Synedrosphenia* (compare with Fig. 8B and Fig. 10D, E, G). Spines absent. We note that the pars exterior of the copula is hyaline around the poles. However, the fine fimbriae on the pars interior of the valvocopula (Fig. 22I) are absent from the specimens in Kooistra et al. (2003 fig. 1J, K). A pleura was present (Fig. 22H), the pars interior also fimbriate (not shown).

###### Taxonomic comments.

Kooistra et al. (2003) show a single row of pores on the mantle and a copula that matches those shown here for *T.hennedyanum*. They do not state a stria density, but we count 11 in 10 µm, measured from their fig. 1E. There has been a tendency to ignore stria density, reflecting the marginal striae and the domination of the valve face by scattered areolae. Kooistra et al. (2003) had monoclonal cultures in which both *hennedyanum*-like and *undulatum*-like cells were present, and they proposed a merger of the two species. The specimens they show had a rippled edge, but not a strongly wavy outline (contrast Poulin et al. 1986; Hein et al. 2008, and our Fig. 23); i.e., a midline along the apical axis is straight, whereas that in *T.undulatum* is sinusoidal. If the Micronesian specimens shown here are indeed *T.hennedyanum* and *T.undulatum* (by no means certain), our results would suggest that the rippled edge seen in the cultures of Kooistra et al. (2003) was a phenotypic expression of *T.hennedyanum* and was not *T.undulatum.* We previously noted (Lobban et al. 2012) the significant genetic variation in Guam isolates of *T.hennedyanum* and *T.undulatum* reported by Theriot et al. (2010, 2011) and this is even more evident now; however, in reviewing the vouchers, we find no data from specimens attributable to *T.undulatum*; all the variation shown in Fig. 33 appears to be part of a *T.hennedyanum* complex.

##### ﻿ Toxarium undulatum

Taxon classificationPlantaeArdissonealesArdissoneaceae

J.W. Bailey, 1854

A210AE75-ECEF-5573-850A-A7639394CD10

Figs 23 , 24

###### References.

**Figure 24.**
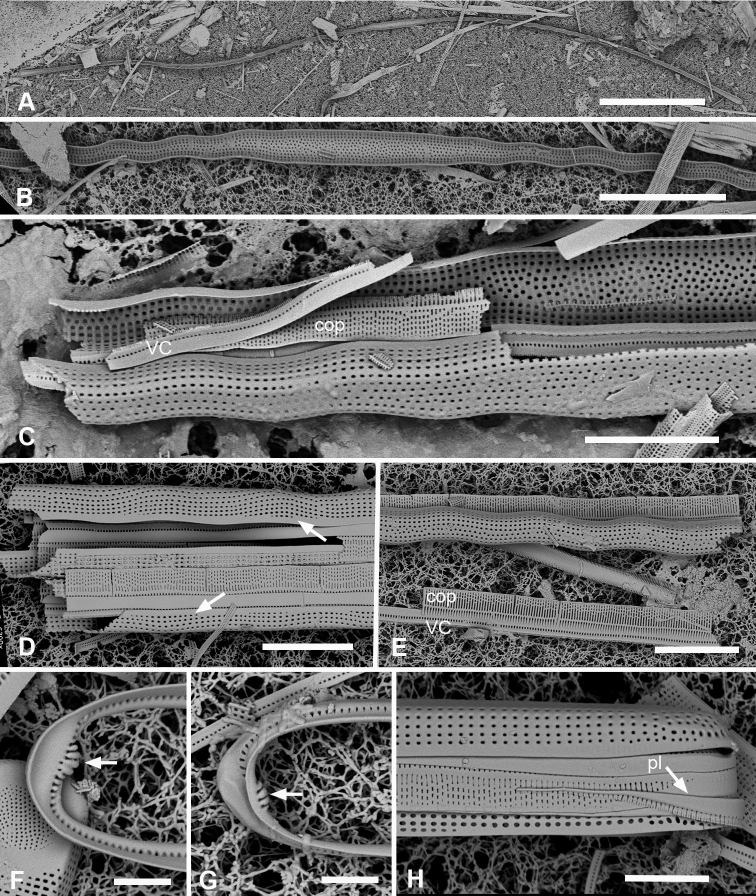
*Toxariumundulatum* from Grunow’s Honduras material **A, B** entire valve, 700 µm long, and detail of central portion, showing long-wavelength undulations **C** portion with the central inflation, showing mantle depth, internal costae, valvocopula and copula (stria density 14 in 10 µm) **D** frustule in girdle view, showing vertical wave in rows of areolae with inserted arcs (arrows); notice also the different depths of the two mantles (stria density 17 in 10 µm) **E** portion of valve with copula and valvocopula **F, G** valvocopula pole, abvalvar and advalvar surfaces, respectively, showing series of arched slits (compare with Fig. 22F) **H** pole of frustule showing fimbriate pleura and notch in valvocopula. Scale bars: 100 µm (**A**); 25 µm (**B**); 10 µm (**C–E**); 5 µm (**F–H**).

**Figure 25.**
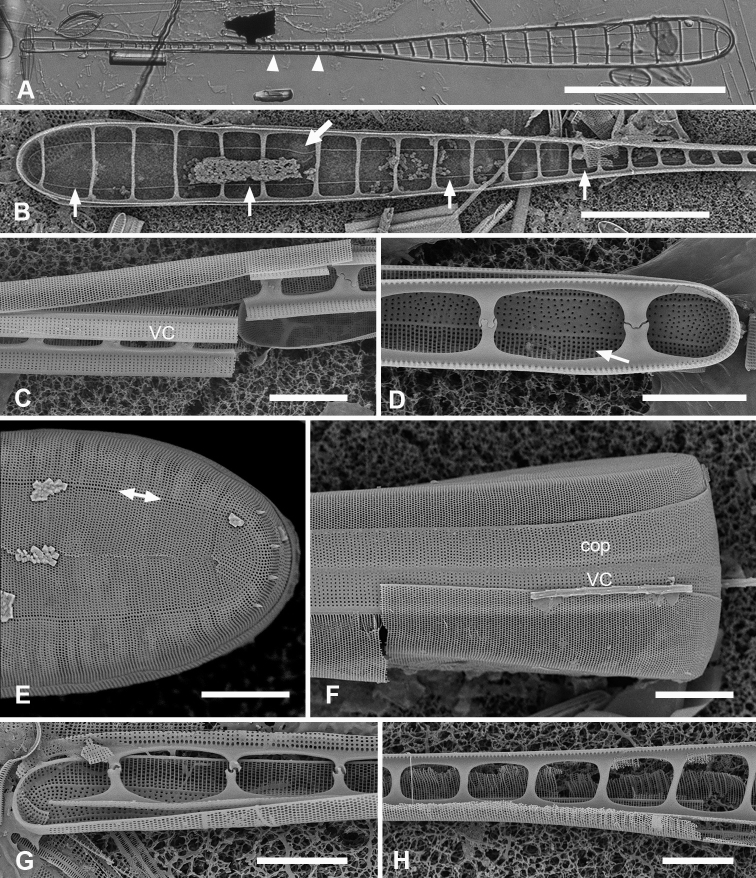
*Climacospheniaelongata*, GU44BM-7, except as noted **A** valve with valvocopula in LM, showing consistently narrow craticular bars, some double (arrowheads) (GU44X-2) **B** apical part of a valve with valvocopula in SEM, showing the progressive narrowing of the annular ring (series of small arrows) and the wider space between craticular bars where the nucleus resides (thick arrow) **C** narrow portion of valve with valvocopula showing the fibmbriae **D** basal pole of valve with valvocopula, showing the scattered pattern of areolae inside the annulus gradually becoming regular striae, development of costae (arrow), and complex unions in the craticular bars **E** external view of apical pole showing annulus (double-headed arrow) and spines **F** frustule in girdle view showing the two girdle bands **G, H** valvocopula near basal pole and in tapering part at top of “stem,” respectively, showing complex versus simple unions in the craticular bars (TK4). Scale bars: 100 µm (**A**); 50 µm (**B**); 10 µm (**C–H**).

Bailey 1854, p. 15, plate (1), figs 24, 25; Peragallo and Peragallo 1897–1908, p. 314, pl. 78, fig. 7; Hustedt 1931–1959, p. 222, 224, fig. 714; Güttinger 1991 (both as *Synedraundulata*); Poulin et al. 1986, figs 33–36; Hein et al. 2008, p. 33, pl. 15, fig. 3; Lobban et al. 2012, p. 260, pl. 17, figs 6–8; Álvarez-Blanco and Blanco 2014, p. 128, pl. 63, figs 4–6.

###### Description from literature.

*Toxariumundulatum*, the generitype, was said to differ only in the wavy outline of the valve (Hustedt 1931–1959), stria density rarely reported but Peragallo and Peragallo (1897–1908) state 12 in 10 µm. The outline in Bailey’s (1854) plate is shown as undulating with a long wavelength, in Peragallo and Peragallo (1897–1908), with a shorter wavelength, and in Hustedt (1931–1959) the center clearly has a wavy edge but the “horns” are scarcely undulate. Micrographs in recent works (Witkowski et al. 2000; Hein et al. 2008) show distinctly undulate outlines. In the SEMs shown by Poulin et al. (1986) we count 19–21 striae in 10 µm, whereas Güttinger (1991: 2.01.30-10) shows specimens with lengths of 485 and 580 µm, widths 8 µm at center, 5 µm at poles, but with 12 striae in 10 µm. Lobban et al. (2012) reported lengths of 270–400 µm, widths 5–9 at the center, 6 µm at poles, 4 µm in between, with some specimens having virtually no central inflation; no stria density stated but their fig. 7 shows 12–13 striae in 10 µm. *T.undulatum* has usually been illustrated as straight but curved specimens are shown by Hein et al. (2008) and Lobban et al. (2012). The genus was emended by Poulin et al. (1986), whose SEM images of *T.undulatum* show an asymmetrical polar pseudoseptum and internal transverse costae “in parts of the valve between the poles and the center and sometimes anastomosing” (also shown in Güttinger 1991). The *T.undulatum* from Guam (GU44S / HK210 / ECT 3802) used by Ruck et al. (2016) showed very shallow undulations, at least after culture, and additional images of the voucher stub show that it has a single row of areolae on the mantle (Suppl. material 4: Fig. S2A–C), so we would therefore identify it as *T.hennedyanum*. A similar specimen from wild material is also shown in the Suppl. material 4: Fig. S2D–F.

###### Materials examined.

Guam: GU44X-2!, GU44Z-15!, GU44BJ-4!, inter alia; Federated States of Micronesia: Chuuk, TK28!; Yap, Y36-2!, Y42-3!. Marshall Islands: Majuro, M1!, Jaluit, J5!

###### Observations.

Valves straight or commonly curved overall, strongly sinusoidal along the narrow part, the oval central portion with a wavy outline, apical portions straight sided (Fig. 23A, B, F), length 255–326 µm, width 5 µm at poles, 7 µm at the center, 3 µm in between, stria densities 14–17 in 10 µm (Fig. 23C–I). Mantles deep with typically 3–4 areolae in the stria there (Fig. 23H, I, K). Spines absent. Valvocopula plain except for single row of pores along the base of the pars interior, fimbriate edge to pars interior (Fig. 23G), knuckle-like structure at poles, as in *T.hennedyanum* (Fig. 23K, compare with Fig. 22F). Copula with a regular pattern of small areolae on the pars exterior, forming striae consistently 34 in 10 µm (Fig. 23H–J, L), in which the pars interior, while similar to that of *T.hennedyanum* near the poles, quickly becomes a fringe of short, narrow fimbriae with a line of triangular pores along the junction with the pars exterior (Fig. 23H). The narrow pleura was largely or entirely obscured (Fig. 23L) (this pole identified as *T.undulata* by 2+ rows of areolae on the mantle, and pores on the poles of the copula; contrast Fig. 22H); a small exposed portion showed a fimbriate edge (Fig. 23M).

We also documented *T.undulatum* in Grunow’s Honduras sample (Fig. 24). A whole valve was 700 µm long (Fig. 24A) with long-wavelength undulations. The deep mantle sometimes exhibited longitudinal waves in the areolae (not solely an artefact of the wave in the valve, shown by short additional longitudinal lines of areolae—Fig. 24D, arrows). The striae on the valve face were two areolae long on each side bordering scattered areolae in the inflated center and poles (Fig. 24C, E), internally, the virgae were thickened into costae (Fig. 24C). Valvocopula (Fig. 24C, E–G) with a single line of areolae on the exterior, short fimbriae along the interior margin except at the sculpted poles, and a series of arched slits (Fig. 24F, G; compare Fig. 23K). Copula (Fig. 24C–E, H) wide with coarse fimbriae on the pars interior, forked at the tips, exterior with striae of pores tending to merge into slits, especially on the advalvar side. Fimbriate pleura clearly seen in Fig. 24H.

###### Taxonomic comments.

The specimens from Grunow’s Honduras material are near the length (“about 35m” = 890 µm; see Itaki and Bjørklund 2006) and shape described by Bailey (1854) for specimens from *Sargassum* in Narragansett Bay. As noted by Hustedt (1931–1959), Grunow (1877) did not mention this species (in the context of presenting new species), but Hustedt says he observed it on Grunow’s slides.

In the Micronesian samples, depths of the mantle, and structure of the copula were different in the two species, so we can assert that the specimens shown here for *T.hennedyanum* and *T.undulatum* are different species. The large genetic variation in *Toxarium* materials we have sequenced (Fig. 33) casts doubt on whether either of them might be identified to authentic Atlantic types, a question highlighted by the Grunow specimens. It similarly raises questions about the number of species there might be in Micronesia. Both questions require much more work to define the range of character states and their combinations, but the oft-repeated claim that the two species differ only in outline is already not supported by morphological evidence. The more important point for the present study is to establish generic characters for comparison to those of the genera in Ardissoneaceae and Climacospheniaceae.

The *Toxarium* spp. specimens shown here from sample GU44BJ-4 were in a community with the new species *Ardissoneopsisgracilis* (see above), which has a slightly undulate outline but is distinguished from *T.undulatum* by the cuneate girdle view, regular striae across the valve face, apical spines, and the decussate pattern of pores on the girdle bands (Fig. 18). Also present were *Synedrospheniagomphonema*, *S.crystallina*, *Ardissoneopsisfulgicans*, *A.appressata*, and *Grunowagopacifica*.

#### Group 6: Genus *Climacosphenia*

Table 5

##### ﻿ Climacosphenia elongata

Taxon classificationPlantaeArdissonealesArdissoneaceae

J.W. Bailey, 1854

621983A0-CE40-5866-A074-AF54574EAC94

Fig. 25

###### References.

Bailey 1854, p. 353, pl. 1, figs 10, 11; Peragallo and Peragallo 1897–1908, p. 352, pl. 86, figs 2–4; Hustedt 1914, pl. 307, figs 1–4; Ricard 1977, figs 669–677; Montgomery 1978, pl. 114, fig. E–H; Round 1982, figs 1–38 (in part); Round et al. 1990, p. 442.

###### Description from literature.

*C.elongata* has been described (e.g., by Peragallo and Peragallo 1897–1908 and Hustedt 1931–1959) as identical to *C.moniligera* Ehrenberg, 1843 except for the valve tapering more abruptly to a stem with parallel sides. Yet Bailey (1854) had specifically distinguished his species from *C.moniligera* by the finer stria density (without giving numbers) and described a species 330 µm long, with very wide craticular bars (as shown also in Peragallo and Peragallo 1897–1908, pl. 86, fig. 5; Hustedt 1914, pl. 307, figs 1–4; and Round 1982, fig. 3) (Table 5). Peragallo and Peragallo (1897–1908) gave stria densities of 18–21 near the basal pole, 27–>30 near the apical pole for *C.elongata* versus 16–17 in 10 µm basal and 19–20 in 10 µm apical for *C.moniligera* but expressed the opinion that there was intergradation between the two species. Hustedt (1931–1959) said that Peragallo and Peragallo had confused their European specimens of *C.elongata* with *C.moniligera* and he attempted to resolve the confusion with an original drawing of a cell with a very long, very narrow stem. The drawing (his fig. 626) was based on Samoan specimens he had published in “Schmidt’s Atlas” (Hustedt 1914). Round (1982) attempted to clarify the difference between the two species, not considering the variation within *C.elongata*, but his drawings of *C.elongata* show specimens no more than 320 µm long. In recent literature, Álvarez Blanco and Blanco (2014), show short cells similar those in Peragallo and Peragallo (1897–1908), while Navarro and Lobban (2009) reported cells 750–1200 µm long from a Guam sample, similar in shape to those in Hustedt (1931–1959), a range that otherwise occurs only in Hustedt’s report (table in Álvarez Blanco and Blanco 2014: 103). Ultrastructural characters: Apical spines present; costae generally present; frustules have only two girdle bands, both with fimbriate inner margins (Ricard 1987, fig. 677; Montgomery 1978, pl. 114, fig. G; Round 1982).

**Table 5.** Comparison of *Climacosphenia* species from literature and present observations.

	Valve length, µm	Width near basal pole, µm	Shape	Valvocopula width near apical pole†, µm	Annulus width near apical pole†, µm	Stria density in 10 µm	Craticular bars
*C.elegantissima*	700–1305	10	Spathulate	22–24	8	18–21 basal, 27–28 apical	Narrow throughout, complex unions in stem often double
*C.elongata* ‡ (literature)	325–525	–	Clavate	–	–	18–21 basal, 27–30 apical	± Narrow throughout, complex unions in stem
*C.elongata* (this study)	278–516	8–9	Clavate	30–31	14–18	22 basal, 28 apical	± Narrow throughout, complex unions in lower stem
*C.moniligera* ‡ (literature)	200–700	10	Gradual taper	15–40	–	16–17 basal, 19–20 apical	Wide throughout; complex unions ?throughout
*C.scimiter*	390–612	9	Curved	25–26	7–10	21 basal, 29 apical	Narrow throughout, complex unions absent
*C.soulonalis*	390–530	10–12	Gradual taper	29–35	15	18–20 basal, 28–29 apical	Narrow throughout, complex unions only in basal 1–3 bars

† We have tried to standardize width measurements for comparison with annulus width by measuring width across the valvocopula rather than the valve because the mantles are deep and tend to spread out, but width data for *C.moniligera* are from the literature. ‡ Confusion between *moniligera* and *elongata* in the literature (see text) has led to a wide range of morphometrics; here we have summarized dimensions from Bailey (1854) and Peragallo and Peragallo (1897–1908) for these species, and craticular bars from drawings in Ehrenberg (1853, pl. 2, part 6, fig. 1a, b), Peragallo and Peragallo (1897–1908, plate 86, fig. 5), Hustedt (1914, pl. 307, figs 1–4), and Round (1982, fig. 3). Navarro et al. (1989, fig. 11) and Witkowski et al. (2000, pl. 18, fig. 1) also show recognizable *C.moniligera* in LM.

###### Materials examined.

Guam: GU44Y-13!, GU44U-1B!, GU44BM-4!, GU44BM-7!, GU52X-1!. Federated States of Micronesia: Chuuk, TK4!

###### Observations.

Valve clavate, apical part gradually tapering to a narrow stem nearly half the total length of 278–516 µm (Fig. 25A, B); maximum width 30–33 at apical pole, 7–8 µm across stem and expanding slightly to 9 µm across basal pole (Table 5); annulus closed at both poles, gradually tapering from maximum width of 16–17 µm near apical pole (Fig. 25B arrows, D, E). Stria densities 28 in 10 µm near apical pole (Fig. 25E), 22 in 10 µm near basal pole outside the annulus; sparser and more loosely organized inside annulus at basal pole (Fig. 25D, G). Apical spines present (Fig. 25E), pseudoseptum absent (Fig. 25G). Costae present except at basal pole, where they start at margin and extend centripetally, eventually thickening the virgae inside the annulus (Fig. 25G). Valvocopula stria density 19 in 10 µm (Fig. 25E, F), pars interior with comb of long, unbranched fimbriae (Fig. 25C). Craticular bars consistently narrow (1 µm) (Fig. 25B, D, G, H), though sometimes doubled (Fig. 25A), the spaces between them rectangular with rounded corners; connections complex in most of the stem, becoming simple where the valve widened (Fig. 25C, D, G vs Fig. 25B, H). There was a wider space between two of the bars in the middle of the wide part (Fig. 25B, thick arrow); based on our observations on *C.elegantissima* (below), this is probably the location of the nucleus. Copula with 24 striae in 10 µm (Fig. 25F), pars interior not observed.

###### Taxonomic comments.

The shape and morphometrics accord adequately with the literature on *C.elongata*, the narrow craticular bars, especially in the stem, differing from the sturdy bars and oval spaces of *C.moniligera*, and from the very long-stemmed specimens depicted by Hustedt (1931–1959, p. 89, fig. 626). Based on the available evidence, we accept the hypothesis that the specimens shown in Fig. 25 are Bailey’s species.

##### ﻿ Climacosphenia elegantissima

Taxon classificationPlantaeArdissonealesArdissoneaceae

Lobban & Ashworth sp. nov.

68B61697-4DD9-56C5-9D7D-9AEC4B97CB5B

Figs 26 , 27 , 28

###### References.

?Hustedt 1914 in Schmidt et al. 1874–1959, pl. 308, figs 5–10; ?Hustedt 1931–1959, fig. 626; Navarro and Lobban 2009, p. 136, figs 64, 65 (all as *C.elongata* Bailey).

###### Diagnosis.

Differing from *C.elongata* in the greater length, especially in the stem, and narrower, linear apical portion, and from *C.truncata* Hustedt ex Simonsen in the shape of the wide part of the valve.

###### Description.

**Figure 26.**
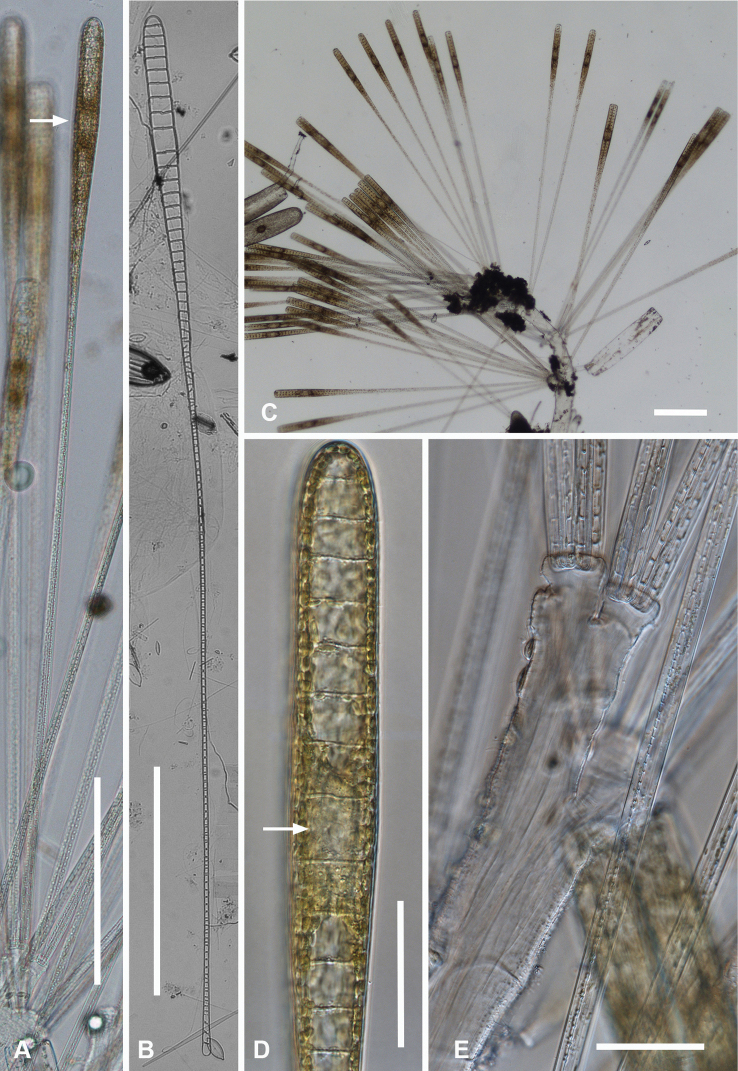
*Climacospheniaelegantissima* sp. nov., (GU52AI-1 except **B** from GU52U-2) **A, D** living cell, showing extreme length and location of nucleus (arrow) **B** acid-cleaned complete valvocopula, emphasizing the very long, narrow stem **C** portion of colony, showing cells attached to mucilage stalk; the two cells to right of middle are 1300 µm long **E** detail of mucilage stalk with bases of attached cells. Scale bars: 250 µm (**A–C**); 50 µm (**D, E**).

**Figure 27.**
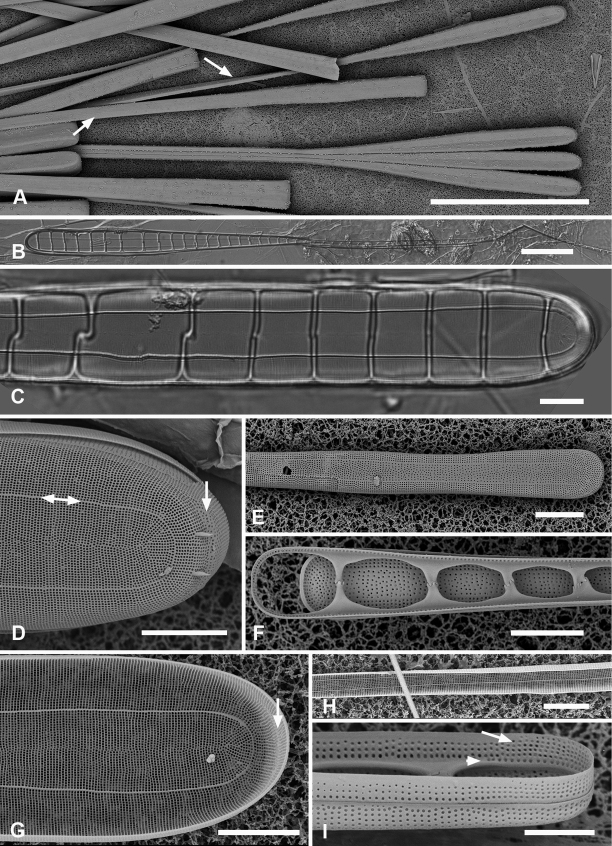
*Climacospheniaelegantissima* sp. nov., cont. GU52AI-1, except GU52U-2 (**B**) and GU52X-1 (**G, H**) **A** whole mount of colony in SEM, showing contrasting width versus depth of frustule in the stem (arrows at approximately same distance from apical poles) **B** portion of holotype in LM; although specimen is broken in two places, the basal pole of the valvocopula is also present, total length ca. 835 µm **C** apical pole of valve and valvocopula in LM, showing parallel sides of annulus and some misaligned unions in the craticular bars **D** SEM of external valve surface at apical pole, showing annulus (double-headed arrow) continuing around apical pole, two spines, and crescent of scattered areolae at top of mantle (arrow) **E** external valve surface at basal pole, showing variation in areola density and alignment within the annulus, apparent discontinuity of annulus at pole, and width of basal pole relative to stem (cf. **H** at same scale) **F** internal aspect of basal pole of valve and valvocopula, showing absence of pseudoseptum (arrow) and associated structure in valvocopula **G, H** internal aspect of valve at apical pole, showing crescent of scattered areolae (arrow) and in the narrow stem, respectively **I** oblique view of basal pole with valve and valvocopula, showing increase in areola density on the valvocopula around the pole (arrow) and short row of pores on pars interior (arrowhead). Scale bars: 200 µm (**A**); 50 µm (**B**); 10 µm (**C–H**); 5 µm (**I**).

**Figure 28.**
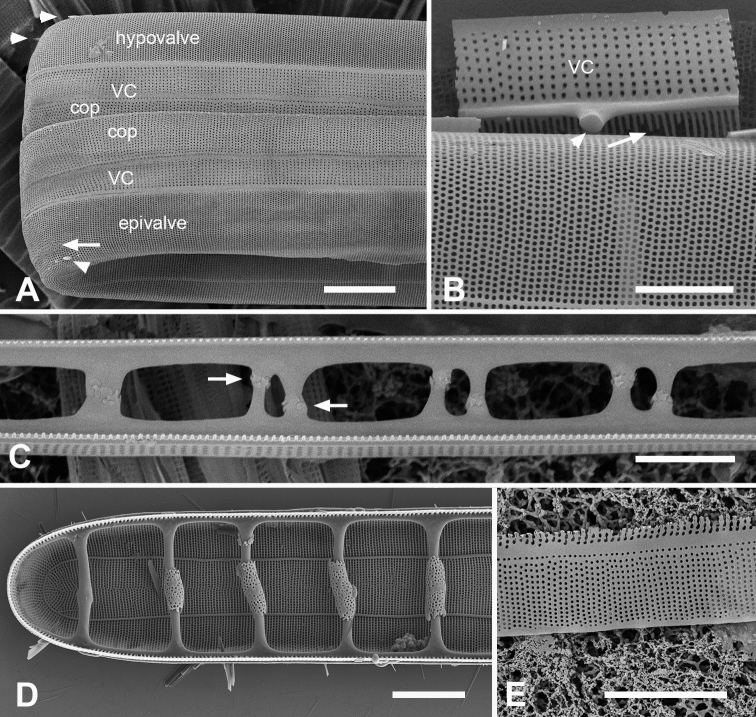
*Climacospheniaelegantissima* sp. nov., cont. GU52U-2 (**A**), GU52X-1 (**B, C**), GU44AI-5 culture (**D**), GU52AI-1 (**E**) **A** whole mount of frustule apical pole in girdle view, showing two girdle bands, patch of irregular areolae on the valve mantle (arrow), spines (arrowheads) **B** part of valvocopula broken away and showing a solid circular cross-section of the craticular bar (arrowhead) and well-developed fimbriae (arrow) **C** portion of valvocopula from stem, showing double craticular bars, ingrowths from opposite sides **D** apical pole with valvocopula showing porous bands of silica around several craticular bars **E** detail of copula showing stria density and fimbriate margin on pars interior. Scale bars: 10 µm (**A, D, E**); 5 µm (**B, C**).

Cells attached in small groups to stout, branched mucilage stalks (Fig. 26A, E), at least 110 µm wide at the base and up to 2 mm long. Frustule generally deeper than wide, especially where stem first narrows below the apical pole, ratio there about 15 µm deep to 5 µm wide (Fig. 27A). Nucleus situated between two more-widely spaced craticular bars about 120 µm from apical pole (Fig. 26A, D); plastids lenticular. Valves 750–1305 µm long, linear toward apical pole, 22–24 µm wide, then tapering to a long linear stem, 5 µm wide, expanding to 10 µm at the pole (Figs 26A–C, 27A). Stria density 27–28 in 10 µm through most of the valve but 18–21 in 10 µm near basal pole, sparser and more scattered inside annulus near basal pole (Fig. 27D, E, G). There was a crescent of scattered areolae at top of mantle at apical pole (Figs 27D, G, 28A) (noticed in *Climacosphenia* spp. by Round 1982: figs 15, 16, 25). Annulus apparently open at basal pole, sides ±parallel in the wide area, 8.3 µm apart, in the stem 2.3 µm apart, expanded to 5 µm at the basal pole (Fig. 27C–G). Two or more stout spines on external apical pole (Figs 27D, 28A), shallow transapical costae throughout except near basal pole (Fig. 27F, G). Pseudoseptum absent (Fig. 27F, G). Two girdle bands (Fig. 28A): valvocopula (Figs 26B, F, 27B, C, F, I, 28A–C) with fimbriate inner edge (Fig. 28B), stria density 19 in 10 µm (Fig. 28A, B) but increasing around foot pole, where there was also a row of pores on pars interior (Fig. 27I). Craticular bars widely spaced in wide part of the cell, with widest space corresponding to the location of the nucleus (Figs 26B, 27B); in the stem becoming much closer together (Figs 26B, 28C), often paired with the two bars apparently growing from opposite sides (Fig. 28C). In one specimen from culture, we observed porous bands of silica around several craticular bars near the apical pole (Fig. 28D). Copula fimbriate, more densely striated than the valvocopula but less than the valve, 22 in 10 µm (Fig. 28A, E). A search of abundant girdle views in whole mounts showed no sign of a pleura.

###### Holotype

**(designated here).** Specimen at 16.1 mm E, 12.8 mm S of the mark on slide 1861; deposited at Academy of Natural Sciences, Drexel University, Philadelphia, accession number ANSP GC20108. Fig. 27B.

###### Registration.

Phycobank http://phycobank.org/103246.

###### Type locality.

Guam: Apra Harbor, Scuba Beach, 13.464°N, 144.656°E, epiphytic on filamentous red algae in farmer fish territory, collection number GU52U-2, 10 May 2015. C.S. Lobban and M. Schefter leg.

###### Additional materials examined.

GU44AI-5! (culture), GU44BM-7!, GU52G-B!, GU52X-1!, GU52Y-3B!, GU52AI-1!.

###### Etymology.

Superlative of *elegans*, with reference to the slender appearance of the extremely long cells.

###### Taxonomic comments.

The width of the apical pole and especially the distance between the annular lines are narrower than in *C.elongata* (Table 5) and the stem is also narrower, though the basal pole is inflated and slightly wider than *C.elongata*. Hustedt’s (1914) drawings of *C.elongata* have the greatly extended stems that are present in our new species, but the apical portions of his cells are generally elliptical rather than linear. However, the valvocopula (Hustedt 1914, pl. 308, fig. 6) is notable in having parallel sides as far as the large space (i.e., where the nucleus would have been), so it is possible they represent *C.elegantissima*. *Climacospheniatruncata* from Borneo is also illustrated there (pl. 308, figs 1–4); it also has a very long stem but possesses an essentially triangular blade with a bluntly rounded apical pole. *C.elegantissima* is common as an epiphyte on filamentous seaweeds in Guam farmer fish territories and such habitats around the western Pacific/ IndoPacific may yield new material of *C.truncata* and other *Climacosphenia* spp.

Round (1982) showed porous bands of silica on craticular bars near the basal pole of both species only found in Hawaiian material. We saw them only once on apical bars in *C.elegantissima*. Their distribution and function are unknown.

##### ﻿ Climacosphenia scimiter

Taxon classificationPlantaeArdissonealesArdissoneaceae

A. Mann, 1925

DD178B1A-C097-527D-8799-88A008FD6425

Figs 29 , 30

###### Reference.

Mann 1925, p. 59, pl. 12, fig. 4.

###### Description from literature.

Cells tapering uniformly and strongly curved, the septa “delicate,” very narrow, “and showing at the middle either obscurely or not at all the break or sutural division common to specimens of this genus.” Length 414 µm, width at apical pole 28 µm, stria density not specified.

###### Materials examined.

GUAM: GU52U-2!, GU52AH!, GU44Z-15!. F.S.M.: Chuuk: TK28!.

###### Observations.

**Figure 29.**
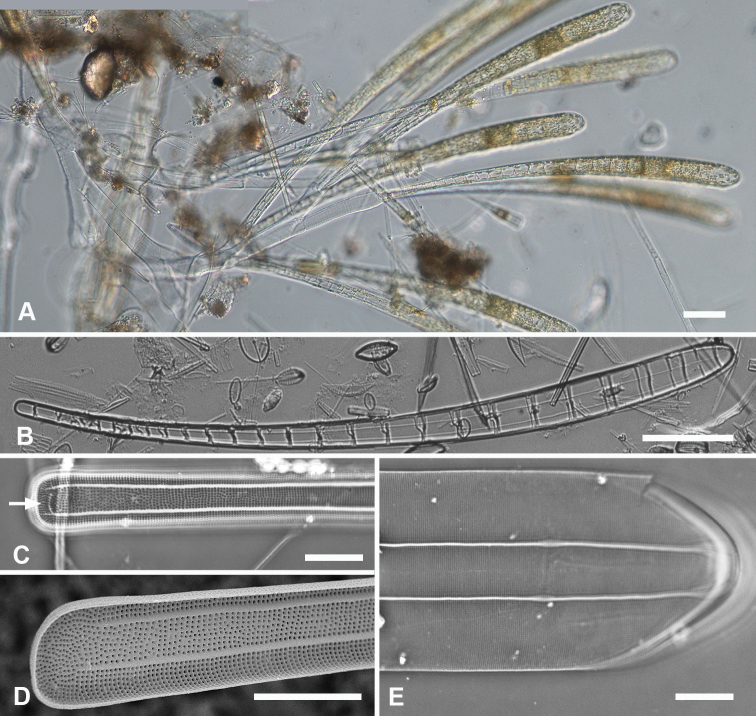
*Climacospheniascimiter*, all from GU52X-1 **A** colony of living cells on branched mucilage stalk **B** valve and valvocopula of cleaned material, LM-DIC, showing consistently narrow craticular bars **C, E** valve in phase contrast LM, showing more strongly silicified basal pole in **C** and weakly silicified apical portion in **E**, along with difference in stria density; note appearance of annulus at basal pole (arrow, **C**) **D** basal pole in SEM, oblique view, showing shallow mantle and very weak costae and apparently discontinuous annulus. Scale bars: 50 µm (**A, B**); 10 µm (**C–E**).

**Figure 30.**
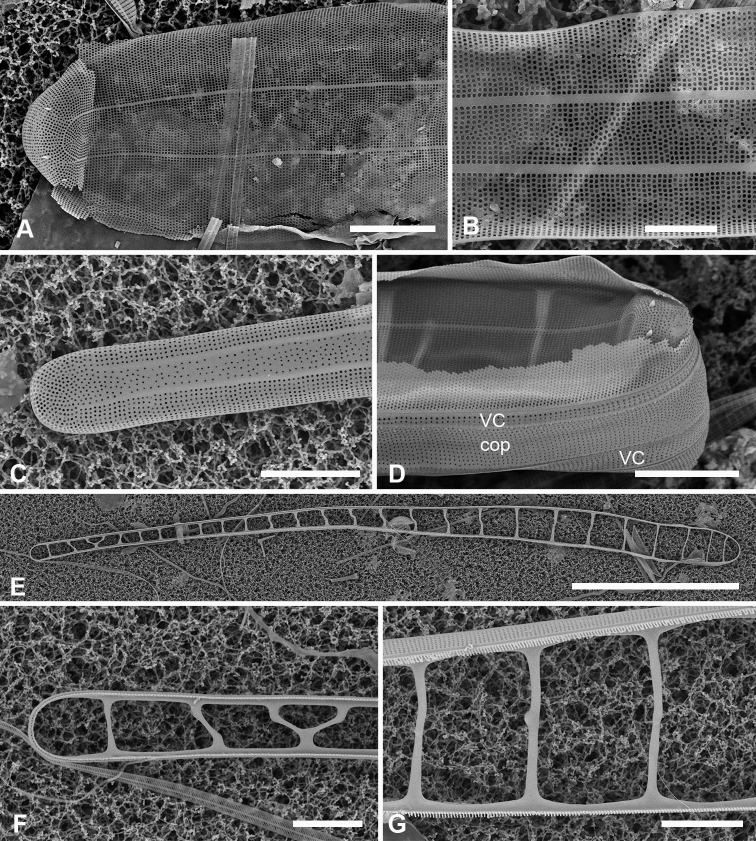
*Climacospheniascimiter*, cont. All GU52X-1 except **D**, GU52U-2 **A** apical pole of valve, exterior view, showing stria density and spines **B** detail of valve internal aspect, showing absence of costae **C** detail of external valve face at basal pole, showing more scattered areolae inside annulus **D** whole mount of frustule, apical pole, showing spines and girdle bands **E–G** valvocopulae showing consistently seamless craticular bars. Scale bars: 100 µm (**E**); 10 µm (**A, C, D, F, G**); 5 µm (**B**).

Specimens fitting this description have been found in Guam and Chuuk, often together with *C.elegantissima*. Colonies on branched mucilaginous stalks, the nucleus near the apical pole between two of the widely spaced craticular bars (Fig. 29A). Length 390–612 µm, maximum width 25–26 µm near apical pole, 8–10 µm across basal pole (Fig. 29A, B). Stria densities outside the annulus 21 in 10 µm in lower part, 29 in 10 µm at apical pole; striae within annulus at basal pole about 16 in 10 µm, areolae more, or less aligned (Fig. 29C–E vs Fig. 30A). Maximum distance across annulus 10 µm (Figs 29B, D, 30A). While annulus appears discontinuous in SEM, phase contrast suggests continuity (Fig. 29C). Valves delicate except at basal pole, apical striae hard to resolve in LM even with phase contrast (Fig. 29C, E), costae not developed on either vimines or annulus, except near basal pole (Fig. 29D). Two spines on apical pole of valve (Fig. 30A, D). Two girdle bands present, both broad and with regular striae, but at different densities, valvocopula 20 in 10 µm, copula 24 in 10 µm (Fig. 30D). Valvocopulae fimbriate with narrow, widely spaced craticular bars, unions all seamless (Fig. 30E–G).

###### Taxonomic comments.

Observations are completely in accord with Mann’s (1925) description of specimens from the Philippines (Mann noted that data on the locations of the dredge samples were lost). Given also that this species was discovered not far from Micronesia, we are confident in the identification. We cannot accept that this is merely a curved *C.moniligera*, as suggested by Round (1982), because of the great difference in septum characters and differences in stria densities. Whether there exist straight versions of this species, or curved *C.moniligera* populations, remain open questions.

##### ﻿ Climacosphenia soulonalis

Taxon classificationPlantaeArdissonealesArdissoneaceae

Lobban & Joon S. Park sp. nov.

1DAD25DA-B1F5-564C-A53F-F986D8A6459A

Fig. 31

###### Diagnosis.

Differing from *C.moniligera* in apical stria density and delicate craticular bars throughout, seamless except for complex unions in the most basal 1–4 bars and from *C.scimiter* in straight outline, presence of complex unions, and cell width.

###### Description.

**Figure 31.**
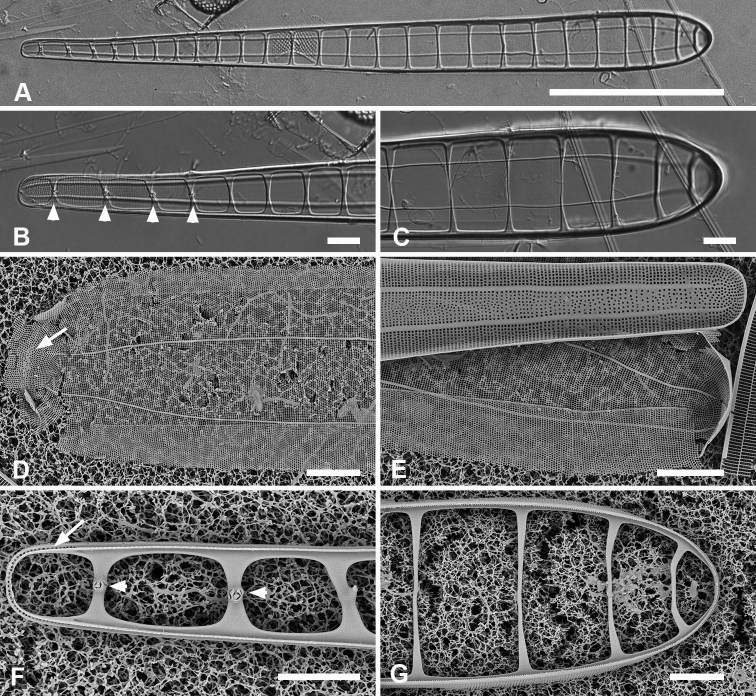
*Climacospheniasoulonalis* sp. nov. All TK4 **A–C** holotype valve with valvocopula, entire, basal pole and apical pole, respectively, showing seamless craticular bars except near basal pole; arrowheads on **B** show complex unions **D** external valve apical pole showing irregular patch of areolae (arrow), spines, and narrowing of the annulus at apical pole **E** internal aspects of basal and apical poles, showing lack of costae (contrast costae in the piece of *Synedrospheniacrystallina* to the right) **F, G** valvocopula with two complex unions at the basal pole (arrowheads, **F**) and showing the row of pores on the pars interior around the basal pole (arrow). Scale bars: 100 µm (**A**); 10 µm (**B–G**).

Valves straight, gradually tapering from apical pole almost to basal pole (Fig. 31A–C), only a short basal part with parallel sides. Length 390–530 µm, width 29–35 µm across apical pole (measured on valvocopula; total valve width including mantles 50 µm), 10–12 µm across basal pole at last craticular bar (Fig. 31A–G). Annulus narrower at apical pole (Fig. 31C–E), widest 15 µm, steadily tapering to 4 µm, apparently open at basal pole (Fig. 31E). Stria densities 18–20 in 10 µm in lower part of the periphery, 28–29 in 10 µm in periphery at apical pole, about 16 in 10 µm in middle part above basal pole (Fig. 31E). Spines present on apical pole and a small patch of irregular areolae (Fig. 31D). Costae apparently poorly developed. Valvocopula fimbriate with narrow, widely spaced craticular bars that have seamless unions except for the most basal 1–4 (Fig. 31B, F); a row of pores on pars interior just around basal pole (Fig. 31F). Copula not positively identified.

###### Holotype

**(designated here).** Specimen at 17.8 E, 10.9 mm S of the mark on slide 153; deposited at ANSP, accession number GC20109. Fig. 31A–C.

###### Registration.

Phycobank http://phycobank.org/103247.

###### Type locality.

Federated States of Micronesia: Chuuk: Northeast Pass into Chuuk Lagoon, 7.514°N, 151.967°E, on algal turf from farmer fish territory, lee side of Moch I., collection number TK4!, 30 May 1991. C.S. Lobban and M. Schefter leg.

###### Additional materials examined.

Chuuk: TK28!, exposed reef, ca. 6 m deep, southeast corner of Northeast Passage.

###### Etymology.

Belonging to Sou-lon, “the Chuukese god of ocean depths and storms, equivalent, perhaps to Neptune” (W.M. Peck 1992, Chuukese Testament. Storyboard: A Journal of Pacific Imagery 2: 44).

###### Taxonomic comments.

Valves resemble *C.moniligera* in size and stria densities (Table 5) but differ in the valve shape and the narrow and mostly seamless craticular bars are unlike the massive septum with complex unions in the bars reported for *C.moniligera* (Peragallo and Peragallo 1897–1908; Hustedt 1931–1959; Round 1982).

### ﻿Systematics

#### Morphological cladistics

**Figure 32.**
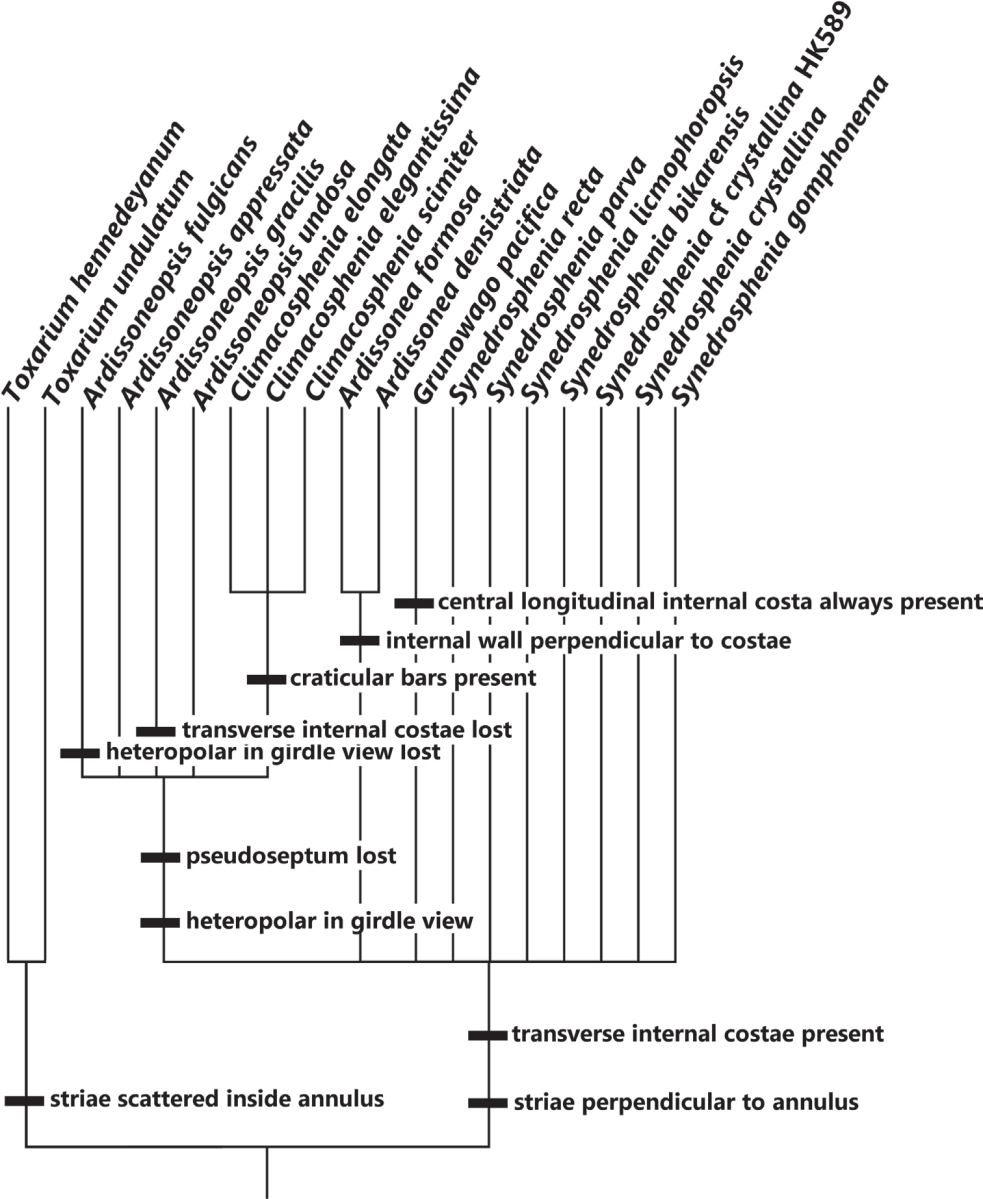
Strict consensus tree summarizing the 18 most-parsimonious trees resolved from the cladistic analysis of the morphological dataset in Suppl. material 2: Table S2. Several diagnostic character states shown on the diagram. Length = 34 steps; Consistency Index = 0.55; Retention Index = 0.75.

The cladistic analysis of the character set (Suppl. material 2: Table S2) resolved 18 equally parsimonious trees, which we have summarized as a strict consensus tree (Fig. 32). The genera with striae perpendicular to the bifacial annulus across the entirety of the valve (*Ardissonea*, *Synedrosphenia*, *Ardissoneopsis*, *Climacosphenia* and *Grunowago*) formed a monophyletic group (*Toxariumhennedeyanum* was used as the outgroup). Three genera appear to have morphological synapomorphies: *Climacosphenia* (craticular bars on the valvocopula), *Ardissonea* (the internal wall) and *Grunowago* (the central longitudinal costa). Morphological synapomorphies for *Ardissoneopsis* and *Synedrosphenia* are less clear but each genus has a unique combination of the main characters (Table 6). The species of *Ardissoneopsis* form a polytomy with *Climacosphenia*, sharing the heteropolar shape of the frustule in girdle view (though this character is lost in *A.fulgicans*). The taxa in *Synedrosphenia* have a range of character states, and the only characters shared by all the species (internal transverse costae, pseudosepta, apical notch on the valvocopula and fimbriate girdle bands) are also shared by species in other genera.

**Table 6.** Summary of Ardissoneaceae generic characters.

Genus	Valve wall	Polar architecture^†^	Striae on valve face	Central long costa	Craticular bars
*Ardissonea*	Double	Complex	Linear	Absent	Absent
*Synedrosphenia*	Single	Complex	Linear	Absent	Absent
*Ardissoneopsis*	Single	Simple	Linear	Absent	Absent
*Grunowago*	Single	Simple	Linear	Present	Absent
*Toxarium*	Single	Complex	Scattered	Absent	Absent
*Climacosphenia*	Single	Simple	Linear	Absent	Present

† Complex refers to the presence of a pseudoseptum on the valve poles + corresponding shelf on valvocopula poles + long fimbriae on the copula poles.

#### Phylogenetics

**Figure 33.**
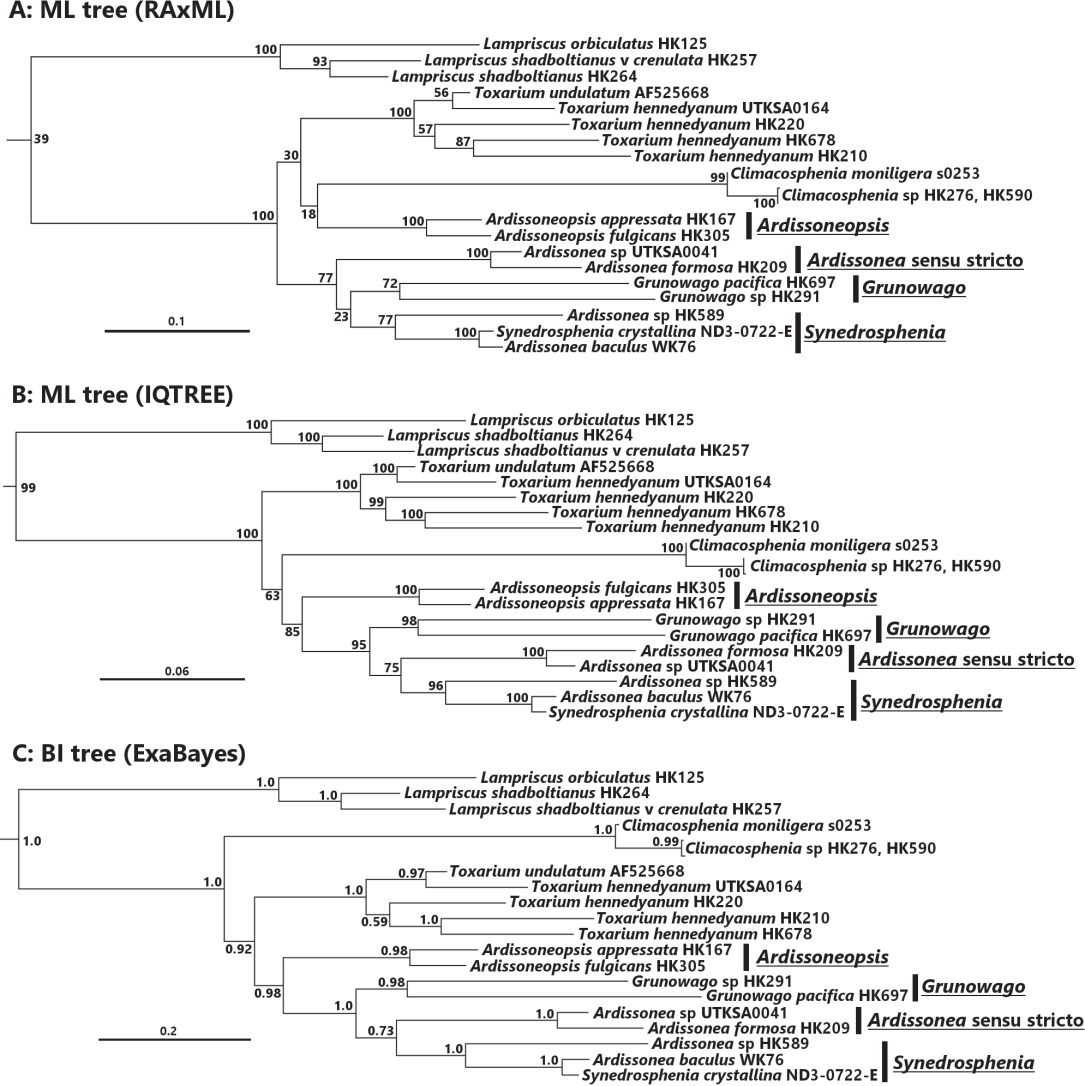
Phylogenetic trees resulting from three analytical methods of the 3-gene DNA sequence dataset assembled for this manuscript. Only the Ardissoneaceae and their sister group in the molecular phylogeny, *Lampriscus*, is shown here—the complete trees can be found in Suppl. materials 5–7: Figs S3–S5 **A** Maximum Likelihood phylogeny resolved by RAxML, where values at nodes are bootstrap support values **B** Maximum Likelihood phylogeny resolved by IQTREE, where values at nodes are bootstrap support values **C** Bayesian Inference phylogeny resolved by ExaBayes, where values at nodes are posterior probability values. Strain taxonomy (name followed by DNA voucher ID or strain ID) follows currently accepted classification, with proposed genera based on morphology shown to the right of strain ID.

Under all three analytical programs, a well-supported clade (bootstrap support [bs] = 100%, posterior probability [pp] = 1.0) contained all the sequenced *Climacosphenia*, *Toxarium*, *Grunowago* and *Ardissonea* sensu lato strains (Fig. 33). The *Ardissonea* sensu lato strains sequenced here represent the *Ardissonea* sensu stricto morphology (*Ardissonea* sp. UTKSA0041 and *Ardissoneaformosa* HK209), *Ardissoneopsis* morphology (*Ardissoneopsisfulgicans* HK305 and *Ardissoneopsisappressata* HK167), the *Synedrosphenia* morphology (*Ardissonea* sp. HK589, *Ardissoneabaculus* WK76 and *Ardissoneacrystallina* ND3-0722-E) and the *Grunowago* morphology (*Synedrabacillaris* HK291 and HK697). All morphotypes were monophyletic (bs > 72%, pp > 0.98), though support was lowest under the RAxML analysis.

The genera *Climacosphenia* and *Toxarium* were also monophyletic in all three analyses (bs > 99%, pp = 1.0), though their position relative to other taxa changed between the analyses. The *Climacosphenia* sequences were sister to the rest of the clade in the ExaBayes analysis (pp = 1.0). In the Maximum Likelihood analyses, *Climacosphenia* was either sister to the *Ardissonea* clades (IQTREE, bs = 63%) or sister to the *Ardissoneopsis*-morphotype strains, but with extremely low support (RAxML, bs = 18%). Strains with the *Ardissonea* sensu stricto morphology were either sister to the *Synedrosphenia* morph strains (IQTREE bs = 75%, ExaBayes pp = 0.73) or sister to a clade containing the *Grunowago* and *Synedrosphenia* morphs (RAxML bs = 77%). The *Ardissoneopsis* morph clade was sister to the rest of the *Ardissonea* sensu lato clades in the IQTREE and ExaBayes analyses (bs = 85%, pp = 0.98, respectively), but in a clade with *Climacosphenia* which was sister to *Toxarium* in the RAxML analysis (bs = 30%).

## ﻿Discussion

The results support a conclusion that there is one monophyletic group containing six genera (Table 6), i.e., *Ardissonea* sensu stricto with double walls and pseudosepta; (2) *Synedrosphenia*, including *Ardissoneacrystallina* and *A.fulgens*, with single walls and pseudosepta; (3) *Ardissoneopsis* gen. nov., including *Synedraundosa* Grunow, with single walls but without pseudosepta; (4) *Grunowago* gen. nov. including *Synedrabacillaris*, with a central costa; (5) *Toxarium* with scattered areolae on the valve face; and (6) *Climacosphenia* with craticular bars on the valvocopula. The following paragraphs further discuss the new and emended genera, before considering the limitations of the study and what we can conclude about the Family/Ordinal level.

There is a clear distinction within *Ardissonea* sensu lato, between species with a double wall and those with a single wall, as noted by Round et al. (1990), i.e. the double wall is a synapomorphy for *Ardissonea* sensu stricto. Even without *Ardissonea* s.s., single-walled taxa under the name *Ardissonea* were not monophyletic and resolved into two clades. In Micronesia, several species differ subtly but perhaps significantly from the known taxa, and there are species sufficiently different to be recognized as undescribed even without sequencing. The two clades of single-walled *Ardissonea* spp. can be separated on the structure of the poles, one group, including *Synedrospheniagomphonema* and therefore taking that genus name, with complex polar architecture (also seen in *Ardissonea* s.s.) including a narrow, often asymmetrical extension (pseudoseptum) of the rim of the valve at the apical pole, a corresponding folding of the valvocopula to form a shelf with a groove or notch, often overlapped internally by very long fimbriae of the copula. The other group has simple polar architecture, commonly seen in diatoms, with a straight edge to the mantle and the pars interior of the valvocopula; we propose *Ardissoneopsis* for this group, including three new species, and typified by Grunow’s obscure species *Synedraundosa*. *Toxarium* is within a clade with the other genera discussed here in two of three molecular analyses and does not clearly separate in morphological cladistics, suggesting that all belong in one family.

*Synedrosphenia* was kept separate from *Synedra* by Hustedt (1931–1959), even though he rejected *Ardissonea* as a genus. His argument was that, despite the many similarities, the heteropolar shape of the valve was more important, and he cited pairs of genera, e.g., *Gomphonema* and *Navicula*, in which heteropolar species were separated from isopolar. *Synedrosphenia* was said by Round et al. (1990) to be in “urgent need of re-investigation,” but, based on the ultrastructure and cell shape, they recognized a relationship with *Climacosphenia*. With more recent EM studies on single-walled *Ardissonea* species (Poulin et al. 1987; Kaczmarska et al. 2020), especially the recognition of complex polar architecture in *Ardissoneafulgens* type material (Kanjer et al. 2021), and our observations on *Synedrosphenia* from Micronesia and Grunow’s historical material from Honduras, it is now clear that the ultrastructural similarities must outweigh the heteropolarity. (We reserve judgement on specimens identified in the literature as A.fulgensvar.gigantea for reasons detailed in Results.) As seen in our new species, the shape presents more of a gradient and strongly suggests that all the single-wall *Ardissonea* and new species with a pseudoseptum should be placed in *Synedrosphenia* rather than erecting a new genus. A dearth of sequenced taxa means that this conclusion is not yet supported by molecular data.

The new genus *Ardissoneopsis* is necessary to accommodate *Ardissonea*-like species with single walls but simple polar architecture. These include not only two species in Micronesia that we had previously identified with Ardissoneafulgens and its var. gigantea, but also Grunow’s *Synedraundosa*, which we were able to observe for the first time in SEM, and a new species. While it is better to have a positive synapomorphy for a genus, the complexity of the apical structure of valve and valvocopula in some other genera in this group is a strong character, comparable to the presence of an inner wall layer, and warrants separating those that have it from those that do not. This genus is presently small but we anticipate it will grow as Ardissoneaceae are more fully explored in other regions.

The new genus *Grunowago* is required to accommodate a new species and *Synedrabacillaris*. The latter cannot remain in *Synedra*, which, although defined by Round et al. (1990) as a freshwater genus and now clarified as a small *marine* genus, with *Catacombas* as a junior synonym (Williams and Karthick 2021), has a double wall, rimoportulae, and polar pore fields. Valve structure in *Grunowago* has some important differences that seem to set it apart from the Ardissoneaceae, including a strong central costa and lack of lateral costae that implies bilateral growth rather than annular growth. Nevertheless, sequence results show that it is part of this Family. Not all the other Ardissoneaceae have evident annular rings, however. As noted above, the ring is not visible in *Synedrospheniabikarensis*; this species has not been sequenced yet. Our hypothesis to explain this is that there is annular growth in these species, since that is the defining character of the group. Regardless of its eventual systematic position, this genus helps resolve *Synedra*.

Our study was largely restricted to observations on materials from Western Pacific Islands, those from Guam usually examined fresh, others only after acid cleaning. Having stubs with remnants from Grunow’s Honduras materials, we made use of them, but the large specimens were very broken up and we had much more success with species with distinctive shapes (*Synedraundosa*, *Toxariumundulatum*, Figs 17, 24) than with the linear species with several similar species that we could not identify. We managed to identify putative Synedracrystallinavar.bacillaris (Fig. 19) but it was hard to be sure that the specimens did not have a double wall when *Ardissonea* was also present. We restricted descriptions of new species to those that could be confidently differentiated from known species, leaving others, such as the two *Toxarium* spp. with the widely used names as working hypotheses. The other major limitation in this study is low taxon sampling for sequencing. Low taxon sampling may not change quickly, as Medlin et al. (2008) commented that *Ardissonea* and *Climacosphenia* are difficult to culture, and that has been our experience as well. Many of the new species were found only after acid cleaning of samples that had been preserved in the herbarium and therefore could provide no information on organic components, including DNA. Low taxon sampling is also the cause of the apparently sister relationship with the centric diatom *Lampriscus* (Fig. 33); it is closest but not close.

Despite the limitations of the study, we can draw some conclusions about the Families and Order. Molecular and morphological evidence support a hypothesis that the six genera in our flora are in one monophyletic family but there is no known common ancestor, from which to derive a synapomorphy for the Family, which now appears to stand alone in an Order with no other Mediophyceae nearby. In the past they were placed in several Families and usually more than one Order; the most recent summary of diatom taxonomy has them in four families under one Order, Toxariales (Cox 2015). Round et al. (1990) described *Ardissonea* based on the type species, *A.robusta*, without reference to the single walled forms that Poulin et al. (1986) had used as the basis for their emendation. However, they also defined a Family and an Order with one and the same paragraph, which, because it was based entirely on the characters of *A.robusta*, even excludes the single-walled species from these higher taxa. Cox (2015) partially resolved these issues by eliminating the Ardissoneales and placing the family in Toxariales. However, her description of Ardissoneaceae mirrors Round’s and she lists only one genus—*Ardissonea*, with only 2 species (presumably *A.robusta* and *A.formosa*), still leaving the single-walled taxa in limbo. Moreover, she also follows Round et al. (1990) in placing *Synedrosphenia* in Climacospheniaceae, which is defined as having valvocopulae with septa that meet in the middle, true only for *Climacosphenia*. We therefore need to emend the description of Ardissoneaceae. First, *Ardissonea*, *Ardissoneopsis* and *Synedrosphenia* (Groups 1–3 as defined above) clearly all belong in Ardissoneaceae as presently conceived. Second, there seems to be little justification for maintaining separate Family+Order for each of *Toxarium* and *Climacosphenia*, given the similarities to the Ardissoneaceae. *Toxarium* has characters of both *Ardissoneopsis* (weakly developed costae) and *Synedrosphenia* (pseudoseptum + valvocopular notch) and is distinguished only by the scattered areolae in the wider areas (when there are any inside the annulus). While the craticular bars of *Climacosphenia* are its defining character, unique within the Order, we do not see this as warranting a separate family, especially considering the distribution of such valvocopulae within the biraphid genus *Climaconeis*, where craticular bars distinguish only 2 of 20 species (Lobban 2021a). We therefore propose to transfer *Toxarium* and *Climacosphenia* to the Ardissoneaceae and to restore that family to the Ardissoneales, emended.

Much work remains to be done even in our region to tease out the biodiversity of these genera, and that work depends on progress in other regions and especially on type materials. Some species, e.g., *Ardissoneaformosa* and *A.pulcherrima*, were described from the East Indies, others from Atlantic/Caribbean waters, e.g. *A.robusta* from Kattagat Strait, Europe, and several species from Honduras. In many cases precise type locations are unknown – Hantzsch received samples labeled only from the East Indies Archipelago; Lindig did not inform Grunow whether his Honduras samples were from its east or west coast. It is tempting but counterproductive to assume that everything is everywhere. Moreover, Williams (2011) and Williams and Kociolek (2017), among others, have found evidence of regional endemicity in freshwater diatoms, and our studies of *Licmophora* (Lobban and Santos in review) indicate the same in that marine genus. Williams (2011) even proposed that we should start from the assumption that everything is endemic, rather than that everything is everywhere. At least we should not assume that everything is everywhere, nor that there is anywhere that the full biodiversity of Ardissoneaceae has been discovered. Likely more species will be resolved with further study both in our region and in other places where these large but understudied species occur.

### ﻿Taxonomic revisions

#### ﻿ Ardissoneales

Taxon classificationPlantaeArdissonealesArdissoneaceae

Round 1990, emend. Lobban & Ashworth

366CBA73-2824-592F-864D-B3CD6AA65F76

##### Description.

Highly elongate valves with development from a bifacial annulus (not always visible) and lacking rimoportulae and apical pore fields (ocelli/ocellulimbi). Frustules often morphologically heteropolar in valve view, girdle view, or both, attached to substrata by mucilage stalks. Valves with simple areolae in uniseriate striae, internal transapical costae more or less developed. Valvocopula and copula closed, fimbriate internal margins. Pleura (when present) narrow, fimbriate inner margin, with larger apical caps.

#### ﻿ Ardissoneaceae

Taxon classificationPlantaeArdissonealesArdissoneaceae

Round 1990, emend. Lobban & Ashworth

3A632EE1-6924-58EF-919E-54CA559D1BD3

##### Remark.

With the characters of the Order.

##### Included genera.

*Ardissonea* De Notaris, sensu stricto

*Ardissoneopsis* Lobban & Ashworth, gen. nov.

*Synedrosphenia* (Peragallo) Azpeitia

*Grunowago* Lobban & Ashworth, gen. nov.

*Toxarium* J.W. Bailey

*Climacosphenia* Ehrenberg

#### ﻿ Ardissonea

Taxon classificationPlantaeArdissonealesArdissoneaceae

De Notaris 1871, emend. Poulin et al. 1986, emend. Lobban & Ashworth

FA6E81D6-7C4B-505B-BD08-C2F87B174994

##### Description.

Valves elongate, linear to lanceolate, alveolate except at the apical pole, owing to an internal silica plate and longitudinal costae under the annulus and sometimes under the midline. Inner plate opening through several large foramina at the poles and each alveolus opening by a small foramen into the cell interior.

##### Included species.

*Ardissonearobusta* (Ralfs) De Notaris (generitype).

*Ardissoneadensistriata* Lobban sp. nov.

*Ardissoneaformosa* (Hantzsch) Grunow ex De Toni.

*Ardissoneapulcherrima* (Hantzsch) Grunow ex De Toni.

#### ﻿ Synedrosphenia

Taxon classificationPlantaeArdissonealesArdissoneaceae

(H. Peragallo in H. Peragallo and M. Peragallo 1897–1908) Azpeitia 1911, emend. Lobban & Ashworth

E5A3F781-4AB5-50AB-952E-8FF45C2DDDE6

Synedrosphenia gomphonema (Janisch and Rabenhorst) Hustedt, 1932. Synonym.
≡ Synedragomphonema Janisch & Rabenhorst, 1863 Beiträge zur näheren Kenntniss und Verbreitung der Algen. Heft I, p. 13, pl. 2: fig. 6. Synonym.

##### Lectotype species here designated.

*Sceptroneiscuneata* Grunow,1877, Monthly Micros. J. 18: 169, pl. 194, fig. 3 ≡ *Synedracuneata* Grunow, 1866, Hedwigia 1: 5.

##### Registration of lectotype.

Phycobank http://phycobank.org/103264.

##### Description.

Valves elongate, linear or tapered from a broader apical pole. Wall a single layer usually with internal costae on the virgae, and a pseudoseptum on the rim of the valve at each pole, forming a complex structure with the valvocopula. Midline absent or indistinct (not subtended by a longitudinal costa), annulus usually evident, though sometimes coinciding with the valve-mantle junction.

##### Included species.

*Synedrospheniagomphonema* (Janisch and Rabenhorst) Hustedt (= *Sceptroneiscuneata* Grunow; type species).

*Synedrospheniabaculum* (Gregory) Lobban & Ashworth, comb. nov.

*Synedrospheniabikarensis* sp. nov.

*Synedrospheniacrystallina* (C. Agardh) Lobban & Ashworth comb. nov.

*Synedrospheniafulgens* (Greville) Lobban & Ashworth comb. nov.

*Synedrosphenialicmophoropsis* sp. nov.

*Synedrospheniaparva* sp. nov.

#### ﻿ Synedrosphenia crystallina

Taxon classificationPlantaeArdissonealesArdissoneaceae

(C.Agardh) Lobban & Ashworth comb. nov.

AE594637-B577-50E0-B6B1-FAF191F4460A

Diatoma crystallinum C. Agardh 1824, Systema Algarum, p. 3. Basionym.
Synedra crystallina (C. Agardh) Kützing, 1844, Die Kieselschaligen Bacillarien oder Diatomeen, p. 69, pl. 16: fig. 1. Synonym.
Ardissonea crystallina (C. Agardh) Grunow ex De Toni 1892. Synonym.

##### Registration.

Phycobank http://phycobank.org/103250.

#### ﻿ Synedrosphenia baculum

Taxon classificationPlantaeArdissonealesArdissoneaceae

(Gregory) Lobban & Ashworth comb. nov.

BDBE7FBA-D7EB-5F81-BB10-5936CC1A16DD

Synedra baculus Gregory, Trans. Micr. Soc. London, 5: p. 83, pl. 1, fig. 54, 1857. Based on GenBank sequence wk76 (Wiebe Kooistra). Basionym.

##### Registration.

Phycobank http://phycobank.org/103252.

##### Comment.

Since *baculum* (baton, rod) is used as a noun in apposition, it should retain its neuter ending, even though Gregory (1857) wrote it *Baculus*. This is an orthographical change that does not require any formal action under ICN rules, Art. 61 (Turland et al. 2018).

In addition, *A.dalmatica* (Kützing) De Toni, 1892, shown by Álvarez-Blanco and Blanco (2014) to have a pseudoseptum and single wall, clearly belongs in *Synedrosphenia*. We have not made a new combination here, pending further investigation into its relationship to *A.crystallina*. Similarly, we do not have enough information to decide where to place *Ardissonea brockmannii ‘brockmanni*’ Hustedt, 1932 (Hustedt 1931–1959, p. 228, fig. 716).

#### ﻿ Synedrosphenia fulgens

Taxon classificationPlantaeArdissonealesArdissoneaceae

(Greville) Lobban & Ashworth comb. nov.

00EAAEC3-AE24-51B9-8444-92E92BB30752

Exilaria fulgens Greville, Scottish Cryptogamic Flora, Vol. 5: pl. 291, 1827. Basionym.
Synedra fulgens (Greville) W.Smith, A Synopsis of British Diatomaceae, vol. 1, p. 74, pl. 12, fig. 103, 1853. Synonym.
Ardissonea fulgens (Greville) Grunow ex De Toni, Sylloge, p. 674, 1892. Synonym.
Ardissonea fulgens (Greville) Kanjer, Kusber & Van de Vijver 2021, Notulae Algarum 215: 1–6, figs 1–16. Synonym.

##### Registration.

Phycobank http://phycobank.org/103259.

##### Comment.

We do not include Ardissoneafulgensvar.gigantea (Lobarzewsky) Rabenhorst in this transfer because of the uncertainty about its morphology.

#### ﻿ Ardissoneopsis

Taxon classificationPlantaeArdissonealesArdissoneaceae

Lobban & Ashworth gen. nov.

809BFF49-6017-56AE-8861-DDC35F8AD171

##### Description.

Valves single walled, isopolar or weakly heteropolar, simple polar architecture on valve and valvocopula.

##### Type species.

*Ardissoneopsisundosa* (Grunow) Lobban & Ashworth comb. nov.

##### Etymology.

*Ardissonea* [named for Italian phycologist Francesco Ardissone (1837–1910)] + -*opsis*, L. similar to.

##### Included species.

*Ardissoneopsisfulgicans* Lobban & Ashworth, sp. nov.

*Ardissoneopsisappresssata* Lobban & Ashworth, sp. nov.

*Ardissoneopsisgracilis* Lobban sp. nov.

*Ardissoneopsisundosa* (Grunow) Lobban & Ashworth comb. nov.

##### Registration.

Phycobank http://phycobank.org/103240.

#### ﻿ Ardissoneopsis undosa

Taxon classificationPlantaeArdissonealesArdissoneaceae

(Grunow) Lobban & Ashworth comb. nov.

AFA9BCB7-AF87-558C-8CFC-DC890C8D8500

Synedra undosa Grunow 1867 Hedwigia 6: 4; and 1877 Monthly Microscopical Journal 18: p. 167, pl. 193, fig. 8a–c. Basionym.
Toxarium undosum (Grunow) De Toni 1892, Sylloge, p. 677. Synonym.

##### Registration.

Phycobank http://phycobank.org/103260.

#### ﻿ Grunowago

Taxon classificationPlantaeArdissonealesArdissoneaceae

Lobban & Ashworth gen. nov.

90989100-D240-5292-A106-4FF16855E501

##### Description.

Valves linear to lanceolate, isopolar; uniseriate striae, virgae thickened internally and a prominent longitudinal costa under the midline. Annulus not apparent even on the valve–mantle junction. No pseudoseptum but continuous rim on valve border.

##### Type species.

*Grunowagobacillaris* (Grunow) Lobban & Ashworth.

##### Etymology.

Grunow + -*ago* (from *agere*, to move, perform, achieve, etc.; see Stearn 1973: 293) Named in recognition of the body of work of Albert Grunow (1826–1914), which included the original recognition of the type species in his study of a Honduras sample. The compound noun is feminine.

##### Included species.

*Grunowagobacillaris* (Grunow) Lobban & Ashworth, comb. nov.

*Grunowagopacifica* sp. nov.

##### Registration.

Phycobank http://phycobank.org/103244.

#### ﻿ Grunowago bacillaris

Taxon classificationPlantaeArdissonealesArdissoneaceae

(Grunow) Lobban & Ashworth comb. nov.

C5530139-4069-53CB-B293-AE157EAA81F9

Synedra crystallina var. bacillaris Grunow 1877, Monthly Microscopical Journal 18: 167, pl. 193, fig. 12. [Grunow gives the authorship there as Grunow, rather than sp. nov., implying that he had already described it, but it is not in his original work on the Honduras material (Grunow 1867).]. Basionym.
Ardissonea crystallina var. bacillaris (Grunow) Grunow in Cleve and Grunow 1880, Kongliga Svenska Vetenskaps-Akademiens Handlingar 17(2): 108. Synonym.
Synedra superba Peragallo 1900, Diatomees Marine de France, pl. 79, fig. 7 (Peragallo and Peragallo 1897–1908). Synonym.
Synedra bacillaris (Grunow) Hustedt 1931–1959, Die Kieselalgen 2(2): 230, fig. 718. Synonym.

##### Registration.

Phycobank http://phycobank.org/103261.

## ﻿Funding

Terance Camacho was a 2019 intern supported by the NSF-funded U. Guam Louis Stokes Alliances for Minority Participation (LSAMP) Islands of Opportunity grant and worked on the early stages of imaging and analysis. The new SEM and later stages of imaging and write-up of this project were supported by the National Science Foundation award No. OIA-1946352, RII Track-1: Guam Ecosystems Collaboratorium for Corals and Oceans (GECCO); any opinions, findings, and conclusions or recommendations expressed in this material are those of the author(s) and do not necessarily reflect the views of the National Science Foundation. Financial support for SEM and sequencing efforts at the University of Texas at Austin was provided by the Harold C. and Mary D. Bold Regents Professorship of Cryptogamic Botany (Phycology) to ECT.

## Supplementary Material

XML Treatment for Ardissonea formosa

XML Treatment for Ardissonea densistriata

XML Treatment for Ardissonea crystallina

XML Treatment for Synedrosphenia gomphonema

XML Treatment for Synedrosphenia bikarensis

XML Treatment for Synedrosphenia licmophoropsis

XML Treatment for Synedrosphenia parva

XML Treatment for Synedrosphenia recta

XML Treatment for Ardissonea fulgens

XML Treatment for Ardissoneopsis fulgicans

XML Treatment for Ardissoneopsis appressata

XML Treatment for Synedra undosa

XML Treatment for Ardissoneopsis gracilis

XML Treatment for Synedra bacillaris

XML Treatment for Grunowago pacifica

XML Treatment for Toxarium hennedyanum

XML Treatment for Toxarium undulatum

XML Treatment for Climacosphenia elongata

XML Treatment for Climacosphenia elegantissima

XML Treatment for Climacosphenia scimiter

XML Treatment for Climacosphenia soulonalis

XML Treatment for Ardissoneales

XML Treatment for Ardissoneaceae

XML Treatment for Ardissonea

XML Treatment for Synedrosphenia

XML Treatment for Synedrosphenia crystallina

XML Treatment for Synedrosphenia baculum

XML Treatment for Synedrosphenia fulgens

XML Treatment for Ardissoneopsis

XML Treatment for Ardissoneopsis undosa

XML Treatment for Grunowago

XML Treatment for Grunowago bacillaris

## Supplementary materials

Supplementary material 1 Table S1. Metadata for strains used in the molecular phylogenetic analysis, including strain extraction voucher ID and GenBank accession numbers Data type Table of GenBank numbers (word file) Explanation note **Table S1.** Metadata for strains used in the molecular phylogenetic analysis, including strain extraction voucher ID and GenBank accession numbers. Collection site for the source of the original strain isolation is also included, where known. Extraction voucher ID corresponds to the culture material extracted for the DNA used here and references dried frustules left over from the DNA extraction as well as photomicrographs of the cells, available from authors MPA or ECT. Taxa are listed alphabetically. https://binary.pensoft.net/file/746750This dataset is made available under the Open Database License (http://opendatacommons.org/licenses/odbl/1.0/). The Open Database License (ODbL) is a license agreement intended to allow users to freely share, modify, and use this Dataset while maintaining this same freedom for others, provided that the original source and author(s) are credited.Matt P. Ashworth

Supplementary material 2 Table S2. Morphological characters and character states evaluated for the morphological cladistic analysis Data type Morphological character matrix (excel file) Explanation note **Table S2.** Morphological characters and character states evaluated for the morphological cladistic analysis. Taxa are arranged by the taxonomy proposed in this manuscript, but are labeled by their basionyms. Character state “U” indicates where the state is unknown for a taxon. Characters “pseudoseptum” and “valvocopula external poration” were treated as additive in the analysis. https://binary.pensoft.net/file/746751This dataset is made available under the Open Database License (http://opendatacommons.org/licenses/odbl/1.0/). The Open Database License (ODbL) is a license agreement intended to allow users to freely share, modify, and use this Dataset while maintaining this same freedom for others, provided that the original source and author(s) are credited.Christopher S. Lobban, Matt P. Ashworth

Supplementary material 3 Figure S1. Plastids in a living cell of *Ardissoneaformosa* showing movement from dispersed (peristrophy) to concentrated around the nucleus (karyostrophy) Data type Images (jpg. file) Explanation note **Figure S1.** Plastids in a living cell of *Ardissoneaformosa* showing movement from dispersed (peristrophy) to concentrated around the nucleus (karyostrophy). Process already underway in Fig. S1A. Girdle views in A–C, F; valve views in D, E. Fig. **A** Entire living cell (length 456 µm) with plastids still mostly spread out. **B** Detail near pole showing shape of plastids. **C, D** Detail of central nucleus with surrounding plastids in girdle view (C) and 2:21 minutes later in valve view (D). **E** Pole of cell in valve view for identification. E. Same living cell 9:00 minutes after images in A and B, showing almost all plastids around the nucleus. Scale bars: 50 µm (A, F); 10 µm (B–E). https://binary.pensoft.net/file/746752This dataset is made available under the Open Database License (http://opendatacommons.org/licenses/odbl/1.0/). The Open Database License (ODbL) is a license agreement intended to allow users to freely share, modify, and use this Dataset while maintaining this same freedom for others, provided that the original source and author(s) are credited.Christopher S. Lobban

Supplementary material 4 Figure S2. *Toxarium* sp. from Guam Data type SEM images (jpg. file) Explanation note **Figure S2.***Toxarium* sp. from Guam. **A–C** SEM showing rippled margin and single row of mantle pores. **A–C** GU44S / HK210 / ECT 3802. **D–F** Wild specimen from GU44BK-1 showing undulate outline but single row of areolae on mantle (F, arrow). Scale bars: 50 µm (**A, D**), 10 µm (**E, F**), 5 µm (**B, C**). https://binary.pensoft.net/file/746753This dataset is made available under the Open Database License (http://opendatacommons.org/licenses/odbl/1.0/). The Open Database License (ODbL) is a license agreement intended to allow users to freely share, modify, and use this Dataset while maintaining this same freedom for others, provided that the original source and author(s) are credited.Matt P. Ashworth, Christopher S. Lobban

Supplementary material 5 Figure S3. Molecular phylogenetic tree illustrating the results of the RAxML Maximum Likelihood analysis of the 3-gene dataset Data type Phylogenetic tree (pdf. file) Explanation note **Figure S3.** Molecular phylogenetic tree illustrating the results of the RAxML Maximum Likelihood analysis of the 3-gene dataset (nuclear-encoded small subunit rDNA, chloroplast-encoded *rbcL* and *psb*C markers). The taxon *Bolidomonaspacifica* was used as an outgroup and all taxa labels are followed by Extraction Voucher ID or Strain ID. Bootstrap support values are present at each corresponding node. This is the complete tree which corresponds to Fig. 33A. https://binary.pensoft.net/file/746754This dataset is made available under the Open Database License (http://opendatacommons.org/licenses/odbl/1.0/). The Open Database License (ODbL) is a license agreement intended to allow users to freely share, modify, and use this Dataset while maintaining this same freedom for others, provided that the original source and author(s) are credited.Matt P. Ashworth, Daryl W. Lam

Supplementary material 6 Figure S4. Molecular phylogenetic tree illustrating the results of the IQTREE Maximum Likelihood analysis of the 3-gene dataset Data type Phylogenetic tree (pdf. file) Explanation note **Figure S4.** Molecular phylogenetic tree illustrating the results of the IQTREE Maximum Likelihood analysis of the 3-gene dataset (nuclear-encoded small subunit rDNA, chloroplast-encoded *rbcL* and *psb*C markers). The taxon *Bolidomonaspacifica* was used as an outgroup and all taxa labels are followed by Extraction Voucher ID or Strain ID. Bootstrap support values are present at each corresponding node. This is the complete tree which corresponds to Fig. 33B. https://binary.pensoft.net/file/746755This dataset is made available under the Open Database License (http://opendatacommons.org/licenses/odbl/1.0/). The Open Database License (ODbL) is a license agreement intended to allow users to freely share, modify, and use this Dataset while maintaining this same freedom for others, provided that the original source and author(s) are credited.Matt P. Ashworth, Daryl W. Lam

Supplementary material 7 Figure S5. Molecular phylogenetic tree illustrating the results of the ExaBayes Bayesian Inference analysis of the 3-gene dataset Data type Phylogenetic tree (pdf. file) Explanation note **Figure S5.** Molecular phylogenetic tree illustrating the results of the ExaBayes Bayesian Inference analysis of the 3-gene dataset (nuclear-encoded small subunit rDNA, chloroplast-encoded *rbcL* and *psb*C markers). The taxon *Bolidomonaspacifica* was used as an outgroup and all taxa labels are followed by Extraction Voucher ID or Strain ID. Posterior probability values are present at each corresponding node. This is the complete tree which corresponds to Fig. 33C. https://binary.pensoft.net/file/746756This dataset is made available under the Open Database License (http://opendatacommons.org/licenses/odbl/1.0/). The Open Database License (ODbL) is a license agreement intended to allow users to freely share, modify, and use this Dataset while maintaining this same freedom for others, provided that the original source and author(s) are credited.Matt P. Ashworth, Daryl W. Lam
