# Supplementary material for: ﻿Revision of Ardissoneaceae (Bacillariophyta, Mediophyceae) from Micronesian populations, with descriptions of two new genera, Ardissoneopsis and Grunowago, and new species in Ardissonea, Synedrosphenia and Climacosphenia
Source: PhytoKeys. 2022 Sep 21;208:103–84. doi: 10.3897/phytokeys.208.89913 (PMC9848972; doi:10.3897/phytokeys.208.89913)
Supplement: Supplementary material 1 — Table S1. Metadata for strains used in the molecular phylogenetic analysis, including strain extraction voucher ID and GenBank accession numbers [file phytokeys-208-103_article-89913__-s001.doc]

Table S1. Taxa, strain voucher ID and GenBank accession numbers for strains used in the DNA sequence data phylogenetic analysis. Collection site for sample of original strain isolation or culture ID for the strain are also included (where known). Taxa are listed alphabetically. If species unknown, authority for genus is listed.

| Taxon | Extraction Voucher (HK###) | Strain or Collection ID  (Locality in parentheses) | GenBank Accession  (SSU, *rbc*L, *psb*C) |
| --- | --- | --- | --- |
| *Acanthoceras zachariasii* (Brun) Simonsen | HK250 | 6VII09-1A (Lake Okoboji, Iowa) | HQ912676, HQ912540, HQ912367 |
| *Achnanthes coarctata* (Brébisson ex W. Smith) Grunow in Cleve & Grunow | HK079 | FD185 (UTEX) | HQ912594, HQ912458, HQ912287 |
| *Achnanthes* sp Bory | HK303 | SanNicholas1 (San Nicholas, Canary Islands) | KC309473, KC309545, KC309617 |
| *Achnanthes* sp Bory | HK309 | ECT3883 (Rainbow Harbor, Long Beach, California) | KC309474, KC309546, KC309618 |
| *Achnanthes* sp Bory | HK310 | ECT3911 (Long Beach, California) | KC309475, KC309547, KC309619 |
| *Achnanthes* sp Bory | HK311 | ECT3684 (Achang Reef, Guam) | KC309476, KC309548, KC309620 |
| *Achnanthes* sp Bory | HK517 | Azo42 (Azores) | MH063437, MH064054, MH063967 |
| *Achnanthes* sp Bory | HK651 | P1 12-8-13 (Pensacola Bay, Florida) | MW327161, MW324579, MW324633 |
| *Achnanthes* sp Bory | HK652 | GU52AB (Outhouse Beach, Guam) | MW327162, MW324580, MW324634 |
| *Achnanthes* sp Bory | HK680 | 8IV18-1C (Tybee Island, Georgia) | OP304747, OP297418, OP297477 |
| *Achnanthes* sp Bory | UTKSA0263 | KSA2015-16 (Al-Nawras, Jeddah, Saudi Arabia) | MH063438, MH064055, N/A |
| *Achnanthidium minutissimum* (Kützing) Czarnecki |  |  | AM502032, AM710499, N/A |
| *Actinocyclus octonarius* Ehrenberg |  | AJA022-31 | OP304748, OP297419, OP297478 |
| *Actinocyclus octonarius* Ehrenberg | HK270 | ECT3845 (Corpus Christi Bay, Texas) | OP304749, OP297420, OP297479 |
| *Actinocyclus* sp Ehrenberg | HK262 | CVPan-4 (STRI Station, Panama) | KC309524, KC309597, KC309670 |
| *Actinocyclus* sp Ehrenberg | HK345 | ECT3910 (Key Largo, Florida) | KC309521, KC309594, KC309667 |
| *Actinocyclus* sp Ehrenberg | HK346 | ECT3899 (Pebble Beach, California) | KC309522, KC309595, KC309668 |
| *Actinocyclus* sp Ehrenberg | HK347 | GU52-O (Outhouse Beach, Guam) | KC309523, KC309596, KC309669 |
| *Actinocyclus* sp Ehrenberg | HK610 | 16VIII13-1 (Bar Harbor, Maine) | OP304750, OP297421, N/A |
| *Actinocyclus* sp Ehrenberg | HK611 | 21IV14-6A (Sprigger Bank, Florida) | N/A, OP297422, OP297480 |
| *Actinocyclus subtilis* (W. Gregory) Ralfs | HK168 | ECT3672 (Haputo Point, Guam) | HQ912640, HQ912504, HQ912333 |
| *Actinoptychus* sp Ehrenberg | HK050 | SA19Ai4 (South Africa) | HQ912574, HQ912438, HQ912267 |
| *Actinoptychus* sp Ehrenberg | HK382 | FijiBottle (New York) | KJ577841, KJ577876, KJ577912 |
| *Actinoptychus* cf *splendens* (Shadbolt) Ralfs | HK381 | ECT3920 (Ft. Stevens Park, Oregon) | KJ577840, KJ577875, N/A |
| *Actinoptychus* cf *splendens* (Shadbolt) Ralfs | HK612 | RO400m (Angola) | OP304751, OP297423, OP297481 |
| *Actinoptychus splendens* (Shadbolt) Ralfs | HK383 | 25VI12-1A SC (Hunting Island, South Carolina) | KJ577842, KJ577877, KJ577913 |
| *Actinoptychus undulatus* (Kützing) Ralfs | HK261 | 17iii09-2F CA (Stillwater Cove, California) | KC309525, KC309598, KC309671 |
| *Adlafia brockmannii* (Hustedt) Bruder & Hinz |  |  | AM502020, AM710487, N/A |
| *Ambo tenuissimus* (Hustedt) Witkowski, Lange-Bertalot & Ashworth | HK546 | DK004 (Senegal) | N/A, MN917216, MN917229 |
| *Amphipentas pentacrinus* Ehrenberg | HK289 | ECT3874 (Channel #5, Florida Bay, Florida) | KC309483, KC309555, KC309626 |
| *Amphipentas pentacrinus* Ehrenberg | HK384 | Coz-1 (Cozumel, Mexico) | KJ577843, KJ577878, KJ577914 |
| *Amphipentas pentacrinus* Ehrenberg | HK582 | PR2 (Condado Lagoon, Puerto Rico) | OP304752, OP297424, OP297482 |
| *Amphipentas pentacrinus* Ehrenberg | HK583 | Azo12 (Azores) | OP304753, OP297425, OP297483 |
| *Amphipleura pellucida* Kützing | HK287 | ECT3568 (Lake Travis, Texas) | KC309477, KC309549, KC309621 |
| *Amphitetras antediluviana* Ehrenberg | HK223 | ECT3627 (Montana de Oro State Park, California) | HQ912665, HQ912529, HQ912358 |
| *Amphitetras antediluviana* Ehrenberg | HK584 | Azo12 (Azores) | OP304754, OP297426, OP297484 |
| *Amphora aliformis* | AMPH177 |  | KP229525, KP229546, KP229548 |
| *Amphora caribaea* | AMPH086 |  | KJ463428, KJ463458, KJ463488 |
| *Amphora commutata* | AMPH126 |  | KP229526, KP229547, KP229549 |
| *Amphora helenensis* | SZCZCH704 |  | KT943649, KT943672, KT943709 |
| *Amphora hyalina* | AMPH136 |  | KJ463432, KJ463462, KJ463492 |
| *Amphora lineolata* | AMPH035 |  | KJ463435, KJ463465, KJ463495 |
| *Amphora obtusa* Gregory | UTKSA0275 | KSA2015-37 (Rabigh, Saudi Arabia) | MH063440, MH064057, N/A |
| *Amphora obtusa* v *crassa* | AMPH070 |  | KJ463436, KJ463466, KJ463496 |
| *Amphora pediculus* (Kützing) Grunow |  | L1030 (UTEX) | HQ912417, HQ912403, HQ912389 |
| *Amphora securicula* | AMPH046 |  | KJ463440, KJ463470, KJ463500 |
| *Amphora* sp Ehrenberg ex Kützing | HK502 | PackaryChannelSediment (Mustang Island, Texas) | MH017634, MH064058, MH063969 |
| *Amphora* sp Ehrenberg ex Kützing | UTKSA0087 | SA12 (Markaz Al Shoaibah, Saudi Arabia) | MH063441, MH064059, MH063970 |
| *Amphora* sp Ehrenberg ex Kützing | UTKSA0115 | KSA2015-27 (Markaz Al Shoaibah, Saudi Arabia) | MH063442, MH064060, N/A |
| *Amphora* sp Ehrenberg ex Kützing | UTKSA0137 | KSA2015-11 (Bhadur resort, Saudi Arabia) | OP304755, N/A, N/A |
| *Amphora* sp Ehrenberg ex Kützing | UTKSA0138 | KSA2015-11 (Bhadur resort, Saudi Arabia) | MW327163, MW324581, MW324635 |
| *Amphora* sp Ehrenberg ex Kützing | UTKSA0153 | KSA2015-37 (Rabigh, Saudi Arabia) | MH063443, MH064061, MH063971 |
| *Amphora* cf *immarginata* Nagumo | UTKSA0172 | KSA2015-37 (Rabigh, Saudi Arabia) | MH063439, MH064056, MH063968 |
| *Amphora* sp Ehrenberg ex Kützing | UTKSA0177 | KSA2015-41 (Rabigh, Saudi Arabia) | MH063444, MH064062, MH063972 |
| *Amphora sublaevis* | AMPH135 |  | KJ463444, KJ463474, KJ463504 |
| *Amphora subtropica* | AMPH051 |  | KJ463445, KJ463475, KJ463505 |
| *Amphora sulcata* | AMPH083 |  | KJ463446, KJ463476, KJ463506 |
| *Amphora vixvisibilis* Li & Witkowski | SZCZCH967 |  | KT943648, KT943670, KT943706 |
| *Amphora waldeniana* | AMPH011 |  | KJ463447, KJ463477, KJ463507 |
| *Anaulus* cf *australis* Drebes & Schulz |  | ANAAUS1 | MN917238, MN917213, MN917226 |
| *Anaulus* cf *australis* Drebes & Schulz |  | ANAAUS2 | MN917239, MN917214, MN917227 |
| *Anomoeoneis fogedii* Reimer |  | FD399 (UTEX) | KJ011610, KJ011793, N/A |
| *Anomoeoneis sphaerophora* Pfitzer |  | FD160 (UTEX) | KJ011612, KJ011795, N/A |
| *Arcocellulus mammifer* Hasle, von Stosch & Syvertsen | HK044 | CCMP132 | HQ912569, HQ912433, HQ912263 |
| *Arcocellulus* sp Hasle, von Stosch & Syvertsen |  | AJA020-4 | OP304756, OP297427, OP297485 |
| *Ardissonea baculus* |  | WK76 | AM746973, AB430664, N/A |
| *Ardissonea formosa* (Hantzsch in Rabenhorst) Grunow in Cleve & Grunow | HK209 | ECT3655 (Gab Gab Beach, Guam) | HQ912653, HQ912517, HQ912346 |
| *Ardissonea* sp De Notaris | UTKSA0041 | SA29 (Jeddah, Saudi Arabia) | OP304757, OP297428, OP297486 |
| *Ardissoneopsis appressata* Lobban & Ashworth | HK167 | ECT3655 (Gab Gab Beach, Guam) | HQ912639, HQ912503, HQ912332 |
| *Ardissoneopsis fulgicans* (Greville) Lobban & Ashworth | HK305 | 26VI10-2A (Bald Head Island, North Carolina) | KC309484, KC309556, KC309627 |
| *Astartiella almalikii* Sabir, Ashworth & Górecka | UTKSA0146 | KSA2015-11 (Bhadur Resort, Saudi Arabia) | MH063445, MH064063, MH063973 |
| *Astartiella* sp A.Witkowski, Lange-Bertalot & Metzeltin | HK719 | Tombwa Bay (Angola) | OP304758, OP297429, OP297487 |
| *Asterionella formosa* Hassall | HK144 | UTCC605 | HQ912633, HQ912497, HQ912326 |
| *Asterionellopsis glacialis* (Castracane) Round | HK107 | CCMP134 | HQ912613, HQ912477, HQ912306 |
| *Asterionellopsis socialis* (Lewin & Norris) Crawford & Gardner | HK181 | CCMP1717 | HQ912646, HQ912510, HQ912339 |
| *Asterionellopsis socialis* (Lewin & Norris) Crawford & Gardner | HK319 | ECT3920 (Ft. Stevens State Park, Oregon) | JX413545, JX413562, JX413579 |
| *Asteromphalus sp.* Ehrenberg | HK386 | ECT3832 (Laguna Beach, California) | KJ577845, KJ577880, N/A |
| *Astrosyne radiata* Ashworth et Lobban | HK169 | ECT3697 (Gab Gab Beach, Guam) | JN975238, JN975252, JN975267 |
| *Attheya armata* (West) Crawford | HK670 |  | MZ546200, MZ542379, MZ542398 |
| *Attheya longicornis* Crawford & Gardner in Crawford, Gardner & Medlin | HK317 | CCMP214 | JX401230, JX401247, JX401265 |
| *Attheya septentrionalis* (Østrup) Crawford in Crawford, Gardner & Medlin | HK112 | CCMP2084 | HQ912618, HQ912482, HQ912311 |
| *Aulacodiscus orientalis* Greville | HK208 | ECT3746 (Talofofo Bay, Guam) | HQ912652, HQ912516, HQ912345 |
| *Aualcodiscus oreganus* Harvey & Bailey | HK348 | ECT3897 (Stillwater Cove, California) | JX413556, JX413573, JX413590 |
| *Aulacoseira granulata* (Ehrenberg) Simonsen | HK094 | FD301 (UTEX) | HQ912606, HQ912470, HQ912299 |
| *Auricula sp.* Castracane | HK434 | 21IV14-4D (Rabbit Key Basin, Florida) | KX981842, KX981810, KX981789 |
| *Auricula cf complexa* (Gregory) Cleve | UTKSA0038 | SA12 (Markaz Al Shoaibah, Saudi Arabia) | MH063446, MH064064, MH063974 |
| *Auricula cf flabelliformis* M. Voigt | UTKSA0071 | SA12 (Markaz Al Shoaibah, Saudi Arabia) | MH063447, MH064065, MH063975 |
| *Bacillaria paxillifer* (O. F. Müller) T. Marsson | HK130 | FD468 (UTEX) | HQ912627, HQ912491, HQ912320 |
| *Bacillaria* sp Gmelin | HK475 | GU44BK-1 (Gab Gab Beach, Guam) | MH063448, MH064066, MH063976 |
| *Bacillaria* sp Gmelin | HK663 | GU44BR-5 (Gab Gab Beach, Guam) | MW327167, MW324587, MW324640 |
| *Bacillaria* sp Gmelin | UTKSA0009 | SA27 (Jeddah, Saudi Arabia) | MH063449, MH064067, MH063977 |
| *Bacillaria* sp Gmelin | UTKSA0129 | KSA2015-9 (Bhadur resort, Saudi Arabia) | MH063450, MH064068, MH063978 |
| *Bacillaria* sp Gmelin | UTKSA0130 | KSA2015-9 (Bhadur resort, Saudi Arabia) | MH063451, MH064069, MH063979 |
| *Bellerochea horologicalis* von Stosch | HK235 | ECT3829 (Redfish Bay, Texas) | HQ912672, HQ912536, HQ912364 |
| *Bellerochea malleus* (Brightwell) Van Heurck | HK265 | Har-1 (HBOI Boat Dock, Florida) | KC309485, KC309557, KC309628 |
| *Bellerochea yucatanensis* Stosch | HK387 | WhiskeyCreek4 (Whiskey Creek, Florida) | KJ577846, KJ577881, KJ577916 |
| *Berkeleya hyalina* (F.E.Round & M.E.Brooks) E.J.Cox | HK388 | ECT3614 (La Jolla, California) | KJ577847, KJ577882, KJ577917 |
| *Berkeleya rutilans* (Trentepohl ex Roth) Grunow | HK154 | ECT3616 (Laguna Beach, California) | HQ912637, HQ912501, HQ912330 |
| *Berkeleya rutilans* (Trentepohl ex Roth) Grunow | HK389 | ECT3602 (Bolinas, California) | KJ577848, KJ577883, KJ577918 |
| *Berkeleya rutilans* (Trentepohl ex Roth) Grunow | HK498 | CGA1605-D (manatee, Georgia) | MH017635, MW324588, N/A |
| *Biddulphia alternans* (J.W. Bailey) Van Heurck | HK292 | ECT3886 (Bald Head Island, North Carolina) | JX401229, JX401246, JX401264 |
| *Biddulphia alternans* (J.W. Bailey) Van Heurck | HK541 | Azo12 (Azores) | MN917243, MN917220, MN917233 |
| *Biddulphia alternans* (J.W. Bailey) Van Heurck | HK542 | 25VI12-1A (Hunting Island, South Carolina) | MN917244, MN917221, MN917232 |
| *Biddulphia alternans* (J.W. Bailey) Van Heurck | HK591 | Atl.Plank#8 (Atlantic Coast, Florida) | OP304759, OP297430, OP297488 |
| *Biddulphia alternans* (J.W. Bailey) Van Heurck | HK592 | AJA028-28 (Georgia) | MZ546201, MZ542380, MZ542399 |
| *Biddulphia biddulphiana* (J.E. Smith) Boyer | HK271 | ClayHI (Kona, Hawaii) | JN975239, JN975253, JN975268 |
| *Biddulphia biddulphiana* (J.E. Smith) Boyer | HK328 | ECT3902 (Channel #5, US-1, Florida) | JX401227, JX401244, JX401262 |
| *Biddulphia biddulphiana* (J.E. Smith) Boyer | HK452 | GU44AK-4 (Gab Gab Beach, Guam) | MN917242, MN917219, MN917232 |
| *Biddulphia biddulphiana* (J.E. Smith) Boyer | HK543 | Tong Yeong Station (South Korea) | MN917241, MN917218, MN917231 |
| *Biddulphia biddulphiana* (J.E. Smith) Boyer | HK547 | 19X15-1B (Channel #5, US-1, Florida) | OP304760, OP297431, OP297489 |
| *Biddulphia sculpta* (Shadbolt) Van Heurck | HK252 | ECT3856 (Kahana Bay, Oahu, Hawaii) | HQ912677, HQ912541, KC309629 |
| *Biddulphia* cf *sculpta* (Shadbolt) Van Heurck | HK329 | ECT3891 (St. George Island, Florida) | KC309486, KC309558, KC309630 |
| *Biddulphia tridens* (Ehrenberg) Ehrenberg | HK239 | CCMP3358 | HQ912674, HQ912538, HQ912365 |
| *Biddulphia tridens* (Ehrenberg) Ehrenberg | HK327 | ECT3902 (Channel #5, US-1, Florida) | JX401228, JX401245, JX401263 |
| *Biddulphia tridens* (Ehrenberg) Ehrenberg | HK711 | PG#12-1 (Iran) | OP304761, OP297432, OP297490 |
| *Biddulphiopsis membranacea* (Cleve) von Stosch & Simonsen | HK166 | ECT3655 (Gab Gab Beach, Guam) | HQ912638, HQ912502, HQ912331 |
| *Biddulphiopsis titiana* (Grunow) von Stosch & Simonsen | HK170 | ECT3697 (Gab Gab Beach, Guam) | HQ912641, HQ912505, HQ912334 |
| *Biremis* sp D.G. Mann & E.J. Cox | HK438 | 21IV14-2A (Duck Key, Florida) | KX981835, KX981811, N/A |
| *Bleakeleya notata* (Grunow in Van Heurck) F.E. Round | HK247 | ECT3733 (Pago Bay, Guam) | HM627330, HM627327, HM627324 |
| *Bolidomonas pacifica* L.Guillou & M.-J.Chrétiennot-Dinet | HK015 | CCMP1866 | HQ912557, HQ912421, HQ912251 |
| *Brachysira* sp Kützing | HK692 | PackChannPlank (Packary Channel, Mustang Island, Texas) | OP304762, OP297433, OP297491 |
| *Brockmanniella brockmannii* (Hustedt) Hasle, von Stosch & Syvertsen | HK040 | CCMP151 | HQ912565, HQ912429, HQ912259 |
| *Caloneis* cf *excentrica* (Grunow) Boyer | HK431 | 21IV14-2A (Duck Key, Florida) | KU179130, KU179117, KU179144 |
| *Caloneis lewisii* Patrick | HK060 | FD54 (UTEX) | HQ912580, HQ912444, HQ912273 |
| *Caloneis* cf *linearis* (Cleve) Boyer | HK430 | 21IV14-3A (Captain’s Key, Florida) | KU179132, KU179119, KU179146 |
| *Caloneis* sp P.T. Cleve | HK429 | SantaRosa cor.green (Costa Rica) | KU179134, KU179123, N/A |
| *Caloneis* sp P.T. Cleve | HK432 | 21IV14-6A FL (Sprigger Bank, Florida) | KU179131, KU179118, KU179145 |
| *Caloneis* sp P.T. Cleve | HK477 | GU7Y-4 (University of Guam Marine Laboratories, Guam) | MH063453, MH064071, MH063981 |
| *Caloneis* sp P.T. Cleve | HK479 | GU52V-2 (Outhouse Beach, Guam) | N/A, MH064072, MH063982 |
| *Caloneis* sp P.T. Cleve | HK480 | 21IV14-2A FL (Duck Key, Florida) | MW327168, MW324589, MW324641 |
| *Caloneis* sp P.T. Cleve | HK481 | 21IV14-3A (Captain’s Key, Florida) | N/A, MW324590, MW324642 |
| *Caloneis* sp P.T. Cleve | HK529 | Tong Yeong Station (South Korea) | MW327171, MW324595, MW324646 |
| *Caloneis* sp P.T. Cleve | UTKSA0235 | KSA2015-37 (Rabigh, Saudi Arabia) | MH063454, MH064073, MH063983 |
| *Caloneis* sp P.T. Cleve | UTKSA0252 | KSA2015-42 (Rabigh, Saudi Arabia) | MH063455, MH064074, MH063984 |
| *Caloneis* cf *westii* |  | SZCZCH1002 | KT943628, KT943654, KT943687 |
| *Campylodiscus clypeus* (Ehrenberg) Kützing |  | L951 (UTEX) | HQ912412, HQ912398, HQ912384 |
| *Campylodiscus* sp Ehrenberg ex Kützing |  | ECT3613 (Tomales Bay, California) | HQ912413, HQ912399, HQ912385 |
| *Campylodiscus* sp Ehrenberg ex Kützing | HK653 | GU44BR-3 (Gab Gab Beach, Guam) | MW327169, MW324591, MW324643 |
| *Campylodiscus* sp Ehrenberg ex Kützing | UTKSA0284 | KSA2015-29 (Markaz Al Shoaibah, Saudi Arabia) | MH063456, MH064075, N/A |
| *Campylosira africana* Giffen |  | SZCZP1004 | MF001980, MF001950, MF001922 |
| *Campylosira africana* Giffen |  | SZCZP860 | MF001979, MF001949, MF001921 |
| *Campylosira cymbelliformis* (Schmidt) Grunow in Van Heurck | HK122 | CCC-1 (Corpus Christi Bay, Texas) | HQ912623, HQ912487, HQ912316 |
| *Campylosira cymbelliformis* (Schmidt) Grunow in Van Heurck | HK518 | 24VI12-2A (Savannah, Georgia) | OP304763, OP297434, OP297492 |
| *Campylosira cymbelliformis* (Schmidt) Grunow in Van Heurck |  | SZCZM1633 | N/A, MF001951, MF001923 |
| *Carinasigma minuta* (Donkin) G. Reid | HK418 | GU7X-6 (University of Guam Marine Lab, Guam) | KX981841, KX981812, KX981790 |
| *Carinasigma* sp G. Reid | UTKSA0269 | KSA2015-37 (Rabigh, Saudi Arabia) | MH063463, MH064087, MH063995 |
| *Carinasigma* sp G. Reid | UTKSA0287 | KSA2015-54 (Duba, Saudi Arabia) | MW327173, N/A, MW324649 |
| *Castoridens hyalina* Ashworth, Witkowski & Li | HK444 | C1 12-7-13 (Destin-Choctawhatchee Bay, Florida) | N/A, KU851892, KU851907 |
| *Castoridens striata* Ashworth, Ch. Li & Witkowski | HK385 | ECT3916 (Baffin Bay, Texas) | KJ577844, KJ577879, KJ577915 |
| *Catacombas* sp*.* D.M. Williams & Round |  | s0045 | KR048195, KR048217, KR048229 |
| *Catacombas gaillonii* (Bory) Williams & Round |  | WK37 | EF423402, MG684346, MG684315 |
| *Centronella reicheltii* Voigt | HK150 | CCAP1011/1 | HQ912635, HQ912499, HQ912328 |
| *Ceratanaulus creticus* (Drebes & Schulz) Górecka, Witkowski, Dąbek & Ashworth |  | SZCZE681 | MN917240, MN917217, MN917230 |
| *Cerataulina bicornis* (Ehrenberg) Hasle | HK390 | AtlanticPlankton#8 (Atlantic Coast, Florida) | KJ577849, KJ577884, KJ577919 |
| *Cerataulina pelagica* (Cleve) Hendey | HK230 | ECT3845 (Ward Island, Texas) | HQ912669, HQ912533, HQ912361 |
| *Cerataulina pelagica* (Cleve) Hendey | HK232 | ECT3836 (Rainbow Harbor, Long Beach, California) | KC309487, KC309559, KC309631 |
| *Cerataulina pelagica* (Cleve) Hendey | HK596 | ECT3884 (Pacific Grove, California) | OP304764, N/A, N/A |
| *Cerataulus turgidus* (Ehrenberg) Ehrenberg | HK699 | Azo12 (Azores) | OP304765, OP297435, OP297493 |
| *Chaetoceros* cf *lorenzianus* Grunow | UTKSA0020 | SA18 (Duba, Saudi Arabia) | OP304766, OP297436, OP297494 |
| *Chaetoceros muelleri* Lemmermann | HK028 | CCMP1316 | HQ912558, HQ912422, HQ912252 |
| *Chaetoceros peruvianus* Brightwell | HK202 | ECT3821 (Ward Island, Texas) | HQ912650, HQ912514, HQ912343 |
| *Chaetoceros simplex* Ostenfeld |  | CCMP200 | N/A, KJ958479 |
| *Chaetoceros sp.* Ehrenberg | HK587 | 17III13-4 (Wiscasset, Maine) | OP304767, N/A, N/A |
| *Chaetoceros* sp Ehrenberg | UTKSA0016 | SA18 (Duba, Saudi Arabia) | OP304768, OP297437, OP297495 |
| *Chaetoceros* sp Ehrenberg | UTKSA0045 | SA18 (Duba, Saudi Arabia) | OP304769, OP297438, OP297496 |
| *Chrysanthomodiscus floriatus* Mann | HK171 | ECT3710 (Haputo Point, Guam) | HQ912642, HQ912506, HQ912335 |
| *Climacodium* *frauenfeldianum* Grunow |  | Fernando22 | MK027054, MK108030, N/A |
| *Climaconeis riddleae* Prasad | HK178 | ECT3724 (Umatac Bay, Guam) | HQ912644, HQ912508, HQ912337 |
| *Climaconeis undulata* (Meister) Lobban et al | HK218 | ECT3743 (Talofofo Bay, Guam) | KC309478, KC309550, N/A |
| *Climaconeis* sp Grunow | UTKSA0040 | SA26 (Jeddah, Saudi Arabia) | KX981836, KX981813, N/A |
| *Climacosphenia* sp Ehrenberg | HK276 | ECT3758 (Scuba Beach, Guam) | HQ912685, HQ912549, HQ912371 |
| *Climacosphenia* sp Ehrenberg | HK590 | GU44AI (Gab Gab Beach, Guam) | OP304770, OP297439, N/A |
| *Climacosphenia moniligera* Ehrenberg |  | s0253 | AM746974, N/A, N/A |
| *Cocconeis convexa* Giffen | HK312 | ECT3901 (Channel #5, US-1, Florida) | KC309479, KC309551, KC309622 |
| *Cocconeis placentula* Ehrenberg | HK077 | FD23 (UTEX) | HQ912592, HQ912456, HQ912285 |
| *Cocconeis stauroneiformis* (W. Smith) H. Okuna | s0230 |  | AB430614, AB430694, N/A |
| *Cocconeis* sp Ehrenberg |  | SZCZP67 | KT943600, KT943614, KT943625 |
| *Cocconeis* sp Ehrenberg | UTKSA0056 |  | KU179133, KU179120, KU179147 |
| *Corethron hystrix* Hensen | HK004 | CCMP307 | HQ912554, HQ912418, HQ912248 |
| *Corethron hystrix* Hensen | HK241 | Har-1 (HBOI Boat Dock, Florida) | KC309526, KC309599, KC309672 |
| *Corethron* sp Castracane | HK356 | ECT3920 (Ft. Stevens State Park, Oregon) | KC309527, KC309600, KC309673 |
| *Corethron* sp Castracane | HK671 | CGA1607-T (manatee, Georgia) | MZ546202, MZ542382, MZ542402 |
| *Coscinodiscus concinnus* W. Smith | HK267 | ECT3839 (Port O’Connor, Texas) | HQ912681, HQ912545, HQ912369 |
| *Coscinodiscus granii* Gough | HK228 | ECT3836 (Rainbow Harbor, Long Beach, California) | HQ912667, HQ912531, HQ912359 |
| *Coscinodiscus jonesianus* (Greville) Ostenfeld | HK393 | 24VI12-1A (Savannah, Georgia) | KJ577852, KJ577887, KJ577922 |
| *Coscinodiscus radiatus* Ehrenberg | HK031 | CCMP310 | HQ912560, HQ912424, HQ912254 |
| *Coscinodiscus radiatus* Ehrenberg | HK352 | ECT3923 (The Evergreen State University, Washington) | KC309529, KC309602, KC309674 |
| *Coscinodiscus* sp Ehrenberg | HK263 | CVPan-4 (Panama) | KC309531, KC309604, KC309676 |
| *Coscinodiscus* sp Ehrenberg | HK298 | ECT3874 (Channel #5, Florida Bay, Florida) | KC309530, KC309603, KC309675 |
| *Coscinodiscus* sp Ehrenberg | HK392 | ECT3850 (Hawaii) | KJ577851, KJ577886, KJ577921 |
| *Coscinodiscus* sp Ehrenberg | HK394 | 14VI10-2A (Long Beach, California) | KJ577853, KJ577888, KJ577923 |
| *Coscinodiscus* sp Ehrenberg | HK350 | ECT3891 (St. George Island, Florida) | KC309532, KC309605, KC309677 |
| *Coscinodiscus* sp Ehrenberg | HK351 | ECT3900 (Moss Landing, California) | KC309533, KC309606, N/A |
| *Coscinodiscus wailesii* Gran & Angst | HK229 | ECT3847 (Port Aransas ferry crossing, Texas) | HQ912668, HQ912532, HQ912360 |
| *Craspedostauros* *alatus* Majewska & Ashworth | HK448 | CCMP1120 (NCMA) | KX981860, KX981817, KX981793 |
| *Craspedostauros* *alyoubii* J. Sabir & Ashworth | UTKSA0083 | SA18 (Duba, Saudi Arabia) | KX981857, KX981814, KX981791 |
| *Craspedostauros amphoroides* (Grunow) Cox | HK447 | CCMP797 (NCMA) | KX981859, KX981815, N/A |
| *Craspedostauros paradoxus* Ashworth & Lobban | HK441 | GU44BK-1 (Gab Gab Beach, Guam, USA) | KX981858, KX981816, KX981792 |
| *Cratericulifera crinigera* (Takano) Ashworth, Li & Witkowski | HK506 |  | N/A, MF092971, MF093054 |
| *Cratericulifera* *shandongensis* Li, Witkowski & Ashworth |  | SZCZCH1247 | N/A, KU851893, KU851908 |
| *Cratericulifera* sp Ch. Li, Witkowski & Ashworth | HK662 | CGA1605T (manatee, Georgia) | N/A, MW324592, N/A |
| *Craticula cuspidata* (Kützing) Mann | HK061 | FD35 (UTEX) | HQ912581, HQ912445, HQ912274 |
| *Craticula cuspidata* (Kützing) Mann | HK499 | FLMan10 (manatee, Crystal River, Florida) | MH017636, MW324593, MW324644 |
| *Ctenophora pulchella* (Ralfs ex Kuetzing) Williams & Round | HK105 | FD150 (UTEX) | HQ912611, HQ912475, HQ912304 |
| *Cyclophora castracanei* Ashworth & Lobban | HK243 | GU44AB-6 (Gab Gab Beach, Guam) | JN975242, JN975256, JN975271 |
| *Cyclophora castracanei* Ashworth & Lobban | HK395 | GU44AN-7 (Gab Gab Beach, Guam) | KJ577854, KJ577889, N/A |
| *Cyclophora* cf *minor* Ashworth & Lobban | HK461 | 24IV14-3A (Pickles Reef, Florida) | MH040308, MH040254, MH040230 |
| *Cyclophora* sp Castracane | HK396 | Coz3 (Cozumel, Mexico) | N/A, KJ577890, KJ577924 |
| *Cyclophora tabellariformis* Ashworth et Lobban | HK306 | ECT3892 (Carrabelle, Florida) | JN975243, JN975257, JN975272 |
| *Cyclophora tabellariformis* Ashworth et Lobban | HK460 | GU44AY-6 (Gab Gab Beach, Guam) | MH040309, MH040255, N/A |
| *Cyclophora tenuis* Castracane | HK216 | ECT3723 (Umatac Bay, Guam) | HQ912660, HQ912524, HQ912353 |
| *Cyclophora tenuis* Castracane | HK307 | ECT3854 (Kahana Beach Park, Oahu, Hawaii) | JN975240, JN975254, JN975269 |
| *Cyclophora tenuis* Castracane | HK308 | ECT3838 (Long Beach, California) | JN975241, JN975255, JN975270 |
| *Cyclostephanos dubius* (Fricke) Round | HK051 | Waco5 (Lake Waco, Texas) | HQ912575, HQ912439, HQ912268 |
| *Cyclotella meneghiniana* Kützing | HK052 | Waco1 (Lake Waco, Texas) | HQ912576, HQ912440, HQ912269 |
| *Cyclotella sp* (F.T. Kützing) A. de Brébisson | HK126 | LO-4-2 (Lake Ohrid, Macedonia) | HQ912625, HQ912489, HQ912318 |
| *Cylindrotheca closterium* (Ehrenberg) Reimann & Lewin | HK180 | CCMP1855 (NCMA) | HQ912645, HQ912509, HQ912338 |
| *Cylindrotheca* sp Rabenhorst | UTKSA0079 | SA12 (Markaz Al Shoaibah, Saudi Arabia) | KX981848, KX981826, KX981801 |
| *Cylindrotheca* sp Rabenhorst | UTKSA0082 | SA18 (Duba, Saudi Arabia) | KX981847, KX981827, KX981802 |
| *Cymatoneis* sp Cleve | UTKSA0378 | KSA2016-3 (Bhadur Resort, Saudi Arabia) | MH063457, MH064076, MH063985 |
| *Cymatopleura elliptica* (Brebisson ex Kutzing) W. Smith | HK215 | L1333 (UTEX) | HQ912659, HQ912523, HQ912352 |
| *Cymatosira belgica* Grunow in Van Heurck | HK343 | ECT3892 (Carrabelle, Florida) | N/A, KC309563, KC309635 |
| *Cymatosira belgica* Grunow in Van Heurck | HK397 | 25VI12-1A (Hunting Island, South Carolina) | N/A, KJ577891, KJ577925 |
| *Cymatosira belgica* Grunow in Van Heurck |  | s0289 | N/A, MF001952, MF001924 |
| *Cymatosira belgica* Grunow in Van Heurck |  | SZCZCH80 | N/A, MF001953, MF001925 |
| *Cymatosira belgica* Grunow in Van Heurck |  | SZCZP1453 | N/A, MF001954, MF001926 |
| *Cymatosira lorenziana* Grunow | HK283 | ECT3874 (Channel #5, US-1, Florida) | KC309490, KC309562, KC309634 |
| *Cymatosira lorenziana* Grunow | HK588 | Coz3 (Cozumel) | OP304771, OP297440, OP297497 |
| *Cymatosira lorenziana* Grunow |  | SZCZCH114 | KU556408, KU556401, KU556394 |
| *Cymatosira lorenziana* Grunow |  | SZCZP4 | KU556407, KU556400, KU556393 |
| *Cymbella aspera* (Ehrenberg) Cleve |  | FD272 (UTEX) | KJ011615, KJ011797, N/A |
| *Cymbella proxima* Reimer |  |  | AM502017, AM710484, N/A |
| *Cymbopleura naviculiformis* (Auerswald ex Heiberg) Krammer |  |  | AM502004, AM710471, N/A |
| *Dactyliosolen blavyanus* (H. Peragallo) Hasle | HK301 | ECT3891 (St. George Island, Florida) | KC309491, KC309564, KC309636 |
| *Dactyliosolen fragilissimus* (Bergon) Hasle | HK426 | P1 12-8-13 (Pensacola Bay, Florida) | MZ546203, MZ542383, MZ542403 |
| *Delphineis sp* G.W. Andrews | HK133 | CCMP1095 | HQ912629, HQ912493, HQ912322 |
| *Delphineis surirella* (Ehrenberg) Andrews | HK295 | ECT3886 (Bald Head Island, North Carolina) | JX413544, JX413561, JX413578 |
| *Denticula kuetzingii* Grunow | HK104 | FD135 (UTEX) | HQ912610, HQ912474, HQ912303 |
| *Detonula confervacea* (Cleve) Gran | HK111 | CCMP353 | HQ912617, HQ912481, HQ912310 |
| *Detonula* sp Schütt ex De Toni | UTKSA0008 | SA29 (Jeddah, Saudi Arabia) | OP304772, OP297441, OP297498 |
| *Diatoma elongatum* (Lyngbye) Agardh | HK119 | UTCC62 | HQ912622, HQ912486, HQ912315 |
| *Diatoma tenuis* Agardh | HK078 | FD106 (UTEX) | HQ912593, HQ912457, HQ912286 |
| *Didymosphenia geminata* (Lyngbye) M. Schmidt | CH058 |  | KJ011636, KJ011819, N/A |
| *Dimeregramma* sp Ralfs in Pritchard | HK288 | ECT3864 (MSI, Port Aransas, Texas) | JN975244, JN975258, JN975273 |
| *Dimeregramma* sp Ralfs in Pritchard | HK358 | ECT3916 (Baffin Bay, Texas) | JX401231, JX401249, JX401267 |
| *Dimeregramma* sp Ralfs in Pritchard | HK359 | ECT3891 (St. George Island, Florida) | JX401232, JX401250, JX401268 |
| *Dimeregramma* sp Ralfs in Pritchard | HK376 | 25VI12-1C SC (Hunting Island, South Carolina) | KF701596, KF701605, KF701614 |
| *Dimeregramma* sp Ralfs in Pritchard | HK377 | AtlanticPlankton#8 (Atlantic coast, Florida) | KF701597, KF701606, KF701615 |
| *Diploneis* cf *cheronensis* (Grunow) Cleve | HK417 | GU44AY-6 (Gab Gab Beach, Guam) | MH017637, MH064077, MH063986 |
| *Diploneis* *parca* (Schmidt in Schmidt et al.) Boyer | UTKSA0267 | KSA2015-49 (Duba, Saudi Arabia) | MH063458, MH064078, MH063987 |
| *Diploneis* cf *smithii* (Brébisson in W. Smith) P.T. Cleve | HK437 | GU44AY-6 (Gab Gab Beach, Guam) | KX981837, KX981818, KX981794 |
| *Diploneis* cf *smithii* (Brébisson in W. Smith) P.T. Cleve | UTKSA0232 | KSA0215-30 (Markaz Al Shoaibah, Saudi Arabia) | MH063459, MH064079, N/A |
| *Diploneis* cf *smithii* (Brébisson in W. Smith) P.T. Cleve | UTKSA0238 | KSA2015-37 (Rabigh, Saudi Arabia) | MH063460, MH064080, MH063988 |
| *Diploneis* sp (Ehrenberg) P.T. Cleve | HK435 | Coz-4 (Cozumel, Mexico) | KX981839, KX981819, KX981795 |
| *Diploneis* sp (Ehrenberg) P.T. Cleve | HK436 | Coz-4 (Cozumel, Mexico) | KX981838, KX981820, KX981796 |
| *Diploneis* sp (Ehrenberg) P.T. Cleve | HK483 | 21IV14-2A (Duck Key, Florida) | MH017638, MH064081, MH063989 |
| *Diploneis* sp (Ehrenberg) P.T. Cleve | HK484 | PackaryChannelPlank (Mustang Island, Texas) | MH017639, MH064082, MH063990 |
| *Diploneis* sp (Ehrenberg) P.T. Cleve | UTKSA0181 | KSA2015-38 (Rabigh, Saudi Arabia) | OP304773, N/A, N/A |
| *Diploneis* sp (Ehrenberg) P.T. Cleve | UTKSA0190 | KSA2015-14 (Bhadur Resort, Saudi Arabia) | MH063461, MH064083, MH063991 |
| *Diploneis subovalis* Cleve | HK084 | FD282 (UTEX) | HQ912597, HQ912461, HQ912290 |
| *Ditylum brightwelli* Peragallo & Peragallo | HK285 | ECT3884 (Pacific Grove, California) | KC309493, KC309566, KC309638 |
| *Ditylum sol* (A. Schmidt) Cleve | HK240 | Har-1 (HBOI Boat Dock, Florida) | KC309492, KC309565, KC309637 |
| *Divergita al-ghamdii* Sabir & Theriot | UTKSA0237 | KSA2015-37 (Rabigh, Saudi Arabia) | MG684351, MG684321, MG684293 |
| *Divergita toxoneides* (Castracane) Theriot |  | WK57 | EF423421, MG684322, MG684294 |
| *Ellerbeckia* sp Crawford | HK398 | B16 (Lake Baikal, Russia) | KJ577855, KJ577892, KJ577926 |
| *Ellerbeckia* sp Crawford | HK609 | Lake Ohrid (Macedonia) | MZ546204, MZ542384, MZ542404 |
| *Encyonema norvegica* (Grunow) Mayer |  | FD342 (UTEX) | KJ011643, KJ011826, N/A |
| *Endictya oceanica* Ehrenberg | HK259 | Pan-1 (STRI Station, Panama) | KC309534, KC309607, KC309678 |
| *Entomoneis* sp Ehrenberg | HK 135 | CS782 (CSIRO) | HQ912631, HQ912495, HQ912324 |
| *Entomoneis* sp Ehrenberg | UTKSA0013 | SA12 (Markaz Al Shoaibah, Saudi Arabia) | N/A, MH064088, MH063996 |
| *Entomoneis* sp Ehrenberg | UTKSA0061 | SA18 (Duba, Saudi Arabia) | MH063464, MH064089, MH063997 |
| *Entomoneis* sp Ehrenberg | UTKSA0080 | SA12 (Markaz Al Shoaibah, Saudi Arabia) | MH063465, MH064090, MH063998 |
| *Entomoneis* sp Ehrenberg | UTKSA0092 | SA18 (Duba, Saudi Arabia) | MH063466, MH064091, MH063999 |
| *Epithemia argus* (Ehrenberg) Kützing | CH211 |  | HQ912408, HQ912394, HQ912380 |
| *Epithemia sorex* Kützing | CH148 |  | HQ912409, HQ912395, HQ912381 |
| *Eucampia cornuta* (Cleve) Grunow | HK399 | 24IV12-2 TX (Port O’Connor, Texas) | KJ577856, KJ577893, KJ577927 |
| *Eucampia sp* C.G. Ehrenberg | HK260 | ECT3836 (Rainbow Harbor, Long Beach, California) | KC309494, KC309567, KC309639 |
| *Eucampia zodiacus* Ehrenberg | HK320 | ECT3896 (Port Aransas Jetty, Texas) | KC309495, KC309568, KC309640 |
| *Eunotia curvata* Lagerstedt | HK086 | FD412 (UTEX) | HQ912599, HQ912463, HQ912292 |
| *Eunotia glacialis* Meister | HK069 | FD46 (UTEX) | HQ912586, HQ912450, HQ912279 |
| *Eunotia pectinalis* (Kützing) Rabenhorst | HK153 | NIES461 (MCC-NIES) | HQ912636, HQ912500, HQ912329 |
| *Eunotia sp.* Ehrenberg | HK286 | ECT3676 (Tinago River, Guam) | KC309480, KC309552, KC309623 |
| *Eunotogramma lunatum* Ashworth | HK294 | ECT3886 (Bald Head Island, North Carolina) | JN975245, JN975259, JN975274 |
| *Eunotogramma lunatum* Ashworth | HK451 | AtlanticPlankton#8 (Atlantic coast, Florida) | MN917237, MN917212, MN917225 |
| *Extubocellulus cribriger* Hasle, von Stosch & Syvertsen | HK046 | CCMP391 | HQ912571, HQ912435, HQ912265 |
| *Extubocellulus cupola* Dąbek, Krzywda & Ch. Li |  | SZCZCH1510 | N/A, MF001955, MF001927 |
| *Extubocellulus cupola* Dąbek, Krzywda & Ch. Li |  | SZCZCH1125 | MF001956, MF001928, MF001981 |
| *Extubocellulus cupola* Dąbek, Krzywda & Ch. Li |  | SZCZP1050 | MF001958, MF001930, MF001982 |
| *Extubocellulus cupola* Dąbek, Krzywda & Ch. Li |  | SZCZP1106 | MF001983, MF001959, MF001931 |
| *Extubocellulus cupola* Dąbek, Krzywda & Ch. Li |  | SZCZP1114 | MF001960, MF001932, MF001984 |
| *Extubocellulus spinifer* (Hargreaves & Guillard) Hasle, Stosch & Syvertsen |  | SZCZCH698 | MF001963, MF001935, MF001985 |
| *Extubocellulus spinifer* (Hargreaves & Guillard) Hasle, Stosch & Syvertsen |  | SZCZCH820 | MF001964, MF001936, MF001986 |
| *Extubocellulus spinifer* (Hargreaves & Guillard) Hasle, Stosch & Syvertsen |  | s0390 | N/A, MF001961, MF001933 |
| *Extubocellulus spinifer* (Hargreaves & Guillard) Hasle, Stosch & Syvertsen |  | s0392 | N/A, MF001962, MF001934 |
| *Extubocellulus* sp Hasle, Stosch & Syvertsen | HK149 | CCAP1018/1 | N/A, KC309570, KC309642 |
| *Fallacia monoculata* (Hustedt) Mann | HK082 | FD254 (UTEX) | HQ912596, HQ912460, HQ912289 |
| *Fallacia pygmaea* (Kützing) Stickle & Mann | HK093 | FD294 (UTEX) | HQ912605, HQ912469, HQ912298 |
| *Fallacia sp.* Stickle & D.G. Mann | HK482 | GU52X-3 (Outhouse Beach, Guam) | MH063467, MH064092, MH064000 |
| *Fistulifera pelliculosa* (Brebisson) Lange-Bertalot |  |  | AY485454, HQ337547, N/A |
| *Fistulifera saprophila* (Lange-Bertalot & Bonik) Lange-Bertalot |  |  | KC736618, KC736593, N/A |
| *Fistulifera solaris* Mayama, Matsumoto, Nemoto & Tanaka |  |  | AB769957, AP011960 |
| *Florella intermedia* (Grunow) Lobban, J.N.Navarro et T.M.Schust. | HK175 | ECT3756 (Guam) | JN975246, JN975260, JN975275 |
| *Fragilariforma virescens* (Ralfs) Williams & Round | HK132 | FD291 (UTEX) | HQ912628, HQ912492, HQ912321 |
| *Gedaniella alfred-wegeneri* Li, Sato & Witkowski |  | s0263 | KR048196, KR048218, KR048230 |
| *Gedaniella boltonii* Li, Krawczyk, Dabek & Witkowski |  | s0393 | MF093083, MF092914, MF093000 |
| *Gedaniella boltonii* Li, Krawczyk, Dabek & Witkowski |  | SZCZCH751 | MF093068, MF092900, MF092986 |
| *Gedaniella boltonii* Li, Krawczyk, Dabek & Witkowski |  | SZCZP10 | MF093073, MF092905, MF092991 |
| *Gedaniella boltonii* Li, Krawczyk, Dabek & Witkowski |  | SZCZP108 | MF093072, MF092904, MF092990 |
| *Gedaniella flavovirens* (Takano) Li, Witkowski & Ashworth |  | CCMP2653 | MF093127, MF093042, MF092958 |
| *Gedaniella flavovirens* (Takano) Li, Witkowski & Ashworth |  | SZCZCH1276 | MF093091, MF092922, MF093008 |
| *Gedaniella flavovirens* (Takano) Li, Witkowski & Ashworth |  | SZCZCH1279 | MF093088, MF092919, MF093005 |
| *Gedaniella flavovirens* (Takano) Li, Witkowski & Ashworth |  | SZCZCH1282 | MF093092, MF092923, MF093009 |
| *Gedaniella flavovirens* (Takano) Li, Witkowski & Ashworth |  | SZCZCH154 | MF093074, MF092906, MF092992 |
| *Gedaniella flavovirens* (Takano) Li, Witkowski & Ashworth |  | SZCZP38 | MF093077, MF092909, MF092995 |
| *Gedaniella mutabilis* Li & Witkowski |  | SZCZCH1381 | MF093117, MF092948, MF093032 |
| *Gedaniella mutabilis* Li & Witkowski |  | SZCZCH153 | MF093069, MF092901, MF092987 |
| *Gedaniella panicellus* Li, Yu & Witkowski |  | SZCZCH1280 | MF093122, MF092953, MF093037 |
| *Gedaniella panicellus* Li, Yu & Witkowski |  | SZCZCH1350 | MF093094, MF092925, MF093011 |
| *Gedaniella panicellus* Li, Yu & Witkowski |  | SZCZCH1379 | MF093132, MF092963, MF093047 |
| *Gedaniella paucistriata* Li & Witkowski |  | SZCZCH1281 | MF093089, MF092920, MF093006 |
| *Geissleria decussis* (Østrup) Lange-Bertalot & Metzeltin |  | FD050 (UTEX) | KJ011647, KJ011830, N/A |
| *Glyphodesmis* sp Greville | HK357 | ECT3891 (St. George Island, Florida) | N/A, JX401248, JX401266 |
| *Gomphonema affine* Kützing | HK098 | FD173 (UTEX) | HQ912608, HQ912472, HQ912301 |
| *Gomphonema parvulum* (Kützing) Kützing | HK081 | FD241 (UTEX) | HQ912595, HQ912459, HQ912288 |
| *Gomphonemopsis* cf *pseudoexigua* (Simonsen) Medlin | UTKSA0026x | SA18 (Duba, Saudi Arabia) | MH063471, MH064098, MH064005 |
| *Grammatophora macilenta* W. Smith | HK368 | GU44AK-4 (Gab Gab Beach, Guam) | JX401241, JX401259, JX401276 |
| *Grammatophora oceanica* Ehrenberg | HK147 | CCMP410 | HQ912634, HQ912498, HQ912327 |
| *Grammatophora* sp Ehrenberg | HK459 | Nate Site 1 (Hawaii) | MG684352, MG684323, MG684295 |
| *Grammatophora* sp Ehrenberg | UTKSA0132 | KSA2015-16 (Al-Nawras, Jeddah, Saudi Arabia) | MH063514, MH064146, MH064051 |
| *Grammatophora undulata* Ehrenberg | HK367 | Coz-3 (Cozumel, Mexico) | JX401240, JX401258, JX401275 |
| *Grammonema striatula* (Lyngbye) Agardh | HK371 | ECT3897 (Pebble Beach, California) | KF701591, KF701600, KF701609 |
| *Grunowago* sp Lobban & Ashworth | HK291 | ECT3874 (Channel #5, Florida Bay, Florida) | JX413548, JX413565, JX413582 |
| *Grunowago pacifica* Lobban & Ashworth | HK679 | GU44AI-5 (Gab Gab Beach, Guam) | N/A, OP297442, OP297499 |
| *Guinardia delicatula* (Cleve) Hasle | HK205 | ECT3821 (Ward Island, Texas) | HQ912651, HQ912515, HQ912344 |
| *Guinardia delicatula* (Cleve) Hasle | HK613 | 16VIII13-1 (Bar Harbor, Maine) | MZ546205, MZ542385, MZ542405 |
| *Guinardia delicatula* (Cleve) Hasle | HK614 | ECT3920 (Ft. Stevens Park, Oregon) | MZ546206, MZ542386, MZ542406 |
| *Guinardia flaccida* (Castracane) H. Peragallo | HK272 | ECT3870 (Boca Chica Channel, Florida Bay, Florida) | KC309535, KC309608, KC309679 |
| *Guinardia flaccida* (Castracane) H. Peragallo | HK353 | ECT3891 (St. George Island, Florida) | KC309536, KC309609, KC309680 |
| *Guinardia striata* (Stolterfoth) Hasle | HK400 | 24IV12-2 (Port O’Connor, Texas) | KJ577857, KJ577894, KJ577928 |
| *Gyrosigma acuminatum* (Kützing) Rabenhorst | HK085 | FD317 (UTEX) | HQ912598, HQ912462, HQ912291 |
| *Halamphora catenulafalsa* Witkowski & Ch. Li | SZCZCH452 |  | KT943646, KT943669, KT943704 |
| *Halamphora coffeaeformis* (Agardh) Levkov | HK089 | FD75 (UTEX) | HQ912602, HQ912466, HQ912295 |
| *Halamphora* *coloradiana* Stepanek & Kociolek | AMPH025 |  | KJ463450, KJ463480, KJ463510 |
| *Halamphora cf costata* (Smith) Levkov | UTKSA0195 | KSA2015-22 (Markaz Al Shoaibah, Saudi Arabia) | MH063468, MH064093, MH064001 |
| *Halamphora montana* (Krasske) Levkov | TCC477 |  | KC736615, KC736590, N/A |
| *Halamphora normanii* (Rabenhorst) Levkov |  |  | AM501958, AM710424, N/A |
| *Halamphora oligotraphenta* (Lange-Bertalot) Levkov | AMPH009 |  | KJ463451, KJ463481, KJ463511 |
| *Halamphora veneta* (Kützing) Levkov | AMPH005 |  | KJ463452, KJ463482, KJ463512 |
| *Hanicella moenia* Lobban & Ashworth | HK379 | GU44AK-6 (Gab Gab Beach, Guam) | KF701599, KF701608, KF701617 |
| *Hantzschia amphioxys v. major* Grunow in Van Heurck |  |  | HQ912404, HQ912390, HQ912376 |
| *Haslea arculata* Lobban & Ashworth | HK605 | ECT3692 (Sala Glula, Guam) | MN977829, MN977806, MN977811 |
| *Haslea arculata* Lobban & Ashworth | HK607 | ECT3692 (Sala Glula, Guam) | MN977830, MN977807, MN977812 |
| *Helicotheca tamensis* (Shrubsole) M.Ricard | HK401 | 25VI12-2A SC (Lady’s Island, South Carolina) | KJ577858, KJ577895, KJ577929 |
| *Hemiaulus sinensis* Greville | HK123 | CCH-1 (Corpus Christi Bay, Texas) | HQ912624, HQ912488, HQ912317 |
| *Hemiaulus sinensis* Greville | HK297 | 24I10-1A (Clear Lake, Texas) | KC309496, KC309569, KC309641 |
| *Hemidiscus cuneiformis* Wallich |  |  | KY362440, N/A, N/A |
| *Hendeyella dimeregrammopsis* Ashworth | HK391 | Coz-1 (Cozumel, Mexico) | KJ577850, KJ577885, KJ577920 |
| *Hendeyella lineata* Ashworth & Lobban | HK325 | GU44AI-3 (Gab Gab Beach, Guam) | JX413547, JX413564, JX413581 |
| *Hendeyella rhombica* Ashworth | HK445 | NateSite1 (Hawaii) | N/A, KU851891, KU851906 |
| *Hendeyella* sp Ashworth, Witkowski & Ch. Li | HK658 | P26 (Biscayne Bay, Florida) | MW327175, MW324598, MW324650 |
| *Hippodonta capitata* (Ehrenberg) Lange-Bertalot, Metzeltin & Witkowski |  |  | AM501966, AM710432, N/A |
| *Hobaniella longicruris* (Greville) P.A. Sims & D.M. Williams | HK226 | ECT3828 (Redfish Bay, Texas) | JX413553, JX413570, JX413587 |
| *Hobaniella* cf *longicruris* (Greville) P.A. Sims & D.M. Williams | HK407 | AtlanticPlankon#8 (Atlantic coast, Florida) | KJ577864, KJ577901, KJ577935 |
| *Hobaniella longicruris* (Greville) P.A. Sims & D.M. Williams | HK576 | 25VI12-1A (Hunting Island, South Carolina) | OP304775, OP297444, OP297501 |
| *Hobaniella longicruris* v *hyalina* (Schröder) P.A. Sims & D.M. Williams | HK284 | ECT3884 (Pacific Grove, California) | KC309512, KC309586, KC309658 |
| *Hobaniella longicruris* v *hyalina* (Schröder) P.A. Sims & D.M. Williams | HK338 | ECT3920 (Ft. Stevens State Park, Oregon) | KC309513, KC309587, KC309659 |
| *Hyalodiscus* sp Ehrenberg | HK201 | ECT3681 (Achang Reef, Guam) | HQ912649, HQ912513, HQ912342 |
| *Hyalodiscus* sp Ehrenberg | HK344 | ECT3898 (Stillwater Cove, California) | KC309537, KC309610, KC309681 |
| *Hyalodiscus stelliger* J.W. Bailey | HK042 | CCMP454 | HQ912567, HQ912431, HQ912261 |
| *Hyalodiscus stelliger* J.W. Bailey | HK615 | BayCut (New Jersey) | MZ546207, MZ542387, MZ542407 |
| *Hyalosynedra al-shareefii* Sabir & Theriot | KSA0100 | SA9 (port area, Jeddah, Saudi Arabia) | MG684362, MG684333, N/A |
| *Hyalosynedra al-shareefii* Sabir & Theriot | UTKSA0253 | KSA2015-3 (Jeddah, Saudi Arabia) | MG684363, MG684334, N/A |
| *Hyalosynedra al-turkii* Sabir & Theriot | UTKSA0076 | SA28 (Jeddah, Saudi Arabia) | MG684364, MG684335, MG684303 |
| *Hyalosynedra* *hyalina* (Grunow) Álvarez-Blanco & S. Blanco | UTKSA0189 | KSA2015-14 (Jeddah, Saudi Arabia) | MG684365, MG684336, MG684304 |
| *Hyalosynedra laevigata* (Grun.) Williams and Round | HK510 | 21IV14-4D (Rabbit Key Basin, Florida) | MG684366, MG684337, MG684305 |
| *Hyalosynedra* *prasadii* Theriot & Ashworth | HK511 | 21IV14-4D (Rabbit Key Basin, Florida) | MG684367, MG684338, MG684306 |
| *Hyalosynedra sublaevigata* Álvarez-Blanco & S. Blanco | HK512 | ECT4119 (San Juan, Puerto Rico) | MG684380, MG684350, MG684320 |
| *Hyalosynedra* sp Williams & Round | HK712 | FLChristine Cc cara (Loggerhead sea turtle, Florida) | OP304776, OP297445, OP297502 |
| *Hydrosera sp.* Wallich | HK274 | TN-CYTX025 (Austin, Texas) | HQ912683, HQ912547, N/A |
| *Hydrosera whampoensis* (A.F.Schwarz) Deby |  | s0300 | MZ546208, MZ542388, MZ542408 |
| *Hydrosilicon* *mitra* Brun | UTKSA0421 | KSA2015-37 (Rabigh, Saudi Arabia) | MH063470, MH064097, N/A |
| *Isthmia minima* Harvey & Bailey | HK275 | CXCL (Guam) | HQ912684, HQ912548, N/A |
| *Koernerella recticostata* (Körner) Ashworth, Lobban & Theriot | HK242 | GU44AB-8 (Gab Gab Beach, Guam) | HM627331, HM627328, HM627325 |
| *Lambertocellus africana* (Dąbek & Witkowski) Dąbek, Witkowski & Ashworth |  | SZCZP74 | KU556402, KU556395 KU556409 |
| *Lampriscus orbiculatus* (Shadboldt) Peragallo & Peragallo | HK125 | ECT3560 (Pete’s Reef, Guam) | HQ912679, HQ912543, KC309643 |
| *Lampriscus shadboltianus* v *crenulata* Navarro | HK257 | GU44AB-8 (Gab Gab Beach Guam) | HQ912680, HQ912544, N/A |
| *Lampriscus shadboltianus* (Greville) Peragallo & Peragallo | HK264 | Pan-1 (STRI Station, Panama) | KC309497, KC309571, KC309644 |
| *Lemnicola hungarica* (Grunow) Round | HK129 | FD456 (UTEX) | HQ912626, HQ912490, HQ912319 |
| *Leptocylindrus aporus* (French & Hargraves) Nanjappa & Zingone |  | SZN-B651 | KC814836, KC814841, KC814846 |
| *Leptocylindrus convexa* Nanjappa & Zingone |  | SZN-B768 | KC814839, KC814845, KC814847 |
| *Leptocylindrus danicus* Schutt |  | SZN-B650 | KC894150, KC814842, KC814848 |
| *Leptocylindrus danicus* Cleve | HK238 | ECT3845 (Ward Island, Texas) | JX413558, JX413575, JX413592 |
| *Leptocylindrus hargravesii* Nanjappa & Zingone |  | CCMP1856 | KC894151, N/A, KC814849 |
| *Leptocylindrus hargravesii* Nanjappa & Zingone |  | SZNB781 | KC814809, KC814843, KC894145 |
| *Leptocylindrus minimus* Gran | HK355 | ECT3920 (Ft. Stevens State Park, Oregon) | KC309538, N/A, N/A |
| *Leptocylindrus danicus* Cleve | HK608 | Tong Yeong Station (South Korea) | MZ546209, MZ542389, MZ542409 |
| *Leyanella arenaria* Hasle, von Stosch & Syvertsen | HK045 | CCMP471 | HQ912570, HQ912434, HQ912264 |
| *Leyanella arenaria* Hasle, von Stosch & Syvertsen |  | SZCZCH1529 | MF001966, MF001938, MF001987 |
| *Leyanella arenaria* Hasle, von Stosch & Syvertsen |  | SZCZM1156 | N/A, MF001965, MF001937 |
| *Leyanella arenaria* Hasle, von Stosch & Syvertsen |  | s0389 | N/A, MF001967, MF001939 |
| *Leyanella pauciporis* Ashworth, Dąbek & Park |  | Tong Yeong Station (South Korea) | KU556405, KU556398, KU556412 |
| *Leyanella probus* Ashworth, Dąbek & Witkowski |  | SZCZP588 | KU556404, KU556397, KU556411 |
| *Leyanella probus* Ashworth, Dąbek & Witkowski | HK708 | ECT3888 (Canary Islands) | KU556403, KU556396, KU556410 |
| *Leyanella probus* Ashworth, Dąbek & Witkowski | HK709 | ECT3888 (Canary Islands) | OP304777, OP297446, N/A |
| *Leyanella probus* Ashworth, Dąbek & Witkowski | HK710 | Azo42a (Azores) | OP304778, OP297447, OP297503 |
| *Licmophora abbreviata* Agardh | UTKSA0049 | SA29 (Jeddah, Saudi Arabia) | KP125882, KP125883, KP125884 |
| *Licmophora colosalis* Belando, Aboal & Jiménez | HK366 | ECT3907 (Rabbit Key Basin, Florida) | JX401239, JX401257, JX401274 |
| *Licmophora colosalis* Belando, Aboal & Jiménez | UTKSA0066 | SA29 (Jeddah, Saudi Arabia) | MG684358, MG684329, MG684299 |
| *Licmophora curvata* Lobban, Tharngan & Ashworth | HK422 | GU44AY-6 (Gab Gab Beach, Guam) | KP125877, KP125880, N/A |
| *Licmophora aff ehrenbergii* (Kützing) Grunow | HK420 | GU7X-6 (University of Guam Marine Lab, Guam) | KP125876, KP125879, KP125881 |
| *Licmophora flucticulata* Lobban, Schefter & Ruck |  | GU56-A (Cocos Wall, Guam) | HQ997923, JN975262, JN975277 |
| *Licmophora normaniana* (Greville) Wahrer | HK403 | 26II12-1 TX (Mustang Island, Texas) | KJ577860, KJ577897. KJ577931 |
| *Licmophora normaniana* (Greville) Wahrer in Wahrer, Fryxell & Cox | HK528 | ChelMyN (Turtle Hospital, Marathon, Florida) | MH040310, MH040256, MH040231 |
| *Licmophora paradoxa* (Lyngbye) Agardh | HK106 | CCMP2313 | HQ912612, HQ912476, HQ912305 |
| *Licmophora peragallioides* (Lobban) Lobban & Ashworth | HK364 | GU44AL-3 (Gab Gab Beach, Guam) | JX401237, JX401255, JX401273 |
| *Licmophora* sp Agardh | HK302 | GU52-O (Outhouse Beach, Guam) | JN975248, JN975263, N/A |
| *Licmophora* sp Agardh | HK365 | Coz2 (Cozumel, Mexico) | JX401238, JX401256, N/A |
| *Licmophora* sp Agardh | HK455 | 24IV14-3A (Pickles Reef, Florida) | MH040311, MH040257, MH040232 |
| *Licmophora* sp Agardh | HK456 | 24IV14-3A (Pickles Reef, Florida) | MH040312, MH040258, MH040233 |
| *Licmophora* sp Agardh | HK522 | GU7AC-1 (University of Guam Marine Lab, Guam) | MH040313, MH040259, MH040234 |
| *Licmophora* sp Agardh | HK523 | GU7AC-1 (University of Guam Marine Lab, Guam) | MH040314, MH040260, N/A |
| *Licmophora* sp Agardh | HK525 | GU44BR-5 (Gab Gab Beach, Guam) | MH040316, MH040262, MH040235 |
| *Licmophora* sp Agardh | HK526 | GU44BR-5 (Gab Gab Beach, Guam) | MH040317, MH040263, MH040236 |
| *Licmophora* sp Agardh | HK527 | GU44BR-5 (Gab Gab Beach, Guam) | MH040318, MH040264, MH040237 |
| *Licmophora* sp Agardh | HK650 | GU7X-6 (University of Guam Marine Lab, Guam) | MW327177, MW324601, MW324652 |
| *Licmophora* sp Agardh | HK716 | GU52AB (Outhouse Beach, Guam) | OP304779, OP297448, OP297504 |
| *Licmophora* sp Agardh | KSA0085 | SA4 (Durrah, Saudi Arabia) | MG684353, MG684324, N/A |
| *Licmophora* sp Agardh | KSA0151 | SA1 (Durrah, Saudi Arabia) | MG684354, MG684325, N/A |
| *Licmophora* sp Agardh | UTKSA0010 | SA29 (Jeddah, Saudi Arabia) | MG684355, MG684326, MG684296 |
| *Licmophora* sp Agardh | UTKSA0029 | SA18 (Duba, Saudi Arabia) | MG684356, MG684327, MG684297 |
| *Licmophora* sp Agardh | UTKSA0050 | SA18 (Duba, Saudi Arabia) | MG684357, MG684328, MG684298 |
| *Licmophora* sp Agardh | UTKSA0084 | SA18 (Duba, Saudi Arabia) | MG684359, MG684330, MG684300 |
| *Licmophora* sp Agardh | UTKSA0191 | KSA2015-14 (Bhadur Resort, Saudi Arabia) | MH063515, MH064147, MH064052 |
| *Lithodesmioides polymorpha* von Stosch | HK211 | ECT3772 (Taeleyag Beach, Guam) | HQ912655, HQ912519, HQ912348 |
| *Lithodesmium intricatum* (T. West) Peragallo & Peragallo | HK231 | ECT3836 (Rainbow Harbor, Long Beach, California) | HQ912670, HQ912534, HQ912362 |
| *Lithodesmium intricatum* (T. West) Peragallo & Peragallo | HK253 | ECT3850 (Kahalu’u, Hawaii) | HQ912678, HQ912542, HQ912368 |
| *Lithodesmium intricatum* (T. West) Peragallo & Peragallo | HK586 | 25VI12-1A (Hunting Island, South Carolina) | N/A, MK817349, MZ542410 |
| *Lithodesmium undulatum* Ehrenberg | HK029 | CCMP1797 | HQ912559, HQ912423, HQ912253 |
| *Lucanicum concatenatum* Lobban & Ashworth | HK378 | GU44AI-3 (Gab Gab Beach, Guam) | KF701598, KF701607, KF701616 |
| *Luticola goeppertiana* (Bleisch) D.G.Mann ex J.Rarick, S.Wu, S.S.Lee & Edlund |  |  | AM501967, AM710433, N/A |
| *Mastodiscus radiatus* Prasad & Nienow | HK249 | ECT3822 (Ward Island, Texas) | HQ912675, HQ912539, HQ912366 |
| *Mastogloia* *aquilegiae* Grunow in Moller | UTKSA0224 | KSA2015-49 (Duba, Saudi Arabia) | N/A, MH064100, MH064007 |
| *Mastogloia fimbriata* (T. Brightwell) Grunow | HK485 | GU52X-1 (Outhouse Beach, Guam) | MH040321, MH040270, MH040243 |
| *Mastogloia* cf *pumila* (Grunow) Cleve | HK136 | 29X07-6B (Mustang Island, Texas) | HQ912632, HQ912496, HQ912325 |
| *Mastogloia* sp Thwaites in W. Smith | HK314 | ECT3762 (Taeleyag Beach, Guam) | KC309481, KC309553, N/A |
| *Mastogloia sp.* Thwaites in W. Smith | HK718 | 11IV18-2A (Long Lake, Florida) | OP304780, OP297449, OP297505 |
| *Mastogloia* sp Thwaites in W. Smith | KSA0062 | SA17 (Duba, Saudi Arabia) | MH063473, MH064101, MH064008 |
| *Mastogloia* sp Thwaites in W. Smith | UTKSA0313 | KSA0216-44 (Markaz Al Shoaibah, Saudi Arabia) | MH063474, MH064102, MH064009 |
| *Mayamea perimitis* (Hustedt) K. Bruder & L.K. Medlin | TCC540 |  | KC736630, KC736600, N/A |
| *Melosira arctica* v *krembsii* Kaczmarska et R. Jahn | HK425 | AKIce (Barrow, Alaska) | MZ546210, MZ542390, MZ542411 |
| *Melosira nummuloides* (Agardh) Greville | HK041 | CCMP482 | HQ912566, HQ912430, HQ912260 |
| *Melosira* sp Agardh | HK404 | BayCut (New Jersey) | KJ577861, KJ577898, KJ577932 |
| *Melosira* sp Agardh | HK616 | GU7X-5 (UoG Marine Laboratory, Guam) | MZ546211, MZ542391, N/A |
| *Melosira* sp Agardh | UTKSA0274 | KSA2015-16 (Al-Nawras, Jeddah, Saudi Arabia) | OP304781, OP297450, OP297506 |
| *Melosira varians* Agardh | HK266 | ECT3833 (Corona Del Mar, California) | KC309539, KC309611, KC309682 |
| *Melosira varians* Agardh | HK617 | 8VII12-1 (Cleveland, Ohio) | MZ546212, MZ542392, MZ542412 |
| *Meuniera membranacea* (Cleve) P. C. Silva | HK313 | ECT3896 (Port Aransas Jetty, Texas) | KC309482, KC309554, KC309624 |
| *Microtabella interrupta* Ehrenberg | HK248 | ECT3700 (Gab Gab Beach, Guam) | JN975247, JN975261, JN975276 |
| *Microtabella interrupta* Ehrenberg | HK458 | 20X15-1 (Boca Chica Channel, Florida) | MH040319, MH040265, MH040238 |
| *Minidiscus trioculatus* (F. J. R. Taylor) Hasle | HK036 | CCMP495 | HQ912563, HQ912427, HQ912257 |
| *Minutocellus polymorphus* (Hargraves & Guillard) Hasle, von Stosch & Syvertsen | HK043 | CCMP497 | HQ912568, HQ912432, HQ912262 |
| *Minutocellus polymorphus* (Hargraves & Guillard) Hasle, von Stosch & Syvertsen | HK321 | ECT3920 (Ft. Stevens State Park, Oregon) | KC309498, KC309572, KC309645 |
| *Minutocellus polymorphus* (Hargraves & Guillard) Hasle, von Stosch & Syvertsen |  | SZCZP72 | MF001988, MF001968, MF001940 |
| *Minutocellus* sp Hasle, von Stosch & Syvertsen | HK519 | P1 12-8-13 (Pensacola Bay, Florida) | OP304782, OP297451, OP297507 |
| *Nanofrustulum shiloi* (J.J. Lee, C.W. Reimer, & M.E. McEnery) F.E. Round, H. Hallsteinsen, & E. Paasche |  | CCMP1306 | MF093126, MF092957, MF093041 |
| *Nanofrustulum* cf *shiloi* (J.J. Lee, C.W. Reimer, & M.E. McEnery) F.E. Round, H. Hallsteinsen, & E. Paasche | HK056 | CCMP2649 | HQ912578, HQ912442, HQ912271 |
| *Nanofrustulum shiloi* (J.J. Lee, C.W. Reimer, & M.E. McEnery) F.E. Round, H. Hallsteinsen, & E. Paasche |  | SZCZE1361 | MF093128, MF092959, MF093043 |
| *Nanofrustulum shiloi* (J.J. Lee, C.W. Reimer, & M.E. McEnery) F.E. Round, H. Hallsteinsen, & E. Paasche |  | SZCZE381 | MF093100, MF092931, MF093017 |
| *Nanofrustulum shiloi* (J.J. Lee, C.W. Reimer, & M.E. McEnery) F.E. Round, H. Hallsteinsen, & E. Paasche |  | SZCZM115 | MF093099, MF092930, MF093016 |
| *Nanofrustulum shiloi* (J.J. Lee, C.W. Reimer, & M.E. McEnery) F.E. Round, H. Hallsteinsen, & E. Paasche |  | SZCZM1342 | MF093118, MF092949, MF093033 |
| *Nanofrustulum shiloi* (J.J. Lee, C.W. Reimer, & M.E. McEnery) F.E. Round, H. Hallsteinsen, & E. Paasche |  | SZCZM404 | MF093101, MF092932, MF093018 |
| *Nanofrustulum shiloi* (J.J. Lee, C.W. Reimer, & M.E. McEnery) F.E. Round, H. Hallsteinsen, & E. Paasche |  | SZCZP1040 | MF093104, MF092935, MF093020 |
| *Nanofrustulum* sp F.E. Round, H. Hallsteinsen, & E. Paasche | KSA0090 | SA9 (Jeddah, Saudi Arabia) | OP304783, OP297452, OP297508 |
| *Nanofrustulum wachnickianum* Li, Witkowski & Ashworth |  | SZCZCH193 | MF093064, MF092896, MF092982 |
| *Navicula cari* Ehrenberg |  |  | AM501991, AM710457, N/A |
| *Navicula cryptocephala* Kützing | HK090 | FD109 (UTEX) | HQ912603, HQ912467, HQ912296 |
| *Navicula hippodontofallax* Witkowski & Ch. Li |  | SZCZCH703 | KT943636, KT943661, KT943695 |
| *Navicula perminuta* Østrup | mbccc3 |  | JQ045340, JQ432375, N/A |
| *Navicula* *perminuta* Østrup | HK561 | FLMan10 (Manatee, Crystal River, Florida) | N/A, MT432484, MT432502 |
| *Navicula reinhardtii* Grunow in Cleve & Möller |  |  | AM501976, AM710442, N/A |
| *Navicula* sp Bory | HK486 | Coz4 (Cozumel, Mexico) | MH040322, MH040271, MH040244 |
| *Navicula* sp Bory | HK488 | 24IV14-2A (Conch Reef, Florida) | MH063475, MH064104, MH064011 |
| *Navicula* sp Bory | HK489 | 24IV14-3A (Pickles Reef, Florida) | MH063476, MH064105, MH064012 |
| *Navicula* sp Bory | HK491 | 17VIII13-2 (Belfast, Maine) | MT441512, MT432483, MT432503 |
| *Navicula* sp Bory | HK493 | GU52X-1 (Outhouse Beach, Guam) | N/A, MH064095, MH064003 |
| *Navicula* sp Bory | HK494 | GU7Y-4 (University of Guam Marine Labs, Guam) | N/A, MH040268, MH040241 |
| *Navicula* sp Bory | HK496 | PR6 (San Juan, Puerto Rico) | MH017640, MH040269, MH040242 |
| *Navicula* sp Bory | HK500 | CGA1605-D (Manatee, Georgia) | MH017641, MN977810, MN977815 |
| *Navicula* sp Bory | HK558 | FLMan1 (Manatee, Crystal River, Florida) | N/A, MN977809, MN977814 |
| *Navicula* sp Bory | HK559 | FLMan1 (Manatee, Crystal River, Florida) | MN977831, MN977808, MN977813 |
| *Navicula* sp Bory | KSA0102 | SA4 (Durrah, Saudi Arabia) | KX981844, KX981821, KX981797 |
| *Navicula* sp Bory | KSA0112 | SA23 (Al-Wajh, Saudi Arabia) | N/A, MH064106, MH064013 |
| *Navicula* sp Bory | UTKSA0122 | KSA2015-30 (Markaz Al Shoaibah, Saudi Arabia) | N/A, MH064096, MH064004 |
| *Navicula* sp Bory | UTKSA0131 | KSA2015-19 (Al-Nawras, Jeddah, Saudi Arabia) | MH063477, MH064107, MH064014 |
| *Navicula* sp Bory | UTKSA0162 | KSA2015-14 (Bhadur Resort, Saudi Arabia) | MH063478, MH064108, MH064015 |
| *Navicula* sp Bory | UTKSA0239 | KSA2015-41 (Rabigh, Saudi Arabia) | MH063479, MH064109, MH064016 |
| *Navicula tripunctata* (O.F. Müller) Bory |  |  | AM502028, AM710495, N/A |
| *Navicula zhengii* Witkowski & Li | SZCZCH96 |  | KT943632, KT943681, KT943691 |
| *Neidium affine* (Ehrenberg) Pfitzer | HK064 | FD127 (UTEX) | HQ912583, HQ912447, HQ912276 |
| *Neidium bisulcatum* (Lagerstedt) Cleve | HK076 | FD417 (UTEX) | HQ912591, HQ912455, HQ912284 |
| *Neidium productum* (W. Smith) Cleve | HK063 | FD116 (UTEX) | HQ912582, HQ912446, HQ912275 |
| *Neocalyptrella robusta* (G.Norman ex Ralfs) Hernández-Becerril & Meave del Castillo | HK423 | P1 12-8-13 (Pensacola Bay, Florida) | MN917245, MN917222, N/A |
| *Neodelphineis* sp*.* Takano | HK421 | FijiBottleNY (New York) | KP125875, KP125878, N/A |
| *Neodelphineis* sp*.* Takano | HK661 | TXOffshore 7/25/18 (Gulf of Mexico) | MW327179, MW324603, MW324654 |
| *Neofragilaria montgomeryii* Ashworth & Ch. Li | HK521 | DTort11 (Dry Tortugas, Florida) | MN473924, MN473941, MN473966 |
| *Neofragilaria nicobarica* Desikachary, Prasad & Prema |  | s0371 | AB433340, KR048216, KR048228 |
| *Neofragilaria* cf *nicobarica* Desikachary, Prasad & Prema | HK375 | Coz-1 (Cozumel, Mexico) | KF701595, KF701604, KF701613 |
| *Neofragilaria stilus* Krzywda, Witkowski & Ch. Li |  | SZCZM116 | KR048193, KR048213, KR048225 |
| *Neostreptotheca* sp von Stosch | UTKSA0005 | SA12 (Markaz Al Shoaibah, Saudi Arabia) | OP304784, OP297453, OP297509 |
| *Neosynedra* *provincialis* (Grunow) Williams & Round | HK457 | 24IV14-3A (Pickles Reef, Florida) | N/A, MH040266, MH040239 |
| *Nitzschia acidoclinata* Lange-Bertalot |  |  | KC736632, KC736602, N/A |
| *Nitzschia asteropeae* Lobban, Ashworth & Calaor | HK467 | GU52V-2 (Outhouse Beach, Guam) | MW327180, MW324605, MW324656 |
| *Nitzschia aurariae* Cholnoky | SZCZCH966 |  | KT943639, KT943663, KT943698 |
| *Nitzschia celaenoae* Lobban, Ashworth, Calaor & Theriot | KSA0035 | SA4 (Durrah, Saudi Arabia) | KU179128, KU179116, KU179143 |
| *Nitzschia draveillensis* Coste & Ricard |  |  | KC736635, KC736605, N/A |
| *Nitzschia dubiformis* Hustedt |  |  | AB430616, AB430696, N/A |
| *Nitzschia inconspicua* Grunow |  |  | KC736636, KC736607, N/A |
| *Nitzschia filiformis* (W. Smith) Van Heurck | HK073 | FD267 (UTEX) | HQ912589, HQ912453, HQ912282 |
| *Nitzschia* cf *frigida* Grunow | HK468 | AKIce (Barrow, Alaska) | N/A, MH064110, MH064017 |
| *Nitzschia* cf *longissima* (Brébisson in Kützing) Grunow | HK176 | ECT3689 (Sala Glula, Guam) | KX981850, KX981829, KX981804 |
| *Nitzschia* cf *longissima* (Brébisson in Kützing) Grunow | UTKSA0021 | SA29 (Jeddah, Saudi Arabia) | MH063480, MH064111, MH064018 |
| *Nitzschia* cf *longissima* (Brébisson in Kützing) Grunow | UTKSA0124 | KSA2015-9 (Bhadur Resort, Saudi Arabia) | MH063481, MH064112, MH064019 |
| *Nitzschia longissima* (Brébisson in Kützing) Grunow |  |  | AY881968, AY881967, N/A |
| *Nitzschia lorenziana* Grunow |  |  | KC736637, KC736608, N/A |
| *Nitzschia martiana* (C. Agardh) Van Heurck | HK405 | 3VIII07 (Talofofo Bay, Guam) | N/A, KJ577899, KJ577933 |
| *Nitzschia schefterae* Lobban, Ashworth & Calaor | HK464 | 19X15-1B (US-1 Channel #5, Florida) | MW327181, MW324606, MW324657 |
| *Nitzschia schefterae* Lobban, Ashworth & Calaor | HK465 | GU52X-1 (Outhouse Beach, Guam) | MW327182, MW324607, MW324658 |
| *Nitzschia* sp Hassall | HK469 | Rincon Mangrove (Costa Rica) | MH040323, MH040273, MH040246 |
| *Nitzschia* sp Hassall | HK470 | Nate Site 1 (Kona, Hawaii) | MH040324, MH040274, MH040247 |
| *Nitzschia* sp Hassall | HK472 | Coz4 (Cozumel, Mexico) | MH040325, N/A, MH040248 |
| *Nitzschia* sp Hassall | HK473 | GU52X-4 (Outhouse Beach, Guam) | MH040326, MH040275, MH040249 |
| *Nitzschia* sp Hassall | HK476 | GU52X-4 (Outhouse Beach, Guam) | OP304785, OP297454, OP297510 |
| *Nitzschia* sp Hassall | HK533 | Tokiane4 (Madagascar) | MW327183, MW324608, MW324659 |
| *Nitzschia* sp Hassall | HK534 | Tong Yeong St (South Korea) | MW327184, MW324609, N/A |
| *Nitzschia* sp Hassall | HK535 | Coz4 (Cozumel, Mexico) | N/A, MW324610, MW324660 |
| *Nitzschia* sp Hassall | HK536 | Coz4 (Cozumel, Mexico) | MW327185, MW324611, MW324661 |
| *Nitzschia* sp Hassall | HK562 | FLMan10 (manatee, Crystal River, Florida) | OP184789, OP191786, OP191831 |
| *Nitzschia* sp Hassall | HK675 | CCMP1698 | MW327188, MW324614, MW324664 |
| *Nitzschia* sp Hassall |  | SZCZCH658 | KT943651, KT943676, KT943713 |
| *Nitzschia* sp Hassall | UTKSA0053 | SA19 (Al-Wajh, Saudi Arabia) | N/A, MH064113, MH064020 |
| *Nitzschia* sp Hassall | UTKSA0102 | KSA2015-14 (Bhadur Resort, Saudi Arabia) | MH063482, MH064114, MH064021 |
| *Nitzschia* sp Hassall | UTKSA0106 | KSA2015-49 (Duba, Saudi Arabia) | MH063483, MH064115, MH064022 |
| *Nitzschia* sp Hassall | UTKSA0107 | KSA2015-49 (Duba, Saudi Arabia) | MH063484, MH064116, MH064023 |
| *Nitzschia* sp Hassall | UTKSA0109 | KSA2015-16 (Al-Nawras, Jeddah, Saudi Arabia) | MH063485, MH064117, N/A |
| *Nitzschia* sp Hassall | UTKSA0111 | KSA2015-23 (Markaz Al Shoaibah, Saudi Arabia) | MH063486, MH064118, MH064024 |
| *Nitzschia* sp Hassall | UTKSA0133 | KSA2015-19 (Al-Nawras, Jeddah, Saudi Arabia) | N/A, MW324616, N/A |
| *Nitzschia* sp Hassall | UTKSA0143 | KSA2015-11 (Bhadur resort, Jeddah, Saudi Arabia) | N/A, MW324617, N/A |
| *Nitzschia* sp Hassall | UTKSA0171 | KSA2015-11 (Bhadur Resort, Saudi Arabia) | MH063487, MH064119, MH064025 |
| *Nitzschia* sp Hassall | UTKSA0173 | KSA2015-37 (Rabigh, Saudi Arabia) | MH063488, MH064120, MH064026 |
| *Nitzschia* sp Hassall | UTKSA0182 | KSA2015-38 (Rabigh, Saudi Arabia) | MH063489, MH064121, MH064027 |
| *Nitzschia* sp Hassall | UTKSA0260 | KSA2015-11 (Bhadur Resort, Saudi Arabia) | MH063490, MH064122, MH064028 |
| *Nitzschia* sp Hassall | UTKSA0326 | KSA2016-5 (Bhadur resort, Jeddah, Saudi Arabia) | N/A, OP297455, OP297511 |
| *Nitzschia taygeteae* Lobban, Ashworth & Calaor | HK466 | GU52X-1 (Outhouse Beach, Guam) | MW327189, MW324615, MW324665 |
| *Nitzschia traheaformis* Ch. Li, Witkowski & Yu Sh. |  | SZCZCH970 | KT943642, KT943666, KT943701 |
| *Nitzschia traheaformis* Ch. Li, Witkowski & Yu Sh. |  | SZCZCH971 | KT943643, KT943667, KT943702 |
| *Nitzschia volvendirostrata* Ashworth, Dabek & Witkowski | HK673 | CCMP2144 | MW327186, MW324612, MW324662 |
| *Nitzschia volvendirostrata* Ashworth, Dabek & Witkowski | HK674 | CCMP2177 | MW327187, MW324613, MW324663 |
| *Nitzschia volvendirostrata* Ashworth, Dabek & Witkowski | KSA0039 | SA12 (Markaz Al Shoaibah, Saudi Arabia) | N/A, KU179112, KU179139 |
| *Odontella aurita* Agardh | HK203 | ECT3619 (Elkhorn Slough, California) | JX413551, JX413568, JX413585 |
| *Odontella aurita* Agardh | HK277 | ECT3788 (Talofofo Bay, Guam) | HQ912686, HQ912550, HQ912372 |
| *Odontella aurita* Agardh | HK278 | ECT3772 (Taelayag Beach, Guam) | HQ912687, HQ912551, HQ912373 |
| *Odontella aurita* Agardh | HK333 | ECT3888 (San Sebastian, Canary Islands) | KC309508, KC309582, KC309655 |
| *Odontella aurita* Agardh | HK334 | ECT3788 (Talofofo Bay, Guam) | KC309509, KC309583, KC309656 |
| *Odontella aurita* Agardh | HK579 | Azo42a (Azores) | OP304787, OP297457, N/A |
| *Odontella aurita* Agardh | HK580 | NateBlackSand (Hawaii) | N/A, OP297458, N/A |
| *Odontella obtusa* Kützing | HK279 | ECT3743 (Talofofo Bay, Guam) | HQ912688, HQ912552, HQ912374 |
| *Odontella obtusa* Kützing | HK578 | OsoBayXing (Corpus Christi, Texas) | OP304786, OP297456, OP297512 |
| *Odontella rhomboides* Jahn & Kusber | HK282 | ECT3845 (Ward Island, Texas) | KC309514, KC309588, KC309660 |
| *Odontella rhomboides* Jahn & Kusber | HK339 | ECT3905 (Florida Bay, Florida) | KC309515, KC309589, KC309661 |
| *Odontella rhomboides* Jahn & Kusber | HK577 | 25VI12-1A (Hunting Island, South Carolina) | OP304788, OP297459, OP297513 |
| *Odontella rhombus* f *trigona* (Cleve ex Van Heurck) R. Ross | HK340 | ECT3891 (St. George Island, Florida) | KC309516, KC309590, KC309662 |
| *Odontella rhombus* f *trigona* (Cleve ex Van Heurck) R. Ross | HK704 | AransasJetty (Port Aransas, Texas) | OP304789, OP297460, OP297514 |
| *Odontella rostrata* (Hustedt) Simonsen | HK225 | ECT3828 (Redfish Bay, Texas) | JX413552, JX413569, JX413586 |
| *Odontella rostrata* (Hustedt) Simonsen | HK585 | AtlanticPlankton#8 (Atlantic coast, Florida) | OP304790, OP297461, OP297515 |
| *Odontella* sp Agardh | HK335 | ECT3854 (Kahana Beach, Hawaii) | KC309517, KC309591, KC309663 |
| *Opephora pacifica* (Grunow) Petit | HK296 | ECT3831 (Texas) | JN975249, JN975264, JN975278 |
| *Palmerina hardmanniana* (Greville) Hasle | HK233 | ECT3847 (Port Aransas ferry crossing, Texas) | HQ912671, HQ912535, HQ912363 |
| *Papiliocellulus simplex* Gardner & Crawford | HK134 | CS431 (CSIRO) | HQ912630, HQ912494, HQ912323 |
| *Paralia longispina* S. Konno & R.W. Jordan | HK408 | GU44AK-4 (Gab Gab Beach, Guam) | KJ577865, KJ577902, KJ577936 |
| *Paralia* sp Heiberg | HK618 | NateSite1 (Kona, Hawaii) | MZ546213, MZ542393, MZ542413 |
| *Paralia sulcata* (Ehrenberg) Cleve | HK048 | CCAP1059/1 | HQ912573, HQ912437, HQ912266 |
| *Parlibellus hamulifer* (Grunow) Cox | HK409 | GU44AK-4 (Gab Gab Beach, Guam) | KJ577866, KJ577903, KJ577937 |
| *Parlibellus harffianus* Witkowski, Ch. Li & S.-X.Yu |  | SZCZCH75 | KT943652, KT943686, KT943715 |
| *Parlibellus* sp Cox | HK428 | SantaRosaCor.green (Costa Rica) | KU179137, KU179122, KU179149 |
| *Parlibellus* sp Cox | UTKSA0240 | KSA2015-41 (Rabigh, Saudi Arabia) | N/A, MW324619, N/A |
| *Perideraion elongatum* R.W. Jordan, Y. Arai & Lobban | HK411 | GU44AK-6 (Gab Gab Beach, Guam) | KJ577868, KJ577905, KJ577939 |
| *Perideraion* cf *elongatum* R.W. Jordan, Y. Arai & Lobban | UTKSA0259 | KSA2015-49 (Duba, Saudi Arabia) | MH063516, MH064148, MH064053 |
| *Perideraion montgomeryii* Lobban, Jordan & Ashworth | HK246 | GU7 (UOG Marine Lab, Guam) | HM627332, HM627329, HM627326 |
| *Phaeodactylum tricornutum* Bohlin | HK011 | CCMP2561 (NCMA) | HQ912556, HQ912420, HQ912250 |
| *Phaeodactylum tricornutum* Bohlin | HK538 | UTEX640 (UTEX) | MH063492, MH064125, MH064031 |
| *Phaeodactylum tricornutum* Bohlin | HK539 | UTEX646 (UTEX) | MH063493, MH064126, MH064032 |
| *Phaeodactylum tricornutum* Bohlin | HK540 | UTEX2089 (UTEX) | MH063494, MH064127, MH064033 |
| *Pinnularia brebissonii* (Kützing) Rabenhorst | HK092 | FD274 (UTEX) | HQ912604, HQ912468, HQ912297 |
| *Pinnularia* sp Ehrenberg | KSA0127 | SA12 (Markaz Al Shoaibah, Saudi Arabia) | KU179135, KU179125, N/A |
| *Pinnularia termitina* (Ehrenberg) Patrick | HK088 | FD484 (UTEX) | HQ912601, HQ912465, HQ912294 |
| *Placoneis elginensis* (Gregory) Cox | HK096 | FD416 (UTEX) | HQ912607, HQ912471, HQ912300 |
| *Plagiogramma porcipellis* Ashworth & Li | HK212 | ECT3776 (Taeleyag Beach, Guam) | HQ912656, HQ912520, HQ912349 |
| *Plagiogramma* sp Greville | HK324 | ECT3924 (Potlatch State Park, Washington) | JX413546, JX413563, JX413580 |
| *Plagiogramma* sp Greville | HK374 | 25VI12-1C (Hunting Island, South Carolina) | KF701594, KF701603, KF701612 |
| *Plagiogramma* sp Greville | HK410 | 25VI12-1C SC (Hunting Island, South Carolina) | KJ577867, KJ577904, KJ577938 |
| *Plagiogramma* sp Greville | HK660 | 14IV18-2B (Carrabelle, Florida) | MW327193, MW324623, N/A |
| *Plagiogrammopsis castigatus* Dabek, Górecka & Witkowski |  | SZCZE471 | MF001995, MF001976, MF001946 |
| *Plagiogrammopsis castigatus* Dabek, Górecka & Witkowski |  | SZCZP1005 | MF001990, MF001970, MF001942 |
| *Plagiogrammopsis castigatus* Dabek, Górecka & Witkowski |  | SZCZP909 | MF001991, MF001971, MF001943 |
| *Plagiogrammopsis* sp Hasle, von Stosch & Syvertsen | HK412 | WhiskeyCreek4 (Whiskey Creek, Florida) | KJ577869, KJ577906, KJ577940 |
| *Plagiogrammopsis van heurckii* (Grunow) Hasle, von Stosch & Syvertsen | HK293 | ECT3885 (Elkhorn Slough, California) | KC309504, KC309578, KC309651 |
| *Plagiogrammopsis van heurckii* (Grunow) Hasle, von Stosch & Syvertsen | HK413 | ECT3856 (Kahana Bay, Hawaii) | KJ577870, KJ577907, KJ577941 |
| *Plagiostriata baltica* Ch. Li, Witkowski & Witak |  | SZCZCH1550 | MF093134, MF092965, MF093049 |
| *Plagiostriata goreensis* Sato & Medlin |  | s0388 | KR048198, KR048220, KR048232 |
| *Plagiotropis* sp Pfitzer | HK508 | PR5 (Condado Lagoon, Puerto Rico) | MH063495, MH064128, MH064034 |
| *Planktoniella sol* (Wallich) Schütt | HK035 | CCMP1608 | HQ912562, HQ912426, HQ912256 |
| *Planothidium frequentissimum* (Lange-Bertalot) Lange-Bertalot |  | PF1 | KJ658409, KJ658392, N/A |
| *Planothidium lanceolatum* (Brébisson ex Kützing) Lange-Bertalot |  | PL2 | KJ658410, KJ658393, N/A |
| *Planothidium* sp Round & Bukhtiyarova |  | SZCZCH26 | KT943653, KT943678, KT943716 |
| *Pleurosigma simulacrum* Lobban & Sterrenburg | HK495 | GU52X-1 (Outhouse Beach, Guam) | MH040327, MH040276, MH040250 |
| *Pleurosigma* sp W. Smith | UTKSA0019 | SA18 (Duba, Saudi Arabia) | KX981840, KX981822, KX981798 |
| *Pleurosigma* sp W. Smith | UTKSA0167 | KSA2015-49 (Duba, Saudi Arabia) | MH063496, MH064129, MH064035 |
| *Pleurosigma* sp W. Smith | UTKSA0264 | KSA2015-16 (Al-Nawras, Jeddah, Saudi Arabia) | MH063497, N/A, MH064036 |
| *Pleurosigma* sp W. Smith | UTKSA0273 | KSA2015-16 (Al-Nawras, Jeddah, Saudi Arabia) | MH063498, MH064130, MH064037 |
| *Pleurosira laevis* (Ehrenberg) Compère | HK068 | FD482 (UTEX) | HQ912585, HQ912449, HQ912278 |
| *Pleurosira laevis* (Ehrenberg) Compère | HK326 | ECT3833 (Corona del Mar, California) | KC309505, KC309579, KC309652 |
| *Pleurosira laevis* (Ehrenberg) Compère | HK581 | 25VI12-2B (Lady’s Island, South Carolina) | LC715262, OP297462, OP297516 |
| *Podocystis* cf *americana* Bailey | HK453 | 19X15-1A (Channel #5, Florida) | MH040320, MH040267, MH040240 |
| *Podocystis* cf *americana* Bailey | HK454 | 19X15-1B (Channel #5, Florida) | MG684360, MG684331, MG684301 |
| *Podocystis spathulata* (Shadbolt) Van Heurck | HK217 | ECT3733 (Pago Bay, Guam) | HQ912661, HQ912525, HQ912354 |
| *Podosira baldjickiana* Grunow | HK669 | GU7X-5 (University of Guam Marine Labs) | MZ546214, MZ542394, MZ542414 |
| *Porosira glacialis* (Grunow) E. Jorgensen | HK115 | CCMP668 | HQ912619, HQ912483, HQ912312 |
| *Proboscia* sp B.G. Sundstrom | HK300 | ECT3891 (St. George Island, Florida) | KC309540, KC309612, KC309683 |
| *Proboscia* sp B.G. Sundstrom | HK236 | ECT3845 (Ward Island, Texas) | KC309541, KC309613, KC309684 |
| *Proschkinia* cf *complanatula* (Hustedt ex Simonsen) D.G. Mann | HK553 | 24II18-1G (Half Moon Bay, California) | MK736943, MK757575, MK757579 |
| *Proschkinia vergostriata* Frankovich, Ashworth & M.J. Sullivan | HK549 | CC032217a (Loggerhead turtle, Florida) | MK736939, MK757571, N/A |
| *Proschkinia vergostriata* Frankovich, Ashworth & M.J. Sullivan | HK550 | ChelMyN 26V16 (Green Sea Turtle, Turtle Hospital, Marathon, Florida) | MK736940, MK757572, MK757576 |
| *Proschkinia vergostriata* Frankovich, Ashworth & M.J. Sullivan | HK551 | ChelMyN 26V16 (Green Sea Turtle, Turtle Hospital, Marathon, Florida) | MK736941, MK757573, MK757577 |
| *Proschkinia vergostriata* Frankovich, Ashworth & M.J. Sullivan | HK552 | ChelMyN 26V16 (Green Sea Turtle, Turtle Hospital, Marathon, Florida) | MK736942, MK757574, MK757578 |
| *Psammodictyon constrictum* (Gregory) Mann in Round, Crawford & Mann | HK440 | GU7X-7 (University of Guam Marine Lab, Guam) | KX981851, KX981830, KX981805 |
| *Psammodictyon constrictum* (Gregory) Mann in Round, Crawford & Mann | HK471 | Nate Site 1 (Kona, Hawaii) | MH040329, MH040278, MH040252 |
| *Psammodictyon pustulatum* (Voigt ex Meister) Lobban | UTKSA0298 | KSA2015-38 (Rabigh, Saudi Arabia) | MH063502, MH064134, MH064041 |
| *Psammodictyon* sp Mann | UTKSA0117 | KSA2015-30 (Markaz Al Shoaibah, Saudi Arabia) | MH063499, MH064131, MH064038 |
| *Psammodictyon* sp Mann | UTKSA0151 | KSA2015-37 (Rabigh, Saudi Arabia) | MH063500, MH064132, MH064039 |
| *Psammodictyon* sp Mann | UTKSA0280 | KSA2015-2 (Bhadur Resort, Saudi Arabia) | MH063501, MH064133, MH064040 |
| *Psammogramma vigoensis* Sato & Medlin | s0391 |  | KR048194, KR048215, KR048227 |
| *Psammoneis japonica* Sato, Kooistra & Medlin | HK299 | GU52-O (Outhouse Beach, Guam) | JN975250, JN975265, JN975279 |
| *Psammoneis japonica* Sato, Kooistra & Medlin | HK521 | DTort8 (Dry Tortugas, Florida) | MN473923, MN473940, MN473965 |
| *Psammoneis* *obaidii* Ashworth & Sabir | UTKSA0057 | SA12 (Markaz Al Shoaibah, Saudi Arabia) | KR059023, KR059022, KR059024 |
| *Psammoneis* sp Sato, Kooistra & Medlin | UTKSA0250 | KSA2015-42 (Rabigh, Saudi Arabia) | MH063517, MH064149, N/A |
| *Psammotaenia lanceolata* Ashworth, Ch. Li & Witkowski | HK316 | 10X10-2 (St. George Island, Florida) | JX413543, JX413560, JX413577 |
| *Pseudauliscus ralfsii* (Kitton ex Pritchard) Schmidt | HK330 | ECT3896 (Port Aransas Jetty, Texas) | KC309506, KC309580, KC309653 |
| *Pseudictyota bicorne* (Cleve) P.A. Sims & D.M. Williams | HK222 | ECT3821 (Ward Island, Texas) | HQ912664, HQ912528, HQ912357 |
| *Pseudictyota dubium* (Brightwell) P.A. Sims & D.M. Williams | HK199 | ECT3767 (Taeleyag Beach, Guam) | HQ912647, HQ912511, HQ912340 |
| *Pseudictyota dubium* (Brightwell) P.A. Sims & D.M. Williams | HK254 | ECT3838 (Long Beach, California) | KC309519, KC309592, KC309665 |
| *Pseudictyota dubium* (Brightwell) P.A. Sims & D.M. Williams | HK342 | ECT3888 (San Sebastian, Canary Islands) | KC309520, KC309593, KC309666 |
| *Pseudictyota dubium* (Brightwell) P.A. Sims & D.M. Williams | HK597 | Tokiane3 (Madagascar) | N/A, MK817341, N/A |
| *Pseudictyota reticulata* (Roper) P.A. Sims & D.M. Williams | HK281 | Pan-1 (STRI Station, Panama) | JX413554, JX413571, JX413588 |
| *Pseudictyota reticulata* (Roper) P.A. Sims & D.M. Williams | HK706 | Tokiane3 (Madagascar) | OP304792, OP297463, OP297517 |
| *Pseudictyota* sp P.A. Sims & D.M. Williams | HK047 | CCMP147 | HQ912572, HQ912436, N/A |
| *Pseudogomphonema* sp Medlin | HK560 | 25II18-1B (Garrapata State Park, California) | N/A, MW324624, MW324669 |
| *Pseudoleyanella lunata* Takano |  | NG0002 | MF001996, MF001977, MF001947 |
| *Pseudosolenia calcar-avis* (Schultze) Sundstrom | HK245 | Har-1 (HBOI Boat Dock, Florida) | KC309542, KC309614, KC309685 |
| *Pseudosolenia calcar-avis* (Schultze) Sundstrom | HK414 | 25II12-3A TX (Redfish Bay, Texas) | KJ577871, KJ577908, KJ577942 |
| *Pseudosolenia calcar-avis* (Schultze) Sundstrom | HK668 | WKOct2015 (Italy) | MZ546215, MZ542395, MZ542415 |
| *Pseudostaurosira elliptica* (Schumann) Edlund, Morales & Spaulding |  | s0398 | MF093080, HQ828191, MF092998 |
| *Pseudostaurosira madagascariensis* Ch. Li, Witkowski & Witak |  | SZCZCH755 | MF093065, MF092897, MF092983 |
| *Pseudostriatella oceanica* Sato, Mann & Medlin |  | s0384 | KR048197, KR048219, KR048231 |
| *Pteroncola* sp Holmes & Croll | UTKSA0078 | SA29 (Jeddah, Saudi Arabia) | MG684376, N/A, MG684316 |
| *Ralfsiella minima* (Grunow) P.A.Sims & D.M.Williams | HK336 | ECT3886 (Bald Head Island, North Carolina) | KC309510, KC309584, KC309657 |
| *Ralfsiella minima* (Grunow) P.A.Sims & D.M.Williams | HK337 | ECT3892 (St. George Island, Florida) | KC309511, KC309585, N/A |
| *Ralfsiella smithii* (Ralfs in Pritchard) P.A. Sims, D.M. Williams & Ashworth | HK224 | ECT3829 (Redfish Bay, Texas) | HQ912666, HQ912530, N/A |
| *Ralfsiella smithii* (Ralfs in Pritchard) P.A. Sims, D.M. Williams & Ashworth | HK331 | ECT3886 (Bald Head Island, North Carolina) | KC309488, KC309560, KC309632 |
| *Ralfsiella smithii* (Ralfs in Pritchard) P.A. Sims, D.M. Williams & Ashworth | HK703 | RO400m (Angola) | OP304793, OP297464, N/A |
| *Ralfsiella* sp P.A. Sims, D.M. Williams & Ashworth | HK341 | ECT3883 (Rainbow Harbor, Long Beach, California) | JX413555, JX413572, JX413589 |
| *Rhabdonema adriaticum* Kützing | HK370 | Coz-3 (Cozumel, Mexico) | JX401243, JX401261, JX401278 |
| *Rhabdonema arcuatum* (Lyngbye) Kützing | HK304 | ECT3898 (Pebble Beach, California) | JN975251, JN975266, JN975280 |
| *Rhabdonema* sp Kützing | HK369 | GU44AI-1 (Gab Gab Beach, Guam) | JX401242, JX401260, JX401277 |
| *Rhaphoneis amphiceros* (Ehrenberg) Ehrenberg | HK237 | ECT3828 (Redfish Bay, Texas) | HQ912673, HQ912537, KC309625 |
| *Rhaphoneis amphiceros* (Ehrenberg) Ehrenberg | HK373 | 25VI12-1A (Hunting Island, South Carolina) | KF701593, KF701602, KF701611 |
| *Rhizosolenia formosa* H. Peragallo | HK354 | ECT3896 (Port Aransas, Texas) | JX413557, JX413574, JX413591 |
| *Rhizosolenia imbricata* Brightwell | HK244 | Har-1 (HBOI Boat Dock, Florida) | KC309543, KC309615, KC309686 |
| *Rhizosolenia setigera* Brightwell | HK032 | CCMP1820 | HQ912561, HQ912425, HQ912255 |
| *Rhizosolenia setigera* Brightwell | HK268 | ECT3845 (Ward Island, Texas) | KC309544, KC309616, KC309687 |
| *Rhizosolenia setigera* Brightwell | HK449 | 25VI12-2A (Lady’s Island, South Carolina) | MZ546216, MZ542396, MZ542416 |
| *Rhizosolenia shrubsolei* Cleve | HK221 | ECT3821 (Ward Island, Texas) | HQ912663, HQ912527, HQ912356 |
| *Rhizosolenia shrubsolei* Cleve | HK619 | P1 12-8-13 (Pensacola Bay, Florida) | MZ546217, MZ542397, MZ542417 |
| *Rhoikoneis pagoensis* C.S. Lobban | HK419 | GU7X-7 (University of Guam Marine Lab, Guam) | KX981846, KX981825, KX981800 |
| *Rhoikoneis pagoensis* C.S. Lobban | UTKSA0128 | KSA2015-16 (Al-Nawras, Jeddah, Saudi Arabia) | MH063503, MH064135, N/A |
| *Rhoicosigma* sp Grunow | UTKSA0194 | KSA2015-22 (Markaz Al Shoaibah, Saudi Arabia) | MH063504, MH064136, MH064042 |
| *Rhoicosigma* sp Grunow | UTKSA0418 | KSA2016-12 (Bhadur Resort, Jeddah, Saudi Arabia) | OP304794, OP297465, N/A |
| *Rhoicosphenia abbreviata* (C.Agardh) Lange-Bertalot |  | CH030 | KJ011672, KJ011854, N/A |
| *Rhoicosphenia cf abbreviata* (C.Agardh) Lange-Bertalot |  | EWT2016.80 | KU965569, KU965580, N/A |
| *Rhopalodia contorta* Hustedt |  | L1299 (UTEX) | HQ912406, HQ912392, HQ912378 |
| *Rhopalodia gibba* (Ehrenberg) O. Müller |  |  | HQ912407, HQ912393, HQ912379 |
| *Rhopalodia* sp O. Müller | HK433 | 21IV14-4D (Rabbit Key Basin, Florida) | KX981843, KX981823, KX981799 |
| *Rhopalodia* sp O. Müller |  | ECT3678 (Tinago River, Guam) | HQ912405, HQ912391, HQ912377 |
| *Rossia* sp Voigt |  |  | EF151968, EF143281, N/A |
| *Roundia cardiophora* (Round) Makarova | HK219 | ECT3681 (Achang Reef, Guam) | KC284712, KC284708, KC284700 |
| *Schizostauron kajotkei* Dabek, Górecka & Witkowski |  | SZCZP32 | KT943595, KT943606, KT943619 |
| *Schizostauron kajotkei* Dabek, Górecka & Witkowski |  | SZCZP40 | KT943596, KT943607, KT943620 |
| *Schizostauron trachyderma* (F.Meister) Górecka, Riaux-Gobin & Witkowski |  | SZCZE1420 | MT982906, MT981072, MT981057 |
| *Schizostauron trachyderma* (F.Meister) Górecka, Riaux-Gobin & Witkowski |  | SZCZE1428 | MT982914, MT981080, MT981065 |
| *Schizostauron rawaii* Górecka, Ashworth, Sabir & Witkowski | UTKSA0141 | KSA2015-11 (Bhadur Resort, Saudi Arabia) | MH063505, MH064137, MH064043 |
| *Scoliopleura peisonis* Grunow | HK103 | FD13 (UTEX) | HQ912609, HQ912473, HQ912302 |
| *Sellaphora laevissima* (Kützing) D.G.Mann | THR4 |  | EF151981, EF143309, N/A |
| *Sellaphora minima* Grunow | TCC524 |  | KF959656, KF959642, N/A |
| *Sellaphora seminulum* (Grunow) D.G. Mann | TCC461 |  | KF959642, KC736613, N/A |
| *Seminavis robusta* D.B.Danielidis & D.G.Mann | HK492 | GU7X-7 (University of Guam Marine Laboratories, Guam) | MH040330, MH040279, MH040253 |
| *Seminavis* sp Mann | UTKSA0266 | KSA2015-47 (Duba, Saudi Arabia) | MW327194, MW324626, MW324670 |
| *Serratifera* *andersonii* Li, Dąbek & Wachnicka |  | SZCZP1059 | MF093090, MF092921, MF093007 |
| *Serratifera* *andersonii* Li, Dąbek & Wachnicka |  | SZCZP1183 | MF093070, MF092902, MF092988 |
| *Serratifera* cf *andersonii* Li, Dąbek & Wachnicka |  | SZCZM910 | MF093086, MF092917, MF093003 |
| *Serratifera* cf *andersonii* Li, Dąbek & Wachnicka |  | SZCZP693 | MF093087, MF092918, MF093004 |
| *Serratifera* cf *andersonii* Li, Dąbek & Wachnicka |  | SZCZP696 | MF093071, MF092903, MF092989 |
| *Serratifera* *brevis* Li & Ashworth | HK446 | Nate Site 1 (Kona, Hawaii) | KU851869, KU851880, KU851895 |
| *Serratifera clavata* Li, Tomczak & Witkowski |  | SZCZCH752 | N/A, KU851890, KU851905 |
| *Serratifera corallina* Li, Górecka & Kwon |  | SZCZE1544 | MF093137, MF092968, N/A |
| *Serratifera* *namibica* Li & Witkowski |  | SZCZCH585 | KU851876, KU851887, KU851902 |
| *Serratifera* *namibica* Li & Witkowski |  | SZCZP88 | MF093067, MF092899, MF092985 |
| *Serratifera* *nosybeana* Li, Witkowski & Riaux-Gobin |  | SZCZCH992 | KU851871, KU851882, KU851897 |
| *Serratifera parkii* Li & Ashworth | HK507 | TongYeongLNG (South Korea) | MF093125, MF092956, MF093040 |
| *Serratifera punctata* Sato & Li |  | s0386 | MF093082, MF092913, N/A |
| *Serratifera* sp Ashworth, Ch. Li & Witkowski |  | CCMP3169 | MF093129, MF092960, MF093044 |
| *Serratifera sourniae* Li, Riaux-Gobin & Witkowski |  | SZCZCH583 | KU851877, KU851888, KU851903 |
| *Serratifera sourniae* Li, Riaux-Gobin & Witkowski |  | SZCZE517 | MF093097, MF092928, MF093014 |
| *Serratifera sourniae* Li, Riaux-Gobin & Witkowski |  | SZCZE614 | MF093098, MF092929, MF093015 |
| *Serratifera sourniae* Li, Riaux-Gobin & Witkowski |  | SZCZM485 | MF093131, MF092962, MF093046 |
| *Serratifera sourniae* Li, Riaux-Gobin & Witkowski |  | SZCZM537 | MF093066, MF092898, MF092984 |
| *Serratifera takanoi* Sato & Li |  | s0308 | MF093081, MF092912, MF092999 |
| *Serratifera varisterna* Li, Ashworth & Witkowski | HK315 | 9X10-2 (Florida State University Marine Lab, Florida) | JX413542, JX413559, JX413576 |
| *Serratifera varisterna* Li, Ashworth & Witkowski |  | SZCZCH168 | KU851870, KU851881, KU851896 |
| *Serratifera varisterna* Li, Ashworth & Witkowski | HK424 | PackaryChannelPlankton (Mustang Island, Texas) | KU851868, KU851879, KU851894 |
| *Stauroforma rinceana* Meleder, Witkowski & Li |  | SZCZCH1603 | MF093138, MF092979, MF093062 |
| *Stauroneis acuta* W. Smith | HK059 | FD51 (UTEX) | HQ912579, HQ912443, HQ912272 |
| *Stauroneis anceps* Ehrenberg |  |  | AM502008, AM710475, N/A |
| *Stauroneis gracilior* Reichardt |  |  | AM501988, AM710454, N/A |
| *Stauroneis kriegeri* Patrick |  |  | AM501990, AM710456, N/A |
| *Stauroneis phoenicentron* (Nitzsch) Ehrenberg |  |  | AM502031, AM710498, N/A |
| *Staurophora* sp Mereschkowsky | HK503 | FLMan1 (manatee, Crystal River, Florida) | MH017644, MW324628, MW324672 |
| *Staurosira construens* Ehrenberg | HK071 | FD232 (UTEX) | HQ912587, HQ912451, HQ912280 |
| *Staurosirella pinnata* (Ehrenberg) Williams & Round | HK116 | CCMP330 (NCMA) | HQ912620, HQ912484, HQ912313 |
| *Staurotropis americana* Ashworth | HK442 | FishPassMangrove (Mustang Island, Texas) | KX981855, KX981834, KX981808 |
| *Staurotropis americana* Ashworth | HK443 | Coz4 (Cozumel, Mexico) | KX981854, KX981833, KX981807 |
| *Staurotropis seychellensis* (Giffen) Paddock | HK172 | ECT3721 (University of Guam Marine Lab, Guam) | KX981856, N/A, KX981809 |
| *Stellarima microtrias* (Ehrenberg) Hasle & Sims | HK108 | CCMP806 | HQ912614, HQ912478, HQ912307 |
| *Stenopterobia curvula* (W. Smith) Krammer |  | L541 (UTEX) | HQ912416, HQ912402, HQ912388 |
| *Stephanopyxis turris* (Greville & Arnott) Ralfs | HK213 | ECT3828 (Redfish Bay, Texas) | HQ912657, HQ912521, HQ912350 |
| *Sternimirus shandongensis* Witkowski & Li |  | SZCZCH968 | KT943637, KT943662, KT943696 |
| *Stictocyclus stictodiscus* (Grunow) R. Ross | HK269 | GU44AB-8 (Gab Gab Beach, Guam) | KC309507, KC309581, KC309654 |
| *Striatella unipunctata* (Lyngbye) Agardh | HK177 | ECT3648 (Asan Beach, Guam) | HQ912643, HQ912507, HQ912336 |
| *Striatella unipunctata* (Lyngbye) Agardh | HK318 | ECT3874 (Channel #5, Florida) | JX419383, JX419384, JX419385 |
| *Stricosus alfageehii* Sabir & Theriot | KSA0128 | SA12 (Markaz Al Shoaibah, Saudi Arabia) | MG684369, MG684340, MG684308 |
| *Stricosus alfageehii* Sabir & Theriot | UTKSA0077 | SA29 (Jeddah, Saudi Arabia) | MG684370, MG684341, MG684309 |
| *Stricosus alfageehii* Sabir & Theriot | UTKSA0242 | KSA2015-14 (Jeddah, Saudi Arabia) | MG684371, MG684342, MG684310 |
| *Stricosus blumbergii* Theriot & Ashworth | HK362 | 15VI11-2A (Baffin Bay, Texas) | JX401235, JX401253, JX401271 |
| *Stricosus cardinalii* Theriot & Lobban | HK516 | GU7Y-4 (University of Guam Marine Lab, Guam) | MG684372, N/A, MG684311 |
| *Stricosus gracilis* Theriot & Ashworth | HK515 | 21IV14-4E (Rabbit Key Basin, Florida) | MG684373, MG684343, MG684312 |
| *Stricosus harrisonii* Lobban & Theriot | HK363 | GU44AI (Gab Gab Beach, Guam) | JX401236, JX401254, JX401272 |
| *Stricosus madanii* Sabir & Theriot | KSA0171 | SA29 (Jeddah, Saudi Arabia) | MG684374, MG684344, MG684313 |
| *Stricosus madanii* Sabir & Theriot | UTKSA0065 | SA29 (Jeddah, Saudi Arabia) | MG684375, MG684345, MG684314 |
| *Stricosus navarroensis* Theriot & Ashworth | HK514 | PR1 (San Juan, Puerto Rico) | MG684368, MG684339, MG684307 |
| *Surirella* cf *fastuosa* (Ehrenberg) Ehrenberg |  | SZCZCH189 | KT943629, KT943655, KT943688 |
| *Surirella minuta* Van Heurck |  | FD320 (UTEX) | HQ912658, HQ912522, HQ912351 |
| *Surirella ovata* Kützing | HK214 | L1241 (UTEX) | HQ912658, HQ912522, HQ912351 |
| *Surirella splendida* (Ehrenberg) Kützing |  |  | HQ912415, HQ912401, HQ912387 |
| *Surirella* sp Turpin | HK717 | GU21AO-3 (Saluglula Pools, Inarajan, Guam) | OP304795, OP297466, OP297518 |
| *Surirella* sp Turpin | UTKSA0299 | KSA2015-2 (Bhadur Resort, Saudi Arabia) | MH063507, MH064139, MH064045 |
| *Synedra famelica* Kützing | HK072 | FD255 (UTEX) | HQ912588, HQ912452, HQ912281 |
| *Synedra ulna* (Nitzsch) Ehrenberg | HK075 | FD404 (UTEX) | HQ912590, HQ912454, HQ912283 |
| *Synedropsis hyperborea* (Grunow) Hasle, Medlin & Syvertsen | HK117 | CCMP1423 (NCMA) | HQ912621, HQ912485,  HQ912314 |
| *Synedropsis* cf *recta* Hasle, Medlin & Syvertsen | HK110 | CCMP1620 (NCMA) | HQ912616, HQ912480, HQ912309 |
| *Synedrosphenia crystallina* (Agardh) Lobban & Ashworth |  | ND3 0722 E | MF555728, MF578745, MF578744 |
| *Synedrosphenia* sp (H.Peragallo) Azpeitia | HK589 | GU44AV8 (Gab Gab Beach, Guam) | MZ546199, MZ542378, N/A |
| *Tabellaria flocculosa* (Roth) Kützing | HK065 | FD133 (UTEX) | HQ912584, HQ912448, HQ912277 |
| *Tabularia* cf *tabulata* (Agardh) Snoeijs | HK109 | CCMP846 (NCMA) | HQ912615, HQ912479, HQ912308 |
| *Talaroneis posidoniae* Kooistra & De Stefano | WK59 |  | AY216905, KR048214, KR048226 |
| *Tenuicylindrus belgicus* (Meunier) Nanjappa & Zingone |  | SZN-B739 | KC814840, KC814844, KC814850 |
| *Terpsinoë americana* (Bailey) Ralfs | HK415 | 25VI12-1C SC (Hunting Island, South Carolina) | KJ577872, KJ577909, KJ577943 |
| *Terpsinoë musica* Ehrenberg | HK273 | NHOP43 (Brackenridge Field Lab, Texas) | HQ912682, HQ912546, HQ912370 |
| *Terpsinoë musica* Ehrenberg | HK544 | CX28VIII07 (Guam) | MN917246, MN917223, MN917235 |
| *Terpsinoë musica* Ehrenberg | HK545 | BallenaEstRock (Costa Rica) | MN917247, MN917224, MN917236 |
| *Tetracyclus* sp Ralfs | HK416 | B12 (Lake Baikal, Russia) | KJ577873, KJ577910, KJ577944 |
| *Tetramphora* *chilensis* (Hustedt) Stepanek & Kociolek |  | AMPH132 | KU665638, KU665639, KU665640 |
| *Thalassionema* cf *bacillare* (Heiden) Kolbe | HK361 | ECT3929 (Gulf of Mexico, Texas) | JX401234, JX401252, JX401270 |
| *Thalassionema frauenfeldii* (Grunow) Tempère & Peragallo | HK372 | 25VI12-1A (Hunting Island, South Carolina) | KF701592, KF701601, KF701610 |
| *Thalassionema* cf *nitzschioides* (Grunow) Mereschkowsky | HK360 | ECT3929 (Gulf of Mexico, Texas) | JX401233, JX401251, JX401269 |
| *Thalassionema* sp Grunow ex Mereschkowsky | HK713 | 14IV18-1B (Ochlockonee Bay, Florida) | OP304796, OP297467, OP297519 |
| *Thalassionema* sp Grunow ex Mereschkowsky | HK714 | 14IV18-2B (Carrabelle, Florida) | OP304797, OP297468, OP297520 |
| *Thalassionema* sp Grunow ex Mereschkowsky | HK715 | 14IV18-2B (Carrabelle, Florida) | OP304798, OP297469, OP297521 |
| *Thalassiosira pseudonana* Hasle & Heimdal | HK008 | CCMP1335 | HQ912555, HQ912419, HQ912249 |
| *Toxarium hennedyanum* (Gregory) Pelletan | HK210 | ECT3802 (Gab Gab Beach, Guam) | HQ912654, HQ912518, HQ912347 |
| *Toxarium hennedyanum* (Gregory) Pelletan | HK220 | ECT3648 (Asan Beach, Guam) | HQ912662, HQ912526, HQ912355 |
| *Toxarium hennedyanum* (Gregory) Pelletan | HK678 | GU44BR-5 (Gab Gab Beach, Guam) | OP304799, OP297470, OP297522 |
| *Toxarium hennedyanum* (Gregory) Pelletan | UTKSA0164 | KSA2015-49 (Duba, Saudi Arabia) | OP304800, OP297471, OP297523 |
| *Toxarium undulatum* Bailey |  | WK44 | AF525668, N/A, N/A |
| *Trachyneis* sp P.T. Cleve | HK439 | SantaRosaCor.green (Costa Rica) | KX981845, KX981824, N/A |
| *Triceratium robertsianum* Greville | HK700 | 14IV18-5A (St. George Island, Florida) | OP304801, OP297472, OP297524 |
| *Triceratium spinosum* Bailey | UTKSA0420 | KSA2016-37 (Duba, Saudi Arabia) | OP304802, OP297473, OP297525 |
| *Triceratium* sp Ehrenberg | HK702 | RO400m (Angola) | OP304803, OP297474, N/A |
| *Trieres mobiliensis* (Bailey) Ashworth & Theriot | HK204 | ECT3829 (Redfish Bay, Texas) | JX413549, JX413566, JX413583 |
| *Trieres mobiliensis* (Bailey) Ashworth & Theriot | HK227 | ECT3834 (Alamitos Bay, California) | KC309654, KC309573, KC309646 |
| *Trieres mobiliensis* (Bailey) Ashworth & Theriot | HK251 | Har-1 (Harbor Branch Oceanographic Institute, Florida) | KC309500, KC309574, KC309647 |
| *Trieres regia* (M.Schultze) Ashworth & Theriot | HK290 | 27II10-3C (Port Aransas, Texas) | KC309502, KC309576, KC309649 |
| *Trieres regia* (M.Schultze) Ashworth & Theriot | HK322 | Har-1 (Harbor Branch Oceanographic Institute, Florida) | KC309501, KC309575, KC309648 |
| *Trieres sinensis* (Greville) Ashworth & Theriot | HK037 | CCMP1815 | HQ912564, HQ912428, HQ912258 |
| *Trieres sinensis* (Greville) Ashworth & Theriot | HK323 | ECT3886 (Bald Head Island, North Carolina) | KC309503, KC309577, KC309650 |
| *Trieres* sp Ashworth & Theriot | HK406 | 25II12-4 (Port Aransas, Texas) | KJ577863, KJ577900, KJ577934 |
| *Trieres* sp Ashworth & Theriot | HK705 | Azo42b (Azores) | OP304804, N/A, N/A |
| *Trigonium formosum* (Brightwell) Frenguelli | HK200 | ECT3689 (Saluglula Pools, Guam) | HQ912648, HQ912512, HQ912341 |
| *Trigonium formosum* (Brightwell) Frenguelli | HK258 | University of Hawaii, Manoa culture | JX413550, JX413567, JX413584 |
| *Trigonium formosum f. quadrangularis* (Greville) T.V. Desikachary & Sreelatha | CX17 | ECT3671 (Haputo Point, Guam) | KC309518, N/A, KC309664 |
| *Tryblionella apiculata* Gregory | HK087 | FD465 (UTEX) | HQ912600, HQ912464, HQ912293 |
| *Tryblionella gaoana* Witkowski & Ch. Li | SZCZCH97 |  | KT943638, KT943683, KT943697 |
| *Ulnaria ulna* (Nitzsch) P. Compère |  | WK36 | MG684361, MG684332, MG684302 |
| *Urosolenia eriensis* (H.L. Smith) Round & Crawford | HK054 | Y98-8 (Yellowstone Lake, Wyoming) | HQ912577, HQ912441, HQ912270 |
| unidentified hemidiscoid | HK402 | ECT3886 (Bald Head Island, North Carolina) | KJ577859, KJ577896, KJ577930 |
| unidentified hemidiscoid | HK701 | ECT3886 (Bald Head Island, North Carolina) | OP304805, OP297475, OP297526 |
| unidentified hemidiscoid |  | AJA027-32 | OP304806, OP297476, OP297527 |
| unidentified monoraphid | HK380 | ECT3899 (Pacific Grove, California) | KJ577839, KJ577874, KJ577911 |
| unidentified monoraphid | HK427 | BallenaEstRock (Costa Rica) | KU179136, KU179121, KU179148 |
| unidentified monoraphid | UTKSA0152 | KSA2015-37 (Rabigh, Saudi Arabia) | MH063509, MH064141, MH064047 |
| unidentified monoraphid | UTKSA0158 | KSA2015-37 (Rabigh, Saudi Arabia) | MH063510, MH064142, N/A |
| unidentified naviculoid | UTKSA0247 | KSA2015-5 (Bhadur Resort, Saudi Arabia) | MH063512, MH064144, MH064049 |
| unidentified naviculoid | HK497 | 23X15-5B (Harbor Branch Oceanographic Institute dock, Florida) | MH063511, MH064143, MH064048 |
| unidentified opephoroid |  | s0402 | MF093084, MF092915, MF093001 |
| unidentified opephoroid |  | s0403 | MF093085, MF092916, MF093002 |
| unidentified stauroneid | UTKSA0220 | KSA2015-7 (Bhadur Resort, Saudi Arabia) | MH063513, MH064145, MH064050 |
| unidentified tabularioid | HK509 | 24IV14-4B (Rabbit Key Basin, Florida) | MG684377, MG684347, MG684317 |
| unidentified tabularioid | HK513 | Azo42a (Azores) | MG684378, MG684348, MG684318 |
