# Supplementary figures and images for: ﻿Revision of Ardissoneaceae (Bacillariophyta, Mediophyceae) from Micronesian populations, with descriptions of two new genera, Ardissoneopsis and Grunowago, and new species in Ardissonea, Synedrosphenia and Climacosphenia
Source: PhytoKeys. 2022 Sep 21;208:103–84. doi: 10.3897/phytokeys.208.89913 (PMC9848972; doi:10.3897/phytokeys.208.89913)

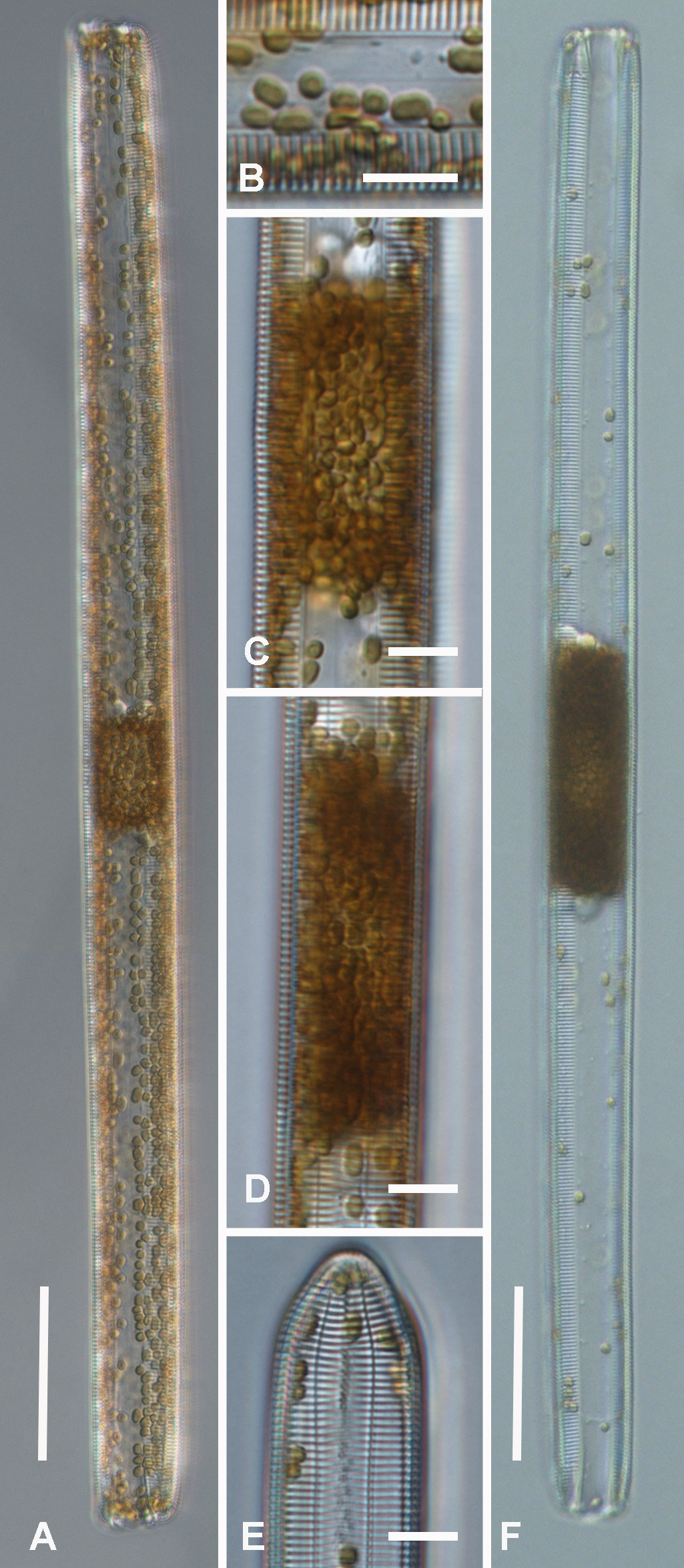

Supplement: Supplementary material 3 — Figure S1. Plastids in a living cell of Ardissoneaformosa showing movement from dispersed (peristrophy) to concentrated around the nucleus (karyostrophy) [file phytokeys-208-103_article-89913__-s003.jpg]

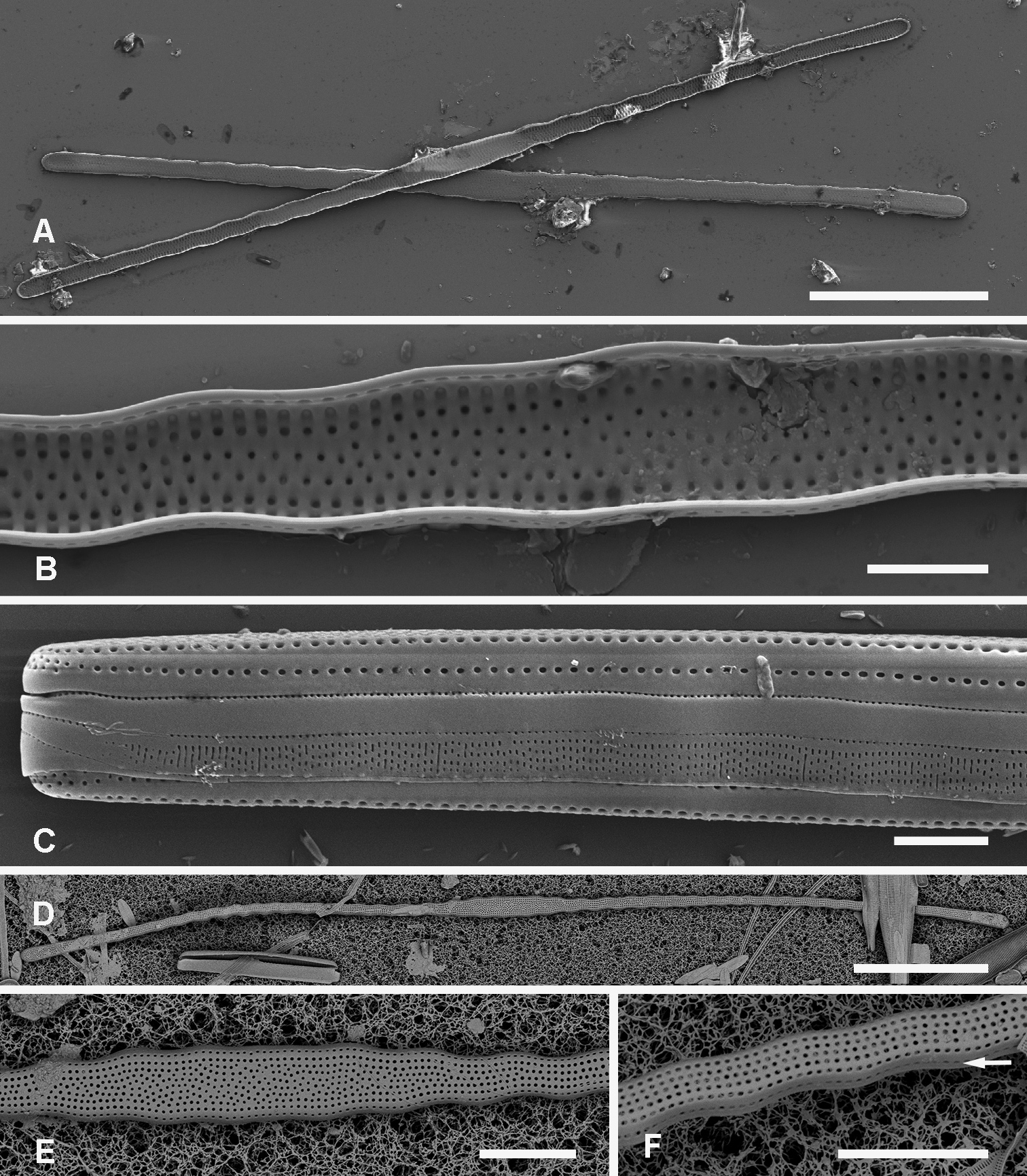

Supplement: Supplementary material 4 — Figure S2. Toxarium sp. from Guam [file phytokeys-208-103_article-89913__-s004.jpg]
