# Supplementary material for: ﻿Revision of Ardissoneaceae (Bacillariophyta, Mediophyceae) from Micronesian populations, with descriptions of two new genera, Ardissoneopsis and Grunowago, and new species in Ardissonea, Synedrosphenia and Climacosphenia
Source: PhytoKeys. 2022 Sep 21;208:103–84. doi: 10.3897/phytokeys.208.89913 (PMC9848972; doi:10.3897/phytokeys.208.89913)

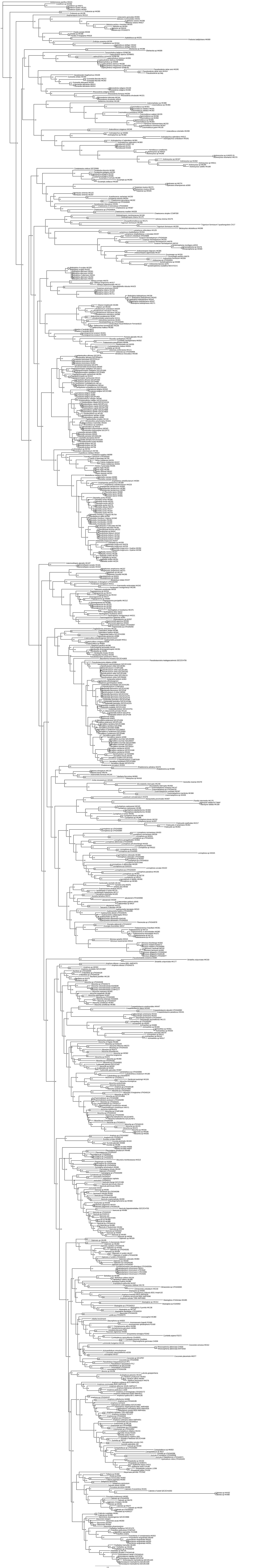

Supplement: Supplementary material 6 — Figure S4. Molecular phylogenetic tree illustrating the results of the IQTREE Maximum Likelihood analysis of the 3-gene dataset [file phytokeys-208-103_article-89913__-s006.pdf]
